# Defining a tandem repeat catalog and variation clusters for genome-wide analyses

**DOI:** 10.1101/2024.10.04.615514

**Published:** 2025-10-16

**Authors:** Ben Weisburd, Egor Dolzhenko, Mark F. Bennett, Matt C. Danzi, Isaac R. L. Xu, Hope Tanudisastro, Adam English, Laurel Hiatt, Tom Mokveld, Guilherme De Sena Brandine, Nehir Edibe Kurtas, Helyaneh Ziaei Jam, Harrison Brand, Stephan Züchner, Melissa Gymrek, Harriet Dashnow, Michael A. Eberle, Heidi L. Rehm

**Affiliations:** 1. Program in Medical and Population Genetics, Broad Center for Mendelian Genomics, Broad Institute of MIT and Harvard, Cambridge, MA, USA; 2. Center for Genomic Medicine, Massachusetts General Hospital, Harvard Medical School, Boston, MA, USA; 3. Pacific Biosciences, 1305 O’Brien Drive, Menlo Park, CA, USA; 4. Population Health and Immunity Division, Walter and Eliza Hall Institute of Medical Research, Parkville, Victoria, Australia; 5. Department of Medical Biology, University of Melbourne, Parkville, Victoria, Australia; 6. Epilepsy Research Centre, Department of Medicine, University of Melbourne, Austin Health, Heidelberg, Victoria, Australia; 7. Dr. John T. Macdonald Foundation Department of Human Genetics and John P. Hussman Institute for Human Genomics, University of Miami Miller School of Medicine, Miami, FL, USA; 8. Centre for Population Genomics, Garvan Institute of Medical Research, Sydney, NSW, Australia; 9. Centre for Population Genomics, Murdoch Children’s Research Institute, Melbourne, VIC, Australia; 10. Faculty of Medicine and Health, University of New South Wales, Sydney, NSW, Australia; 11. Faculty of Medicine and Health, University of Sydney, Sydney, NSW, Australia; 12. Human Genome Sequencing Center, Baylor College of Medicine, Houston, TX, USA; 13. University of Utah, Department of Human Genetics, Salt Lake City, UT; 14. Department of Computer Science and Engineering, University of California San Diego, La Jolla, CA; 15. Department of Biomedical Informatics, University of Colorado Anschutz Medical Campus, Aurora, CO

## Abstract

Tandem repeat (TR) catalogs are important components of repeat genotyping studies as they define the genomic coordinates and expected motifs of all TR loci being analyzed. In recent years, genome-wide studies have used catalogs ranging in size from fewer than 200,000 to over 7 million loci. Where these catalogs overlapped, they often disagreed on locus boundaries, hindering the comparison and reuse of results across studies. Now, with multiple groups developing public databases of TR variation in large population cohorts, there is a risk that, without sufficient consensus in the choice of locus definitions, the use of divergent repeat catalogs will lead to confusion, fragmentation, and incompatibility across future resources.

In this paper, we compare existing TR catalogs and discuss desirable features of a comprehensive genome-wide catalog. We then present a new, richly annotated catalog designed for large-scale analyses and population databases. Our catalog stratifies TRs into two groups: 1) isolated TRs suitable for repeat copy number analysis using short-read or long-read data and 2) so-called variation clusters that contain TRs within wider polymorphic regions that are best studied through sequence-level analysis. To define variation clusters, we present a novel algorithm that leverages long-read HiFi sequencing data to group repeats with surrounding polymorphisms. We show that the human genome contains at least 25,000 complex variation clusters, most of which span over 120 bp and contain five or more TRs. Resolving the sequence of entire variation clusters instead of individually genotyping constituent TRs leads to a more accurate analysis of these regions and enables us to profile variation that would have been missed otherwise.

## Introduction

Tandem repeats (TRs) are nucleotide sequences composed of consecutive repetitions of a shorter motif (also called a repeat unit). TRs with motif size of 6 bp or less are called short tandem repeats (STRs) while those with longer motifs are called variable number tandem repeats (VNTRs). These sequences can be perfect repeats of a single motif - for example CAG CAG CAG CAG, or they can contain interruptions - as in CAG CAG CAA CAG. Over the past fifteen years, wide-ranging biological studies have used sequencing data to investigate many aspects of TRs including mutation rates,^1–4^ effects on gene expression,^5^ splicing,^6^ and disease risk.^7,8^ With the exception of several specialized methods for identifying *de novo* variation,^9–11^ most TR genotyping tools^12–21^ involved in these studies require the user to provide a TR catalog which specifies the reference coordinates and motifs of each TR locus to genotype - making the choice of catalog a key factor in the overall sensitivity of a study.^22–24^

Researchers have typically generated TR catalogs using tools like Tandem Repeats Finder (TRF)^25^ that detect repetitive sequences by scanning the reference genome. Although a reference-based approach is able to identify a wide range of TR loci, it can miss polymorphic repeats that are too short or absent from the reference genome. Furthermore, methods like TRF are highly sensitive to input parameters, with some commonly-used settings yielding fewer than 1 million mostly perfect TRs, while other settings produce over 10 million TRs that contain many sequence interruptions. An alternative approach, which we call the cohort-driven strategy, detects TR variation directly from the sequencing data^9,10,26^ or haplotype-resolved assemblies.^27,28^ The cohort-driven strategy not only prioritizes polymorphic loci, but can also capture TRs that are not well represented in the reference genome and hence missed by the reference-based approach.

In addition to the different strategies for identifying TR loci, another important aspect of catalog design is the definition of locus boundaries. Since naturally occurring repeat sequences can contain interruptions or consist of multiple arrays of highly similar motifs, defining the starts and ends of TR loci in the reference genome is often subject to ambiguity. Yet, even small, seemingly inconsequential differences in how locus boundaries are specified within different catalogs, resources, or reference materials can lead to real issues with interpretation of TR genotypes. For example, the *PABPN1* locus has two commonly used definitions. With narrower locus boundaries, the pathogenic threshold is 8 GCGs, while with wider boundaries, it becomes 12 GCGs, creating the potential for inadvertent errors in the diagnosis of autosomal dominant oculopharyngeal muscular dystrophy (OPMD) if the genotype (number of repeats) is interpreted without awareness of which locus definition was used when running the genotyping tool (Supplementary Information).

The selection of TR loci and the specification of their boundaries can influence the suitability of a catalog for two different kinds of downstream analysis approaches. The first approach focuses primarily on the quantification of repeat copy numbers and using them to detect outlier expansions or perform association tests with other variables like gene expression. The second approach involves sequence-level analysis of TR regions in order to characterize motif composition, interruption patterns, and properties of flanking sequences. Most existing TR genotyping tools, especially those designed for short-read data, only report repeat copy numbers and not allele sequences.^12,13^ They are unable to resolve the full sequence-level variations of complex regions such as nested repeats and repeats surrounded by other repeats or structural variants.^29^ Additionally, these tools work best with narrow locus definitions that contain only perfect or nearly-perfect repeat sequences.^28^ Therefore, catalogs designed for repeat copy number analysis benefit from setting narrow locus boundaries, and flagging or excluding TRs within complex regions. On the other hand, the latest generation of tools^14,15,21^ can report allele sequences in addition to repeat copy numbers, and so enables sequence-level analysis both for isolated TRs as well as more complex repeat regions.^30,31^ Examples of TR variation that especially benefit from sequence-level analysis include the recently identified polymorphic region associated with the stability of the FGF14 repeat,^32^ a single base pair insertion in a homopolymer located within a *MUC1* VNTR that causes autosomal dominant tubulointerstitial kidney disease (ADTKD), and TRs like the *RFC1* locus whose pathogenicity is affected by sequence composition changes.^33,34^ Such loci frequently correspond to fragments of perfect tandem repeats punctuated by insertions, deletions, and substitutions in and around the repeat sequence.

Taken together, these observations highlight the challenges of creating genome-wide TR catalogs suitable for population studies, especially multi-center studies employing different computational tools and sequencing technologies. To help address these challenges, we introduce a new genome-wide repeat catalog. We employ both cohort-based and reference-based TR identification approaches to capture a comprehensive set of loci, including those that harbor common or rare variation. To simplify adoption, we share the catalog in the different formats used by popular TR analysis tools for short- and long-read data. Our catalog also stratifies TRs into two groups: isolated TRs suitable for repeat copy number analysis and variation clusters that contain TRs surrounded by polymorphic sequence. Due to their complexity, variation clusters are best studied through sequence level analysis, particularly using long-read data. To perform this stratification we introduce a new method for profiling population-scale variation around TRs and other regions of the genome. Lastly, we share the trexplorer.broadinstitute.org web portal to enable interactive online exploration of the loci and variation clusters in our catalog. Users can view TR population allele frequency distributions, visualize locus boundaries, and filter TRs by various criteria such as genomic interval, gene name, repeat motif, and/or polymorphism rate. Among other things, the filtering functionality allows users to define a smaller subset of the catalog that is most relevant to their particular study objectives, and then export it in the file format required as input to their preferred TR genotyping tool.

## Results

### Existing tandem repeat catalogs

**Figure 1.**
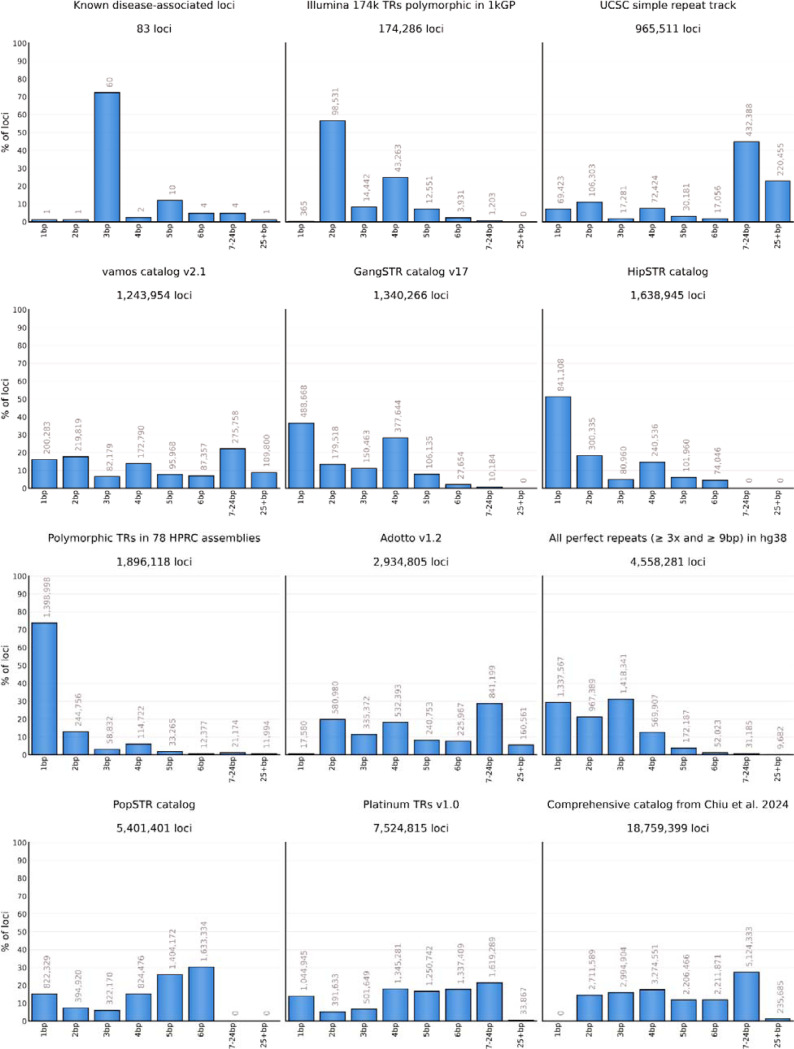
Comparison of motif size distributions across TR catalogs. The x-axis represents motif sizes. Each bar is labeled with the number of loci it contains.

Widely-used TR catalogs differ substantially in their core attributes such as their total number of loci, range of included motif sizes, and repeat purity (Table 1, Figure 1, Figures S1, S2, Methods). Strikingly, pairwise comparison of eleven TR catalogs showed that every pair differed in over half of their TR definitions (Figure S1). Many of these catalogs were aimed at different study objectives, tools and sequencing technologies, and so used different minimum repeat length and purity thresholds when selecting TR loci for inclusion. Also, they employed distinct approaches to define locus boundaries. For example, the recently-released Adotto v1.2, Platinum TRs v1.0, and the Chiu et al. 2024 catalogs contain many long imperfect repeats and include non-repetitive flanking sequences. Such locus definitions are compatible with existing tools for genotyping TRs in long reads, but cannot be accurately genotyped with existing tools for short-read sequencing data. On the other hand, older catalogs like GangSTR v17 and Illumina 174k define narrow locus boundaries around mostly perfect TR sequences and so are suitable for both short-read and long-read analyses. However, for technical or historical reasons, these catalogs have relatively low sensitivity as measured by their degree of overlap with our test set of true positive polymorphic STR loci with 3–6 bp motifs (Methods).

### Defining a genome-wide catalog

To incorporate the advantages of both reference-based and cohort-driven approaches, we collected loci from three cohort-driven catalogs and one reference-based catalog (Table 2). Where multiple source catalogs contained different definitions of the same TR locus (Methods), the higher prioritized definition - as described in Table 2 - was incorporated. To preserve compatibility of our locus definitions with those of existing resources, we prioritized widely-used catalogs above those from the new TR sources introduced in this paper. First, we included 63 disease-associated loci, 10 of their adjacent repeats, as well as 10 candidate TR loci that have been historically included in rare disease analyses. These loci have been the focus of extensive research,^35,36^ and many have well established, widely-used locus definitions which we incorporated here. Next, we added 174,244 loci from the Illumina catalog of polymorphic TRs which was created in 2021 by analyzing signals of TR variation within short-read sequencing data from 2,504 individuals in the 1000 Genomes Project.^26^ Prioritizing this catalog above catalogs 3 and 4 (Table 2) ensured compatibility with the locus boundary definitions used in prior studies and resources.^37^

To ensure comprehensive inclusion of perfect repeat sequences, we identified all TRs in hg38 that spanned at least 3 copies of a 3–1000 bp motif, at least 5 copies of a dinucleotide motif, or at least 9 copies of a homopolymer motif (Methods). This contributed another 4,391,197 loci to our catalog. Our focus on perfect repeats was motivated by their higher mutation rates compared to interrupted repeats, their importance to TR-associated disease mechanisms,^38^ as well as their higher overall genotyping accuracy in short-read analyses.^13^ We chose a ≥ 3x threshold to match several known disease-associated loci such as *C9orf72* that span only three repeats in the hg38 reference. Additionally, this threshold represented a favorable trade-off between sensitivity and specificity in terms of capturing as many polymorphic TR loci as possible while not including too many loci overall (Fig. S2 in Weisburd et al. 2023).^28^

Finally, we incorporated polymorphic TRs detected using 78 haplotype-resolved T2T assemblies from the Human Pangenome Reference Consortium (HPRC) and Human Genome Structural Variation Consortium (HGSVC). The specialized method we employed for this (Methods) allowed us to complement the previous three catalogs by capturing polymorphic loci that had fewer than three repeats in the reference, or that contained single base interruptions like those observed at known disease-associated loci such as *ZIC3*, *ATXN1, and FXN*. This approach contributed a further 297,519 TRs to the final catalog.

The resulting catalog contained 4,863,041 TRs that collectively spanned 2.1% of the hg38 reference. 4,803,366 of these (98.8%) were STRs, including 1,567,337 homopolymers (32.2%), 978,972 dinucleotide repeats (20.1%), 1,432,117 trinucleotide loci (29.4%), 590,787 loci with 4bp motifs (12.1%), 177,422 loci with 5bp motifs (3.6%), and 56,731 loci with 6bp motifs (1.2%). 59,675 were VNTRs (1.2%): 43,996 (0.9%) had 7 to 24 bp motif sizes and 15,679 (0.3%) had 25bp to 833bp motifs. Intersecting the catalog with Gencode v46 gene annotations and taking the most significant gene region based on the following priority: coding region (CDS), 5’ untranslated region (UTR), 3’ UTR, exon, intron, promoter, showed that 2,687,339 (55.3%) TRs in the catalog were intronic, 1,950,456 (40.1%) were intergenic, 56,725 (1.2%) overlapped promoters, 57,198 overlapped 3’ UTRs (1.2%), 54,883 (1.1%) overlapped exons of non-coding genes, 39,872 (0.8%) overlapped coding regions, and 16,568 (0.3%) overlapped 5’ UTRs.

### Analysis of variation clusters

**Figure 2:**
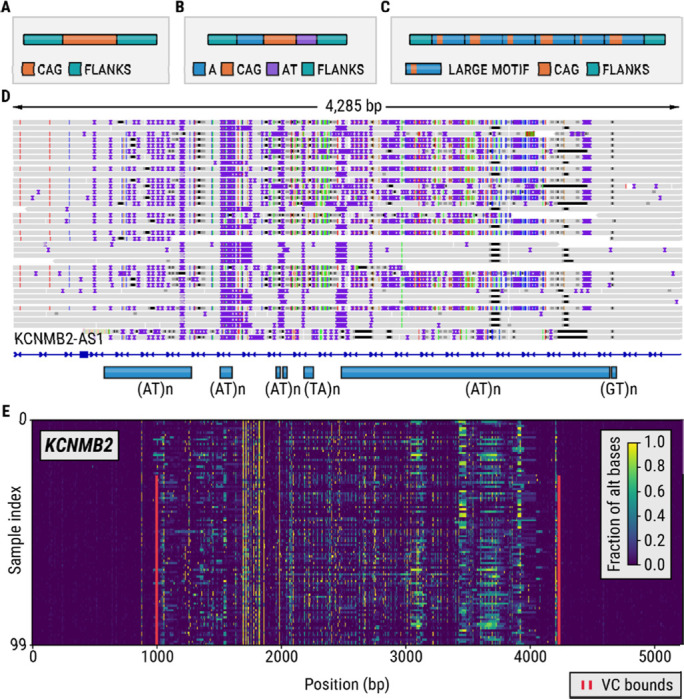
(A) A TR surrounded by non-repetitive flanks. (B) A TR with a CAG motif surrounded by an A homopolymer and AT dinucleotide repeat. (C) Nested TRs. (D) HiFi reads originating from the HG002 sample spanning a variation cluster located in an intron of the *KCNMB2* gene. (E) A variation plot depicting the *KCNMB2* locus in 100 long-read HiFi samples.

Some tandem repeats (TRs) are located within regions that have high rates of polymorphism and hence often differ significantly from the reference genome (Figure 2 A-C). We refer to such regions as variation clusters (VCs). Because most methods for TR analysis assume that the regions surrounding the TR closely resemble the reference genome, TRs located within variation clusters may be prone to analysis errors. For example, consider (AT)n repeats located in a variation cluster in the *KCNMB2* gene (Figure 2D). In the individual shown, the flanks of these TRs significantly differ from the reference genome, making it difficult to locate the exact boundaries or separate one repeat from the other repeats located within this region. This issue can be avoided if we locate the boundaries of the entire VC and then determine its full-length allele sequences in each sample with tools like TRGT^15^ or LongTR.^14^ Then the resulting sequences can be either analyzed directly or their repeat content can be characterized with existing methods,^15^ making it possible to analyze VCs together with isolated TRs.

**Figure 3.**
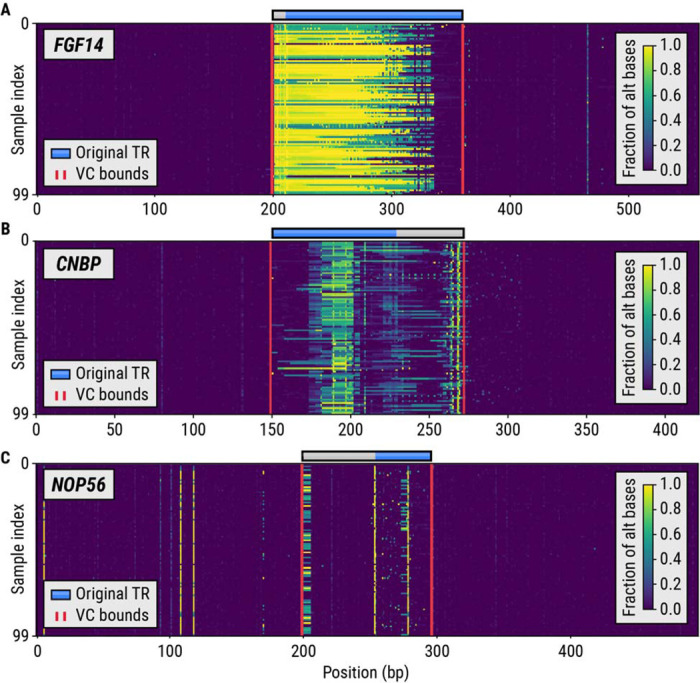
Variation plots showing the genetic variation in 100 HiFi HPRC samples around known pathogenic repeats in the (A) *FGF14*, (B) *CNBP*, and (C) *NOP56* genes. The blue bars denote the boundaries of the original repeat region while the gray bars depict the extension of the repeat region to a full-length variation cluster.

We developed a method, vclust, that when given a set of TR regions and aligned sequencing data from multiple samples can identify the bounds of variation clusters around each TR (Methods). We first used vclust to characterize variation across 63 loci containing known disease-associated TRs in 100 HiFi WGS HPRC samples. Our method identified variation clusters around eight of these TRs located in the *ATXN8OS*, *FGF14*, *NOP56*, *CNBP*, *HTT*, *BEAN1*, *LRP12*, and *EIF4A3* genes. Variation around most of these TRs has already been identified: the *ATXN8OS*, *NOP56*, *CNBP*, and *HTT* TRs contain adjacent repeats that contribute to variation in their flanks. A common insertion flanking the *FGF14* TR was recently found to have a stabilizing effect on this repeat.^32^ As anticipated, the vclust analysis incorporated the site of this insertion into the *FGF14* variation cluster (Figure 3A). The *CNBP* and *NOP56* repeats were the only two known pathogenic TRs whose variation clusters extended the original TR region by more than 40 bp. The *CNBP* VC (Figure 3B) includes two polymorphic repeats with CAGA and CA motifs while the *NOP56* VC (Figure 3C) contains a common deletion located less than 50 bp away from the repeat boundary. *BEAN1*, *LRP12*, and *EIF4A3* VCs were caused by the presence of small indels adjacent to these repeats.

**Figure 4.**
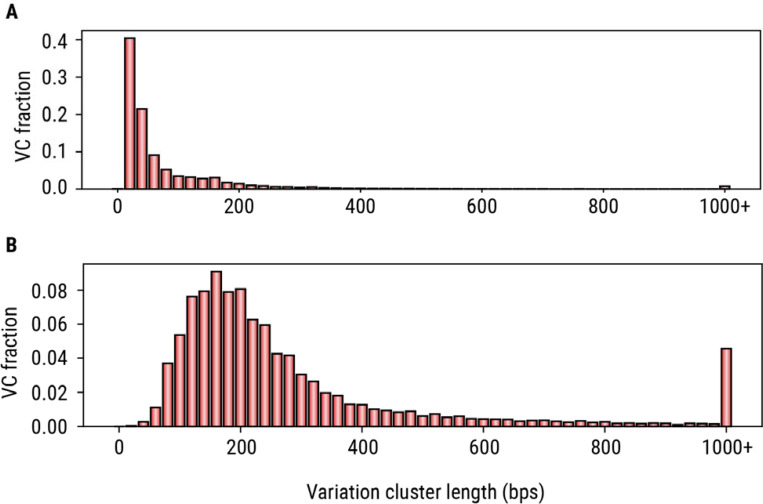
Size distributions of variation clusters. (A) Distribution of the differences between variation cluster length and the length of the original TR region that seeded it. (B) Distribution of lengths of complex variation clusters in the reference genome.

Next, we applied the variation cluster analysis to our catalog of 4,863,043 TRs. This analysis yielded 273,112 VCs that cumulatively overlapped 744,458 TRs, with 66% of VCs overlapping two or more TRs. About 18% of VCs (N=50,557) spanned over 120 bp in the reference (Figure 4A), a length at which exact genotyping with short-read data (30× 150bp WGS) becomes difficult. To demonstrate the benefits of genotyping complete variation clusters instead of constituent TRs, we compared the accuracy of allele sequences derived by genotyping entire VCs to the accuracy of alleles derived by genotyping the individual TRs in a long-read HiFi sequencing dataset from the HG002 sample. We defined accuracy as base-level concordance with a highly accurate assembly of the same genome ^39,40^ (Methods). The allele concordance rate increased from 79.43% for TRs (87.30% accepting off-by-one errors as concordant calls) to 90.58% (95.98%) for the corresponding VCs. The concordance rate was 96.21% (98.50%) for isolated TRs, demonstrating that individual TRs within VCs were a major source of genotyping errors. To further investigate VCs that contain multiple repeat tracts, we identified 24,867 VCs each containing five or more constituent TRs. We refer to these variation clusters as complex VCs. Complex VCs cumulatively contain 254,879 TRs, their median length is 208 bp and 86% exceed 120 bp (Figure 4B). For example, a VC in the *KCNMB2* gene (Figure 2E) spans over 3 Kbp in the reference genome and contains 135 TRs, including 116 TRs with either AT motifs.

**Figure 5.**
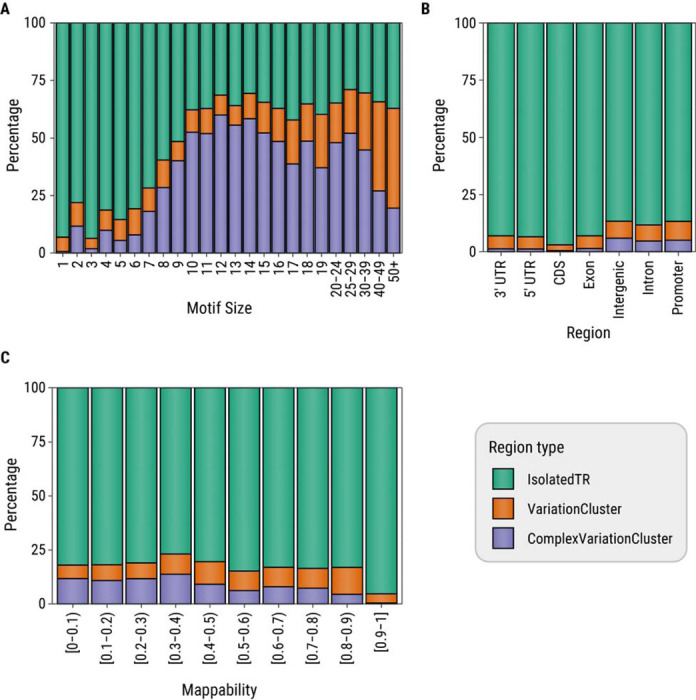
Percentage of tandem repeat (TR) loci in each variation cluster (VC) status group as a function of (A) motif size (in bp), (B) Gencode genomic region, and (C) mappability of tandem repeat locus and flanks. Isolated TRs are TRs that do not lie within a VC, complex VCs are VCs that contain five or more TRs.

We investigated the distribution of features that differed between isolated TRs compared to those within VCs or complex VCs (Figure 5). The majority of TRs with 1–9 bp repeat motifs are isolated TRs (88%), with this trend being most pronounced for homopolymer and trinucleotide TRs (93% and 94%, respectively). In contrast, only 34% of TRs with motifs 10 bp or larger are isolated TRs, while nearly half (46%) are located within complex VCs (Figure 5A). We also observed a higher proportion of isolated TRs in coding or untranslated regions (94%) relative to intronic, promoter, or intergenic regions (88%). Conversely, a higher percentage of TRs in intronic, promoter, or intergenic regions lie within complex VCs (5%) compared to those in coding or untranslated regions (1%) (Figure 5B). Finally, we found that TRs within VCs tend to have lower mappability while TRs with high mappability are nearly all isolated TRs (Figure 5C).

### Catalog Annotations and Utilities

To simplify the analysis of TRs in our catalog, we annotated them with several different categories of information, including their relationship to variation clusters, their gene regions based on Gencode^41^, Refseq^42^ and MANE^43^, the mappability of the TR loci and their flanking regions, and population allele frequencies derived from 78 haplotype-resolved assemblies from the HGDP and HGVSC consortia. Mappability for each TR was computed by taking the per-base scores from the 36-mer mappability track in the UCSC browser^44^ and averaging them across the repeat locus and 150bp flanks. The UCSC 36-mer mappability score for a given genomic position represents the fraction of 36bp k-mers overlapping that position that are uniquely mappable in the genome. Although 36bp is much smaller than the typical short read length, we chose this k-mer size because it provides a more sensitive estimate of mappability than larger k-mer sizes and is particularly relevant to the mappability of spanning and flanking reads that include only a small amount of flanking sequence, and that short read genotyping tools rely on to estimate repeat allele sizes. These annotations are currently available in JSON format for all loci in the catalog (Table S1). As an illustration of their utility, we used these annotations to summarize the basic properties of known disease-associated loci (Table S2) and to reproduce Figure 2 in Danzi et al. 2025^45^ which shows that many known disease-associated loci have some of the highest polymorphism rates among TR loci genome-wide (Figure S4).

Additionally, we share the *str-analysis* library of scripts and utilities for performing common operations on the catalog, such as filtering based on the above annotations, combining it with other TR catalogs, and computing various statistics (Code Availability).

## Discussion

Advances in sequencing technologies and genotyping tools may soon make TR analysis a standard part of rare and common disease discovery pipelines alongside SNVs, indels and SVs. We expect that this will involve a transition from the current variety of highly custom, specialized approaches to a small number of well-established methods and repeat catalogs. The lack of consensus around TR catalog design in particular may become increasingly problematic as population databases and other large-scale surveys of TR variation are made available. Our study is therefore an effort to build consensus by bringing issues and trade-offs involved in TR catalog design to the foreground.

While existing genome-wide TR catalogs have enabled a range of important discoveries, they are still primarily designed for specific tools and sequencing technologies that make them suboptimal choices for population resources of TR variation. Although newer catalogs^2,27,46^ cover a large fraction of repeats in the human genome, they use permissive locus definitions that include significant amounts of non-repetitive sequence. Such catalogs cannot be easily applied to studies based on short-read data which will remain important due to the wide-spread adoption and larger sample sizes of these datasets. Many older catalogs developed for short-read tools do support repeat copy number analyses in both short-read and long-read data. However, these catalogs remain incomplete, missing a substantial fraction (30% or more) of the polymorphic STR loci in our test sets.

We therefore developed a catalog that incorporates TRs identified in the reference genome as well as polymorphic TRs detected through cohort-based analyses, ensuring comprehensive representation of a wider range of TR loci that are amenable to both short-read and long-read analysis. To simplify adoption, we provide the catalog in the different formats expected by short-read and long-read tools. Additionally, we annotate the TR loci with their gene regions, population allele frequencies from 78 T2T assemblies, and other properties. Our catalog stratifies TRs into two groups: isolated TRs and variation clusters. This second group is derived using a novel tool that extends the boundaries of TRs located within broader regions of variation. This is similar to how HipSTR and LongTR dynamically extend the repeat boundaries when variation is detected close to the original repeat region. While the HipSTR/LongTR approach improves genotyping accuracy, it can lead to discordant repeat definitions when these tools are applied to different sample batches because the boundaries are adjusted at genotyping time. In contrast, defining variation cluster boundaries apriori with our new tool vclust facilitates comparisons across studies/batches in downstream analyses.

We hope that the TR catalog and the computational tools introduced in this paper will serve as a starting point for a dynamic community resource that will address the needs of most research groups involved in profiling variation in TR regions. The adoption of a shared catalog across TR studies and population databases will help avoid future challenges in the reuse and interpretation of TR analysis results, including population allele frequencies and pathogenic thresholds.

Going forward, we will extend the catalog by adding TR loci not captured by our current approach. This includes a broader set of polymorphic VNTRs and interrupted repeats. For each locus, our catalog currently specifies only the TR motifs present in the reference genome, it will be important to supplement our TR definitions with motif sets observed in other genomes.^19,47^ Future work will characterize the accuracy of TR genotypes across loci, tools, and sequencing technologies to identify loci that can be accurately genotyped using short-read tools as well as those loci that can only be resolved using long reads. The catalog would also benefit from annotations related to methylation states, interruption patterns, somatic mosaicism, and most importantly, population allele frequencies derived from larger sample sizes.

## Methods

### Catalog comparison

All catalog comparison steps were implemented in the tandem-repeat-catalogs GitHub repo compare_catalogs.py script. Prior to comparison, any compound locus definitions were split into their constituent TR loci, each of which had a single chromosome, start (0-based), end, and motif. For example, the compound definition of the *HTT* locus described by the expression (CAG)*CAACAG(CCG)* would be split into two separate definitions with the motifs CAG and CCG. For the Vamos v1.2 catalog, the first motif was selected from each motif set. The PlatinumTRs v1.0 TRGT catalog contained compound locus definitions that typically spanned multiple adjacent TRs without specifying the boundaries between them. To use the same approach for all catalogs, we attempted to split these compound definitions into their constituent TRs by locating stretches of perfect repeats of each specified motif within the reference sequence, as implemented in the str-analysis repo convert_trgt_catalog_to_expansion_hunter_catalog.py script. For 296,782 out of 7,722,729 locus definitions (3.8%), the exact boundaries of constituent TRs couldn’t be resolved in this way due to interruptions in the reference sequence, so we excluded them from comparisons and statistics.

In Table 1, comparison of each catalog with the test set of polymorphic STRs was performed by converting the catalogs to BED format, then running bedtools v2.31.0^48^ using the following command:

bedtools subtract -A -a {STR_test_set.bed} -b {catalog_i.bed} | wc -l

We then divided the number of loci found to be unique to the STR test set by the overall size of the test set (250,262).

### Identifying polymorphic TR loci in 78 T2T assemblies

To identify polymorphic TRs within haplotype-resolved T2T assemblies from 78 individuals for use as our TR test set and as the 4th source of TR loci for our TR catalog (Table 1, row #4), we employed the algorithm described in Weisburd et al.^28^ Briefly, we performed assembly-to-hg38 alignments and variant detection for each individual by running dipcall v0.3 with default parameters.^49^ Then, we filtered high-confidence insertion and deletion variants to the subset that represented tandem repeat expansions or contractions. For an insertion or deletion allele to be considered a tandem repeat variant, its non-reference sequence plus its flanking reference sequences needed to consist of 3 or more repeats of some motif while spanning at least 9bp. We then merged per-sample TRs from the 78 individuals by running the merge_loci.py script provided in the str-analysis repo. The pipelines for running dipcall and generating the merged set of TR loci are implemented in the str-truth-set-v2 repo run_dipcall_pipeline.py and run_filter_vcf.py scripts.

### Detecting All Perfect Repeats in the Reference Genome

To identify uninterrupted TR stretches in the reference genome, we located all sequences that spanned at least 9bp and consisted of at least three repeats of any 1– 1000 bp motif. We implemented this method in the colab-repeat-finder GitHub repo perfect_repeat_finder.py script, and ran it as follows:

python3 perfect_repeat_finder.py --min-repeats 3 --min-span 9

--min-motif-size 1 --max-motif-size 1000 hg38.fa

Initially, we tried using TRF v4.09.1 for this purpose by setting its mismatch and indel penalties to prohibitively high values (ie. 10^7^), and running it using the command:

trf409.macosx chr22.fa 2 10000000 10000000 80 10 2 2000 -h -ngs

However, when we compared TRF output for hg38 chr22 to that of perfect_repeat_finder.py, we found that TRF missed 1,355 (2%) out of the 67,638 perfect TRs detected by our approach. At the same time, our approach detected all perfect TRs identified by TRF. Loci missed by TRF had a variety of motifs and locus sizes, including the following examples: chr22:39,325,454–39,325,467 (GAG), chr22:22,778,396–22,778,417 (GATATA), chr22:50,530,014–50,530,023 (CGG), chr22:43,504,251–43,504,261 (TGA).

### Variation plots

A variation plot (as shown in Figure 3) is a visualization of a matrix whose columns correspond to bases in some genomic region and whose rows correspond to sequenced samples. The element (□,□), of the matrix is the fraction of alternative bases observed at position □ in the sample □. The numerator of this fraction counts deleted bases, mismatched bases, and inserted bases (an insertion is assigned the reference position of its anchor base). The denominator is given by the number of HiFi reads that span the entire region. The maximum value of the fraction is set to 1.0.

### Detection of variation clusters with vclust

To determine the variation cluster (VC) that a given TR belongs to, we implemented a method called vclust, available on GitHub (https://github.com/PacificBiosciences/vclust). The method calculates the probability that each base surrounding a given TR belongs to the VC. It then iteratively extends the boundaries of the VC upstream and downstream by one base pair at a time until the probability that each flanking base belongs to a variation cluster drops below 0.5 (Figure S3). To calculate the VC probability for a given base (Figure S3A), we consider variation in a window starting at that base and extending 150 bp away from the repeat (Figure S3B). We discretize variation for each base of the window into six categories according to the fraction of observed alternative bases (≤0.10, ≤0.25, ≤0.75, ≤1.50, ≤5.00, and >5.00) producing a 150 element *variation vector* with entries in {*0*,...,*5*}. We then calculate the probability that the first base of the variation vector window □ belongs to the variation cluster using □(□□|□) = □(□ | □□)□(□□)/(□(□ | □□)□(□□) + □(□ | □□□)□(□□□)). Here, the terms □(□ | □□) and □(□ | □□□) represent probabilities that the first base of the window belongs to a variation cluster or is outside of the variation cluster respectively. They are calculated according to Figure S3D and S3E. The terms □(□□□) and □(□□) are set to 0.58 and 0.42 respectively. The distributions in Figure S3D and S3E are defined through the following analysis: We randomly picked 50,000 TRs from our catalog, and then extracted the upstream and downstream flanking sequences of these TRs from 100 HPRC HiFi samples. The resulting sequences were aligned to the corresponding segments of the hg38 reference genome. If the base adjacent to the TR (rightmost base for the upstream flank and leftmost base for the downstream flank) was soft-clipped in more than half of the samples, we designated it as being in a variation cluster. We used the corresponding (discretized) variation windows to create the distributions depicted in Figure S3D, E. A locus was designated as a variation cluster if its boundary was extended by more than 5 bp in either direction. Overlapping variation clusters were then merged together.

### TRGT LPS genotypes

Longest pure segments (LPS)^45^ within repeat alleles were calculated using the recently developed TRGT-LPS tool (https://github.com/PacificBiosciences/trgt-lps). Briefly, TRGT-LPS finds copies of a given motif using a hidden Markov model (see TRGT paper) and then calculates the longest stretch of consecutive copies of that motif, which is allowed to have at most one imperfect motif per 10 perfect motif occurrences.

### Assembly consistency analysis

The assembly consistency analysis was performed as follows. We concatenated TR allele sequences produced by TRGT with 250 bp flanks extracted from the reference genome. We aligned the resulting sequences to the HG002 genome assembly with minimap2 2.24-r1122.^50^ The edit distance between each allele sequence and the corresponding segment of the assembly was then used as a measure of consistency.

## Supplementary Material

Supplement 1

Supplement 2

Supplement 3

## Figures and Tables

**Table 1:** Properties of commonly-used TR catalogs, including the total size, total span, and motif size ranges. The “Polymorphic STRs missed” column is based on lack of overlap with a test set of 250,262 polymorphic STRs with 3–6 bp motifs identified in 78 diverse haplotype-resolved assemblies. Overlap was defined leniently as having any TR definition that overlapped the test set STR by 1 bp or more, regardless of motif.

row	catalog	total loci	genome coverage	motif sizes	locus sizes in reference	pure repeat fraction in reference	includes chrX	includes chrY	polymorphic STRs missed
1	Known disease-associated TRs + adjacent repeats & historical candidate loci	83	<0.01%	1 – 27 bp	6 – 150 bp	94%	✓	✕	N/A
2	Illumina 174k polymorphic loci	174,286	0.15%	1 – 24 bp	4 – 532 bp	100%	✓	✕	71%
3	UCSC Simple Repeat Track	965,511	9.9%	1 – 1,991 bp	13 – 499,998 bp	83%	✓	✓	38%
4	Vamos v2.1	1,243,954	2.9%	1 – 667 bp	1 – 9,975 bp	64%	✓	✓	27%
5	GangSTR v17	1,340,266	0.74%	1 – 20 bp	10 – 612 bp	100%	✓	✓	53%
6	HipSTR	1,638,945	1.4%	1 – 6bp	9 – 132,210 bp	77%	✓	✓	30%
7	Adotto v1.2 *	2,934,805	3.9%	1 – 495 bp	4 – 49,802 bp	85%	✓	✓	13%
8	popSTR	5,401,401	2.7%	1 – 6bp	8 – 136 bp	96%	✕	✕	26%
9	Platinum TRs v1.0 **	† 7,524,815	5.1%	1 – 822 bp	4 – 9,504 bp	90%	✓	✓	45%
10	Chiu et al. 2024	18,759,399	10.7%	2 – 100 bp	2 – 185,860 bp	70%	✓	✕	4%

* For the Adotto v1.2 catalog, each of the original 1,784,804 entries was split into its constituent TRs, before calculating statistics

** The PlatinumTR v1.0 catalog was converted from TRGT catalog format, splitting any compound locus definitions into their constituent TRs (Methods).

† 296,782 loci (3.8%) were excluded because their reference sequences contained interruptions that prevented us from unambiguously splitting the region into its constituent repeats.

**Table 2:** Four source catalogs that were incorporated into our tandem repeat catalog.

priority	catalog	total loci	genome coverage	motif sizes	locus sizes in reference	pure repeat fraction in reference	includes chrX	includes chrY	loci contributed to final catalog
1	Known disease-associated TRs + adjacent repeats & historical candidate loci	83	<0.01%	1 – 27 bp	6 – 150 bp	94%	✓	✕	83
2	Illumina 174k polymorphic TRs in 1kGP	174,286	0.15%	1 – 24 bp	4 – 532 bp	100%	✓	✕	174,244
3	Perfect repeats that span ≥ 9 bp and ≥ 3 repeats in hg38	4,558,281	2.0%	1 – 833 bp	9 – 2,523 bp	100%	✓	✓	4,391,197
4	Polymorphic TRs in 78 T2T assemblies	1,937,805	1.0%	1 – 833 bp	1 – 2,499 bp	100%	✓	✓	297,517
	Tandem Repeat Catalog	4,863,041	2.1%	1 – 833 bp	1 – 2,523 bp	100%	✓	✓	

## Data Availability

The Repeat Catalog is available on the Releases page of the tandem-repeat-catalog GitHub repo (https://github.com/broadinstitute/tandem-repeat-catalog). The 100 HiFi samples from the Human Pangenome Reference Consortium are available under BioProject ID PRJNA850430 (https://www.ncbi.nlm.nih.gov/bioproject/730823) in SRA. TRGT allele calls for each of the individual samples are available from the authors upon request.

## Code Availability

- https://github.com/broadinstitute/TRExplorer TRExplorer online portal client-side and server-side code
- https://github.com/broadinstitute/variation-clusters documentation and tutorials for computing variation clusters
- https://github.com/broadinstitute/tandem-repeat-catalogs scripts for generating the new repeat catalog described in this paper
- https://github.com/broadinstitute/colab-repeat-finder tool for detecting perfect repeats in nucleotide sequences
- https://github.com/broadinstitute/str-analysis utilities for filtering, annotating, and converting TR catalogs to a variety of formats
- https://github.com/broadinstitute/str-truth-set-v2 pipelines for identifying TR variants through T2T-assembly-to-reference-genome alignment via dipcall, followed by filtering and post-processing steps^28^
- https://github.com/PacificBiosciences/vclust variation cluster analysis tool
- https://github.com/PacificBiosciences/trgt-lps TRGT companion tool that processes TRGT allele sequences to detect the longest pure segments (LPS)

## References

[1] Steely C. J., Watkins W. S., Baird L. & Jorde L. B. The mutational dynamics of short tandem repeats in large, multigenerational families. Genome Biol. 23, 253 (2022). 36510265 10.1186/s13059-022-02818-4 PMC9743774

[2] Porubsky D. A familial, telomere-to-telomere reference for human de novo mutation and recombination from a four-generation pedigree. bioRxiv (2024) doi:10.1101/2024.08.05.606142.

[3] Mitra I. Patterns of de novo tandem repeat mutations and their role in autism. Nature 589, 246–250 (2021). 33442040 10.1038/s41586-020-03078-7 PMC7810352

[4] Kristmundsdottir S. Sequence variants affecting the genome-wide rate of germline microsatellite mutations. Nat. Commun. 14, 3855 (2023). 37386006 10.1038/s41467-023-39547-6 PMC10310707

[5] Fotsing S. F. The impact of short tandem repeat variation on gene expression. Nat. Genet. 51, 1652–1659 (2019). 31676866 10.1038/s41588-019-0521-9 PMC6917484

[6] Hamanaka K. Genome-wide identification of tandem repeats associated with splicing variation across 49 tissues in humans. Genome Res. 33, 435–447 (2023). 37307504 10.1101/gr.277335.122 PMC10078293

[7] Depienne C. & Mandel J.-L. 30 years of repeat expansion disorders: What have we learned and what are the remaining challenges? Am. J. Hum. Genet. 108, 764–785 (2021). 33811808 10.1016/j.ajhg.2021.03.011 PMC8205997

[8] Hannan A. J. Tandem repeats mediating genetic plasticity in health and disease. Nat. Rev. Genet. 19, 286–298 (2018). 29398703 10.1038/nrg.2017.115

[9] Dolzhenko E. ExpansionHunter Denovo: a computational method for locating known and novel repeat expansions in short-read sequencing data. Genome Biol. 21, 102 (2020). 32345345 10.1186/s13059-020-02017-z PMC7187524

[10] Dashnow H. STRling: a k-mer counting approach that detects short tandem repeat expansions at known and novel loci. Genome Biol. 23, 257 (2022). 36517892 10.1186/s13059-022-02826-4 PMC9753380

[11] Fearnley L. G., Bennett M. F. & Bahlo M. Detection of repeat expansions in large next generation DNA and RNA sequencing data without alignment. Sci. Rep. 12, 13124 (2022). 35907931 10.1038/s41598-022-17267-z PMC9338934

[12] Dolzhenko E. ExpansionHunter: a sequence-graph-based tool to analyze variation in short tandem repeat regions. Bioinformatics 35, 4754–4756 (2019). 31134279 10.1093/bioinformatics/btz431 PMC6853681

[13] Mousavi N., Shleizer-Burko S., Yanicky R. & Gymrek M. Profiling the genome-wide landscape of tandem repeat expansions. Nucleic Acids Res. 47, e90 (2019). 31194863 10.1093/nar/gkz501 PMC6735967

[14] Ziaei Jam H. LongTR: genome-wide profiling of genetic variation at tandem repeats from long reads. Genome Biol. 25, 176 (2024). 38965568 10.1186/s13059-024-03319-2 PMC11229021

[15] Dolzhenko E. Characterization and visualization of tandem repeats at genome scale. Nat. Biotechnol. (2024) doi:10.1038/s41587-023-02057-3. PMC11921810 38168995

[16] Gymrek M., Golan D., Rosset S. & Erlich Y. lobSTR: A short tandem repeat profiler for personal genomes. Genome Res. 22, 1154–1162 (2012). 22522390 10.1101/gr.135780.111 PMC3371701

[17] Dashnow H. STRetch: detecting and discovering pathogenic short tandem repeat expansions. Genome Biol. 19, 121 (2018). 30129428 10.1186/s13059-018-1505-2 PMC6102892

[18] Tankard R. M. Detecting expansions of tandem repeats in cohorts sequenced with short-read sequencing data. Am. J. Hum. Genet. 103, 858–873 (2018). 30503517 10.1016/j.ajhg.2018.10.015 PMC6288141

[19] Ren J., Gu B. & Chaisson M. J. P. vamos: VNTR annotation using efficient motif sets. bioRxiv 2022.10.07.511371 (2022) doi:10.1101/2022.10.07.511371.

[20] Kristmundsdottir S., Eggertsson H. P., Arnadottir G. A. & Halldorsson B. V. popSTR2 enables clinical and population-scale genotyping of microsatellites. Bioinformatics 36, 2269–2271 (2020). 31804671 10.1093/bioinformatics/btz913 PMC7141861

[21] Willems T. Genome-wide profiling of heritable and de novo STR variations. Nat. Methods 14, 590–592 (2017). 28436466 10.1038/nmeth.4267 PMC5482724

[22] Kaivola K. Genome-wide structural variant analysis identifies risk loci for non-Alzheimer’s dementias. Cell Genom. 3, 100316 (2023). 10.1016/j.xgen.2023.100316 PMC10300553 37388914

[23] Tanudisastro H. A., Deveson I. W., Dashnow H. & MacArthur D. G. Sequencing and characterizing short tandem repeats in the human genome. Nat. Rev. Genet. 25, 460–475 (2024). 38366034 10.1038/s41576-024-00692-3

[24] Genovese L. M., Mosca M. M., Pellegrini M. & Geraci F. Dot2dot: accurate whole-genome tandem repeats discovery. Bioinformatics 35, 914–922 (2019). 30165507 10.1093/bioinformatics/bty747 PMC6419916

[25] Benson G. Tandem repeats finder: a program to analyze DNA sequences. Nucleic Acids Res. 27, 573–580 (1999). 9862982 10.1093/nar/27.2.573 PMC148217

[26] Docs/str_generation.md at Master · Illumina/RepeatCatalogs. (Github).

[27] Chiu R., Rajan-Babu I.-S., Friedman J. M. & Birol I. A comprehensive tandem repeat catalog of the human genome. medRxiv (2024) doi:10.1101/2024.06.19.24309173. PMC12855859 41605898

[28] Weisburd B., Tiao G. & Rehm H. L. Insights from a genome-wide truth set of tandem repeat variation. (2023) doi:10.1101/2023.05.05.539588.

[29] Hiatt L. STRchive: a dynamic resource detailing population-level and locus-specific insights at tandem repeat disease loci. medRxiv (2024) doi:10.1101/2024.05.21.24307682. PMC11938676 40140942

[30] Hoyt S. J. From telomere to telomere: The transcriptional and epigenetic state of human repeat elements. Science 376, eabk3112 (2022). 10.1126/science.abk3112 PMC9301658 35357925

[31] Ziaei Jam H. A deep population reference panel of tandem repeat variation. Nat. Commun. 14, 6711 (2023). 37872149 10.1038/s41467-023-42278-3 PMC10593948

[32] Pellerin D. A common flanking variant is associated with enhanced stability of the FGF14-SCA27B repeat locus. Nat. Genet. 56, 1366–1370 (2024). 38937606 10.1038/s41588-024-01808-5 PMC11440897

[33] Rajan-Babu I.-S., Dolzhenko E., Eberle M. A. & Friedman J. M. Sequence composition changes in short tandem repeats: heterogeneity, detection, mechanisms and clinical implications. Nat. Rev. Genet. 25, 476–499 (2024). 38467784 10.1038/s41576-024-00696-z

[34] Dominik N. Normal and pathogenic variation of RFC1 repeat expansions: implications for clinical diagnosis. Brain 146, 5060–5069 (2023). 37450567 10.1093/brain/awad240 PMC10689911

[35] Ibañez K. Whole genome sequencing for the diagnosis of neurological repeat expansion disorders in the UK: a retrospective diagnostic accuracy and prospective clinical validation study. Lancet Neurol. 21, 234–245 (2022). 35182509 10.1016/S1474-4422(21)00462-2 PMC8850201

[36] van der Sanden B. P. G. H. Systematic analysis of short tandem repeats in 38,095 exomes provides an additional diagnostic yield. Genet. Med. 23, 1569–1573 (2021). 33846582 10.1038/s41436-021-01174-1

[37] Cui Y. A genome-wide spectrum of tandem repeat expansions in 338,963 humans. Cell 187, 2336–2341.e5 (2024). 38582080 10.1016/j.cell.2024.03.004 PMC11065452

[38] Gall-Duncan T., Sato N., Yuen R. K. C. & Pearson C. E. Advancing genomic technologies and clinical awareness accelerates discovery of disease-associated tandem repeat sequences. Genome Res. 32, 1–27 (2022). 34965938 10.1101/gr.269530.120 PMC8744678

[39] Rautiainen M. Telomere-to-telomere assembly of diploid chromosomes with Verkko. Nat. Biotechnol. 41, 1474–1482 (2023). 36797493 10.1038/s41587-023-01662-6 PMC10427740

[40] Rhie A. The complete sequence of a human Y chromosome. Nature 621, 344–354 (2023). 37612512 10.1038/s41586-023-06457-y PMC10752217

[41] Frankish A. GENCODE: reference annotation for the human and mouse genomes in 2023. Nucleic Acids Res. 51, D942–D949 (2023). 36420896 10.1093/nar/gkac1071 PMC9825462

[42] O’Leary N. A. Reference sequence (RefSeq) database at NCBI: current status, taxonomic expansion, and functional annotation. Nucleic Acids Res. 44, D733–45 (2016). 26553804 10.1093/nar/gkv1189 PMC4702849

[43] Morales J. A joint NCBI and EMBL-EBI transcript set for clinical genomics and research. Nature 604, 310–315 (2022). 35388217 10.1038/s41586-022-04558-8 PMC9007741

[44] Marco-Sola S., Sammeth M., Guigó R. & Ribeca P. The GEM mapper: fast, accurate and versatile alignment by filtration. Nat. Methods 9, 1185–1188 (2012). 23103880 10.1038/nmeth.2221

[45] Danzi M. C. Detailed tandem repeat allele profiling in 1,027 long-read genomes reveals genome-wide patterns of pathogenicity. bioRxivorg (2025) doi:10.1101/2025.01.06.631535.

[46] English A. Benchmarking of small and large variants across tandem repeats. bioRxiv (2023) doi:10.1101/2023.10.29.564632. PMC11952744 38671154

[47] Gu B. & Chaisson M. J. P. TRCompDB: A reference of human tandem repeat sequence and composition variation from long-read assemblies. bioRxiv 2024.08.07.607105 (2024) doi:10.1101/2024.08.07.607105.

[48] Quinlan A. R. & Hall I. M. BEDTools: a flexible suite of utilities for comparing genomic features. Bioinformatics 26, 841–842 (2010). 20110278 10.1093/bioinformatics/btq033 PMC2832824

[49] Li H. A synthetic-diploid benchmark for accurate variant-calling evaluation. Nat. Methods 15, 595–597 (2018). 30013044 10.1038/s41592-018-0054-7 PMC6341484

[50] Li H. Minimap2: pairwise alignment for nucleotide sequences. Bioinformatics 34, 3094–3100 (2018). 29750242 10.1093/bioinformatics/bty191 PMC6137996

[51] Brais B. Short GCG expansions in the PABP2 gene cause oculopharyngeal muscular dystrophy. Nat. Genet. 18, 164–167 (1998). 9462747 10.1038/ng0298-164

[52] Smith I. C. Emerging and established biomarkers of oculopharyngeal muscular dystrophy. Neuromuscul. Disord. 33, 824–834 (2023). 37926637 10.1016/j.nmd.2023.09.010

[53] Halman A., Dolzhenko E. & Oshlack A. STRipy: A graphical application for enhanced genotyping of pathogenic short tandem repeats in sequencing data. Hum. Mutat. 43, 859–868 (2022). 35395114 10.1002/humu.24382 PMC9541159

