# Defining a tandem repeat catalog and variation clusters for genome-wide analyses

**DOI:** 10.1101/2024.10.04.615514

**Published:** 2025-12-29

**Authors:** Ben Weisburd, Egor Dolzhenko, Mark F. Bennett, Matt C. Danzi, Isaac R. L. Xu, Hope Tanudisastro, Bida Gu, Adam English, Laurel Hiatt, Tom Mokveld, Guilherme De Sena Brandine, Readman Chiu, Nehir Edibe Kurtas, Helyaneh Ziaei Jam, Harrison Brand, Indhu Shree Rajan Babu, Melanie Bahlo, Mark JP Chaisson, Stephan Züchner, Melissa Gymrek, Harriet Dashnow, Michael A. Eberle, Heidi L. Rehm

**Affiliations:** 1 Program in Medical and Population Genetics, Broad Center for Mendelian Genomics, Broad Institute of MIT and Harvard, Cambridge, MA, USA; 2 Center for Genomic Medicine, Massachusetts General Hospital, Harvard Medical School, Boston, MA, USA; 3 Pacific Biosciences, 1305 O’Brien Drive, Menlo Park, CA, USA; 4 Population Health and Immunity Division, Walter and Eliza Hall Institute of Medical Research, Parkville, Victoria, Australia; 5 Department of Medical Biology, University of Melbourne, Parkville, Victoria, Australia; 6 Epilepsy Research Centre, Department of Medicine, University of Melbourne, Austin Health, Heidelberg, Victoria, Australia; 7 Dr. John T. Macdonald Foundation Department of Human Genetics and John P. Hussman Institute for Human Genomics, University of Miami Miller School of Medicine, Miami, FL, USA; 8 Centre for Population Genomics, Garvan Institute of Medical Research, Sydney, NSW, Australia; 9 Centre for Population Genomics, Murdoch Children’s Research Institute, Melbourne, VIC, Australia; 10 Faculty of Medicine and Health, University of New South Wales, Sydney, NSW, Australia; 11 Faculty of Medicine and Health, University of Sydney, Sydney, NSW, Australia; 12 Department of Quantitative and Computational Biology, University of Southern California, Los Angeles, CA; 13 Human Genome Sequencing Center, Baylor College of Medicine, Houston, TX, USA; 14 University of Utah, Department of Human Genetics, Salt Lake City, UT; 15 Canada’s Michael Smith Genome Sciences Centre, BC Cancer, Vancouver, BC, Canada; 16 Department of Computer Science and Engineering, University of California San Diego, La Jolla, CA; 17 Department of Medical Genetics, University of British Columbia, Vancouver, BC, Canada; 18 Norris Comprehensive Cancer Center, University of Southern California, Los Angeles, CA; 19 Department of Biomedical Informatics, University of Colorado Anschutz Medical Campus, Aurora, CO

## Abstract

Tandem repeat (TR) catalogs are important components of repeat genotyping studies as they define the genomic coordinates and expected motifs of all TR loci being analyzed. In recent years, genome-wide studies have used catalogs ranging in size from fewer than 200,000 to over 7 million loci. Where these catalogs overlapped, they often disagreed on locus boundaries, hindering the comparison and reuse of results across studies. Now, with multiple groups developing public databases of TR variation in large population cohorts, there is a risk that, without sufficient consensus in the choice of locus definitions, the use of divergent repeat catalogs will lead to confusion, fragmentation, and incompatibility across future resources.

In this paper, we compare existing TR catalogs and discuss desirable features of a comprehensive genome-wide catalog. We then present a new, richly annotated catalog designed for large-scale analyses and population databases. This new catalog, which we call the TRExplorer catalog v1.0, contains 4.9 million TR loci and, unlike most catalogs, is designed to be useful for both short-read and long-read analyses. It consists of 4,803,366 STRs and 59,675 VNTRs, of which 780,607 STRs and 21,888 VNTRs are both polymorphic and entirely absent from widely-used catalogs previously developed for short-read analyses. Additionally, our catalog stratifies TRs into two groups: 1) isolated TRs suitable for repeat copy number analysis using short-read or long-read data and 2) so-called variation clusters that contain TRs within wider polymorphic regions that are best studied through sequence-level analysis. To define variation clusters, we present a novel algorithm that leverages long-read HiFi sequencing data to group repeats with surrounding polymorphisms. We show that the human genome contains at least 25,000 complex variation clusters, most of which span over 120 bp and contain five or more TRs. Resolving the sequence of entire variation clusters instead of individually genotyping constituent TRs leads to a more accurate analysis of these regions and enables us to profile variation that would have been missed otherwise. We also share the trexplorer.broadinstitute.org portal which allows anyone to search, visualize, and download the catalog along with variation clusters and annotations.

## Introduction

Tandem repeats (TRs) are nucleotide sequences composed of consecutive repetitions of a shorter motif (also called a repeat unit). Typically, TRs with motif size of 6 bp or less are called short tandem repeats (STRs) while those with longer motifs are called variable number tandem repeats (VNTRs). These sequences can be perfect repeats of a single motif - for example CAG CAG CAG CAG, or they can contain interruptions - such as CAG CAG CA**A** CAG. Over the past fifteen years, wide-ranging studies have used sequencing data to investigate many aspects of TRs including mutation rates,^1–4^ effects on gene expression,^5^ splicing,^6^ methylation,^7,8^ and disease risk.^9–11^ With the exception of several specialized methods for identifying *de novo* variation,^12–14^ most TR genotyping tools^15–24^ involved in these studies require the user to provide a TR catalog which specifies the reference coordinates and motifs of each TR locus to genotype - making the choice of catalog a key factor in the overall sensitivity of a study.^25–27^

Researchers have typically generated TR catalogs using tools like Tandem Repeats Finder (TRF)^28^ that detect repetitive sequences by scanning the reference genome. Although a reference-based approach is able to identify a wide range of TR loci, it can miss polymorphic repeats that are too short or absent from the reference genome. Furthermore, methods like TRF are highly sensitive to input parameters, with some commonly-used settings yielding approximately 1 million mostly perfect TRs,^16^ while other settings produce over 10 million TRs (Table S3) that contain many sequence interruptions.^29^ An alternative approach, which we call the cohort-driven strategy, detects TR variation directly from sequencing data^12,13,30^ or from haplotype-resolved assemblies.^29,31^ The cohort-driven strategy not only prioritizes polymorphic loci, but can also capture TRs that are not well represented in the reference genome and hence missed by the reference-based approach.

In addition to the different strategies for identifying TR loci, another important aspect of catalog design is the definition of locus boundaries. Since naturally occurring repeat sequences can contain interruptions or consist of multiple arrays of highly similar motifs, defining the starts and ends of TR loci in the reference genome is often subject to ambiguity. Yet, even small, seemingly inconsequential differences in how locus boundaries are specified within different catalogs, resources, or reference materials can lead to real issues with interpretation of TR genotypes. For example, the *PABPN1* locus has two commonly used definitions. With narrower locus boundaries, the pathogenic threshold is 8 GCG repeats, while with wider boundaries, the equivalent pathogenic threshold is 12 repeats. This creates the potential for inadvertent errors in the diagnosis of autosomal dominant oculopharyngeal muscular dystrophy (OPMD) if the genotype (number of repeats) in a given individual is interpreted without awareness of which locus definition was used during genotyping (Supplementary Information).

The selection of TR loci and the specification of their boundaries can influence the suitability of a catalog for two different kinds of downstream analyses. The first approach focuses primarily on the quantification of repeat copy numbers and using them to detect outlier expansions or perform association tests with other variables like gene expression. The second approach involves sequence-level analysis of TR regions in order to characterize motif composition, interruption patterns, and properties of flanking sequences. Most existing TR genotyping tools, especially those designed for short-read data, only report repeat copy numbers and not allele sequences.^15,16^ They are unable to resolve the full sequence-level variations of complex regions such as nested repeats and repeats surrounded by other repeats or structural variants. Additionally, these tools work best with narrow locus definitions that contain only perfect or nearly-perfect repeat sequences.^31^ Therefore, catalogs designed for repeat copy number analysis benefit from setting narrow locus boundaries, and flagging TRs within complex regions as more likely to produce erroneous genotypes. On the other hand, the latest generation of tools^17,18,23^ can report allele sequences in addition to repeat copy numbers, and so enables sequence-level analysis both for isolated TRs as well as more complex repeat regions.^32,33^ Examples of TR variation that especially benefit from sequence-level analysis include the recently identified polymorphic region associated with the stability of the FGF14 repeat,^34^ a single base pair insertion in a homopolymer located within a *MUC1* VNTR that causes autosomal dominant tubulointerstitial kidney disease (ADTKD), and TRs like the *RFC1* locus whose pathogenicity is affected by sequence composition changes.^35,36^ Such loci frequently correspond to short stretches of perfect tandem repeats punctuated by insertions, deletions, and substitutions in and around the repeat sequences.

Taken together, these observations highlight the challenges of creating genome-wide TR catalogs suitable for population studies, especially multi-center studies employing different computational tools and sequencing technologies. To help address these challenges, we introduce a new genome-wide repeat catalog. We employ both cohort-based and reference-based TR identification approaches to capture a comprehensive set of loci, including those that harbor common or rare variation. To simplify adoption, we share the catalog in the different formats used by popular TR analysis tools for short- and long-read data. Our catalog also stratifies TRs into two groups: isolated TRs where repeats are surrounded by non-polymorphic flanking regions and variation clusters that contain TRs surrounded by polymorphic sequence. Due to their complexity, variation clusters are best studied through sequence level analysis, particularly using long-read data. To perform this stratification we introduce a new method for profiling population-scale variation around TRs and other regions of the genome.

Lastly, we provide the trexplorer.broadinstitute.org portal to enable interactive online exploration of the TRs and variation clusters in our catalog. Users can view population allele frequency distributions, visualize locus definitions from different catalogs, and filter TRs by various criteria such as gene region, gene name, repeat motif, and/or polymorphism rates. The filtering functionality is a key feature as it allows users embarking on new analyses to first select the subset of loci that is most relevant to their particular study objectives, and then export this subset to the file format expected by their preferred TR genotyping tool. Additionally, a large and continuously increasing number of annotations provides context for TR loci of interest.

## Results

### Existing tandem repeat catalogs

**Figure 1.**
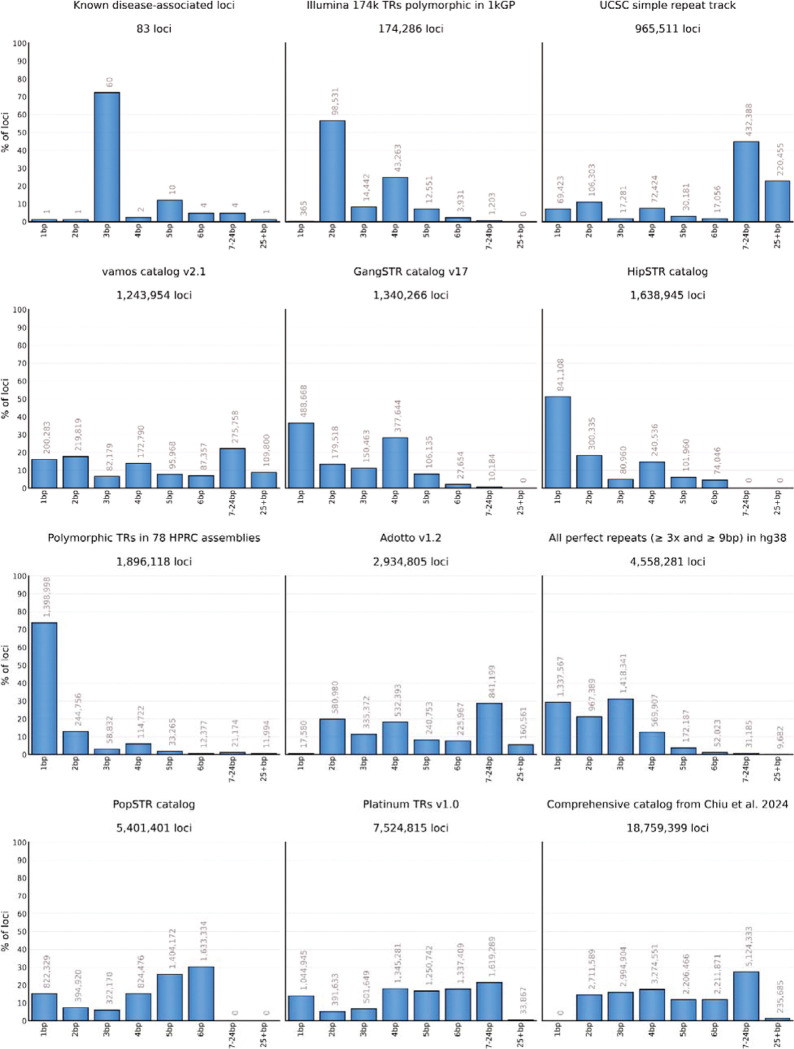
Comparison of motif size distributions across TR catalogs. The x-axis represents motif sizes. Each bar is labeled with the number of loci it contains.

Widely-used TR catalogs differ substantially in their core attributes such as their total number of loci, range of included motif sizes, repeat purity, and distribution of repeat sizes in the reference (Table 1, Figure 1, Figures S1, S2, Methods). Strikingly, pairwise comparison of eleven TR catalogs showed that every pair differed in over half of their TR definitions (Figure S1). These catalogs were generated using different analysis strategies (reference-based or cohort-driven) and aimed at different study objectives, tools and sequencing technologies. This led to different minimum repeat length and purity thresholds choices when selecting TR loci for inclusion (Table S3). Also, the repeat cataloging projects employed distinct approaches to define locus boundaries. For example, the recently-released Adotto v1.2^37^, Platinum TRs v1.0^4^, and the Chiu et al. 2024^29^ catalogs contain many long imperfect repeats and include non-repetitive flanking sequences. Such locus definitions are compatible with existing tools for genotyping TRs in long reads, but often cannot be accurately genotyped with existing tools for short-read sequencing data. On the other hand, older catalogs like GangSTR v17 (1.3 million loci) and Illumina 174k (174 thousand loci) define narrow locus boundaries around mostly perfect TR sequences and so are suitable for both short-read and long-read analyses. However, for technical or historical reasons, these early catalogs are smaller than more recent catalogs and, importantly, are missing more than half of our test set of 250,262 orthogonally-derived polymorphic STR loci with 3–6 bp motifs (Methods).

### Defining a genome-wide catalog

To incorporate the advantages of both reference-based and cohort-driven approaches, we collected loci from three cohort-driven catalogs and one reference-based catalog (Table 2). Where multiple source catalogs contained different definitions of the same TR locus (Methods), the higher prioritized definition - as described in Table 2 - was incorporated. First, we included 63 disease-associated loci, 10 of their adjacent repeats, as well as 10 candidate TR loci that have been historically included in rare disease analyses. These loci have been the focus of extensive research,^38,39^ and many have well established, widely-used locus definitions which we incorporated here to ensure compatibility with prior studies. Next, we added 174,244 loci from the Illumina catalog of polymorphic TRs which was created in 2021 by analyzing signals of TR variation within short-read sequencing data from 2,504 individuals in the 1000 Genomes Project.^30^ Prioritizing this catalog above catalogs 3 and 4 (Table 2) ensured compatibility with the locus boundary definitions used in prior studies and resources.^40^

To ensure comprehensive inclusion of perfect repeat sequences, we identified all TRs in hg38 that spanned ≥3 copies of any 3–1000 bp motif, ≥5 copies of a dinucleotide motif, or ≥9 copies of a homopolymer motif (Methods). This added another 4,391,197 loci to the catalog. Our focus on perfect repeats was motivated by their higher mutation rates compared to interrupted repeats, the importance of repeat purity in TR-associated disease mechanisms,^41,42^ as well as their higher overall genotyping accuracy in short-read analyses.^16^ We chose a ≥ 3x threshold to match several known disease-associated loci such as *C9orf72* that span only three repeats in the hg38 reference. Additionally, our initial analyses showed that this threshold represented a favorable balance between precision and recall, capturing many polymorphic TR loci in our test set without excessively inflating the size of the catalog (Figure S2 in Weisburd et al. 2023).^31^ Subsequently, we applied TRGT-LPS (Methods) to long-read samples from 1,027 individuals in the AoU phase 1 release as well as 256 individuals from the Human Pangenome Reference Consortium (HPRC) in order to directly measure the polymorphism rates at 4,023,736 out of 4,391,197 of these loci. This analysis showed that only 753 out of 4,023,736 loci (0.019%) were non-polymorphic (i.e. homozygous reference) in all individuals. However, because genotyping errors can artificially inflate the number of polymorphic loci, we also evaluated the standard deviation of the allele size distribution at each locus and found that the standard deviation exceeded 0.1 in the AoU phase 1 or the HPRC samples at 2,924,712 out of 4,023,736 (73%) loci, while at 1,446,103 (36%) loci it exceeded 0.3 which is higher than the standard deviation of some known disease-associated STR loci such as ZIC3 and PABPN1 (Table S2). We kept low polymorphism repeats in our catalog to enable studies aimed at profiling variation in populations that were not well represented in our data, to allow different studies to set their own minimum polymorphism thresholds when selecting loci for inclusion, and finally to differentiate these loci from ones that were absent from our polymorphism analyses.

Finally, we incorporated polymorphic TRs detected using 78 haplotype-resolved T2T assemblies from the Human Pangenome Reference Consortium (HPRC) and Human Genome Structural Variation Consortium (HGSVC). The computational method we employed for this task (Methods) allowed us to complement the previous three catalogs by capturing polymorphic loci that had fewer than three repeats in the reference, or that contained single base interruptions like those observed at known disease-associated loci such as *ZIC3*, *ATXN1, and FXN*. This approach contributed a further 297,519 TRs to the final catalog. It is worth noting that our catalog covers all loci in the GangSTR catalog (v17) even though we did not explicitly include it as a source catalog.

The resulting TRExplorer v1.0 catalog contained 4,863,041 TRs that collectively spanned 2.1% of the hg38 reference. As summarized in Figure S3, 4,803,366 of these (98.8%) were STRs, including 1,567,337 homopolymers (32.2%), 978,972 dinucleotide repeats (20.1%), 1,432,117 trinucleotide loci (29.4%), 590,787 loci with 4bp motifs (12.1%), 177,422 loci with 5bp motifs (3.6%), and 56,731 loci with 6bp motifs (1.2%). 59,675 were VNTRs (1.2%): 43,996 (0.9%) had 7 to 24 bp motif sizes and 15,679 (0.3%) had 25bp to 833bp motifs. Intersecting the catalog with Gencode v49 gene annotations and taking the most significant gene region based on the following priority: coding region (CDS), 5’ untranslated region (UTR), 3’ UTR, exon, intron, promoter, showed that 3,200,611 (65.8%) TRs in the catalog were intronic, 1,353,371 (27.8%) were intergenic, 49,496 (1.0%) overlapped promoters, 64,929 overlapped 3’ UTRs (1.3%), 125,242 (2.6%) overlapped exons of non-coding genes, 41,420 (0.9%) overlapped coding regions, and 27,972 (0.6%) overlapped 5’ UTRs.

To estimate the polymorphism rates of TR loci in the catalog, we used TRGT followed by TRGT-LPS (Methods) to genotype 256 HPRC HiFi samples from diverse ancestries, as well as the AoU phase 1 release data from 1,027 HiFi samples. In aggregate, 2,438,741 (52%) loci were polymorphic, having an allele size distribution standard deviation ≥ 0.2 in one or both of these datasets (Figure S7A). Stratifying polymorphism rates by motif indicated trinucleotide repeats to have the highest fraction of non-polymorphic loci (Figure S7B) which differs from other recent analyses using HiFi data like Porubsky et al. 2025 Figure 3a^4^, likely due to the inclusion of shorter loci in this study. Stratifying by source catalog (Table 2, Figure S7C) showed that the Illumina 174k catalog had the highest fraction of polymorphic loci (98%), followed by the catalog of known disease-associated loci (84.3%), followed by the catalog of polymorphic TRs in T2T assemblies (72.6%), followed by perfect repeats in hg38 that span ≥ 9bp and ≥ 3 repeats (48.4%). Downsampling analysis in individuals of African ancestry from the HPRC data showed that, after the first 50 samples, every additional sample increased the total number of loci with more than one observed allele size by 4.5k on average, while the number of loci with more than 2 observed allele sizes increased by 3.3k loci, and there was no indication that the number of polymorphic loci plateaued after 90 samples (Figure S7D).

### Analysis of variation clusters

**Figure 2:**
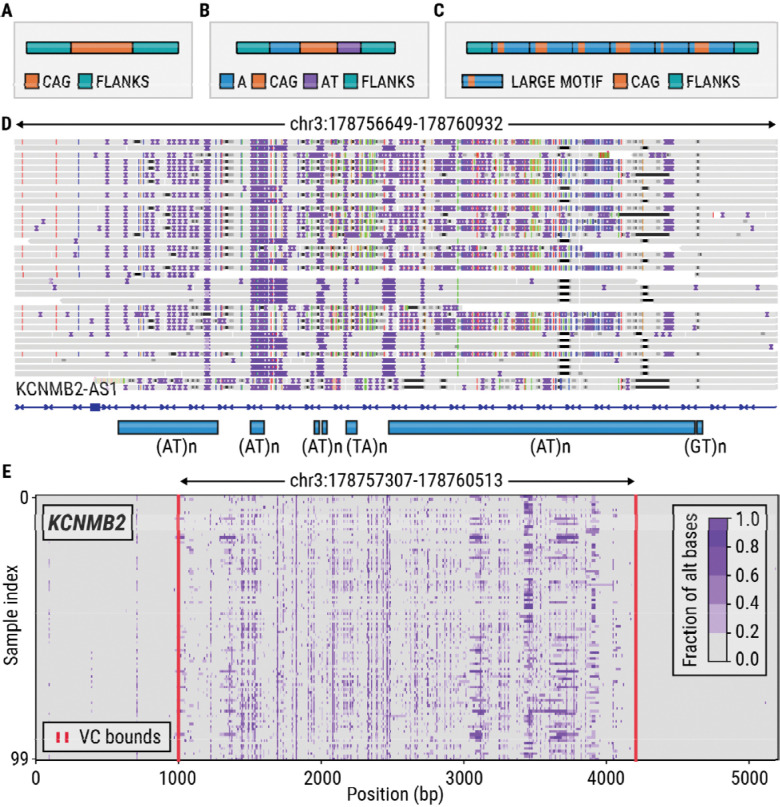
(A) A TR surrounded by non-repetitive flanks. (B) A TR with a CAG motif surrounded by an A homopolymer and AT dinucleotide repeat. (C) Nested TRs. (D) HiFi reads from the HG002 sample spanning a variation cluster located in an intron of the *KCNMB2* gene. (E) A variation plot depicting the *KCNMB2* locus in 100 long-read HiFi samples; the red vertical lines denote the boundaries of variation cluster (VC) computed by the vclust tool.

Some tandem repeats (TRs) are located within regions that have high rates of polymorphism and hence often differ significantly from the reference genome (Figure 2 A–C). We refer to such regions as variation clusters (VCs). Because most methods for TR analysis assume that the regions surrounding the TR closely resemble the reference genome, TRs located within variation clusters may be prone to analysis errors. For example, consider (AT)n repeats located in a variation cluster in the *KCNMB2* gene (Figure 2D). In the individual shown, the flanks of these TRs significantly differ from the reference genome, making it difficult to locate the exact boundaries or separate one repeat from the other repeats located within this region. This issue can be avoided if we locate the boundaries of the entire VC and then determine its full-length allele sequences in each sample with tools like TRGT^18^ or LongTR.^17^ Then the resulting sequences can be either analyzed directly or their repeat content can be characterized with existing methods,^18^ making it possible to analyze VCs together with isolated TRs.

**Figure 3.**
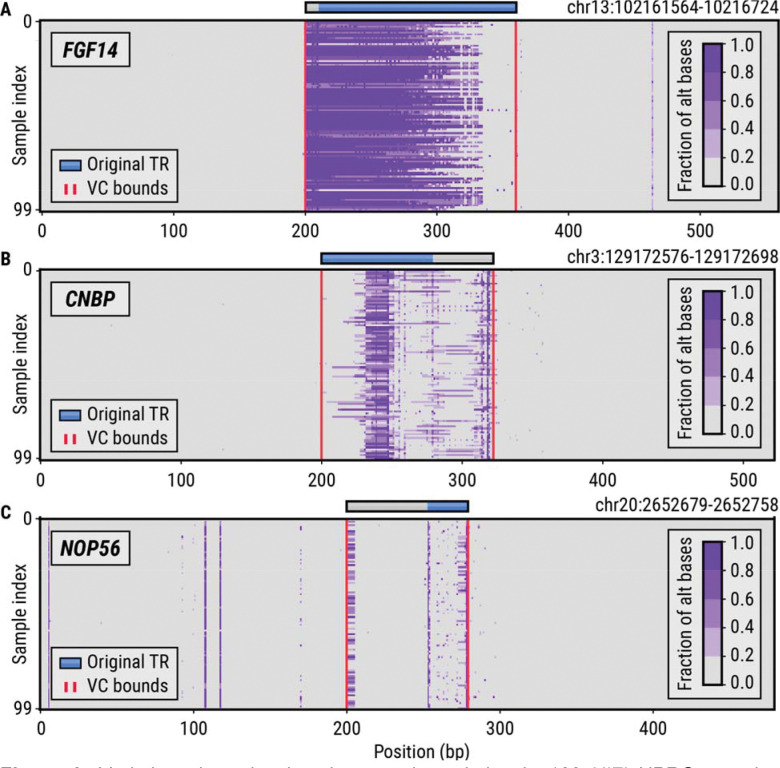
Variation plots showing the genetic variation in 100 HiFi HPRC samples around known pathogenic repeats in the (A) *FGF14*, (B) *CNBP*, and (C) *NOP56* genes. The blue horizontal bars denote the original repeat region while the gray bars depict the extension of the repeat region to a full-length variation cluster. The red vertical lines denote the boundaries of variation clusters (VCs) computed by the vclust tool.

We developed a method, vclust, that when given a set of TR regions and aligned sequencing reads from multiple samples can identify variation clusters around each TR (Methods). We first used vclust to characterize variation across 63 loci containing known disease-associated TRs in 100 HiFi WGS HPRC samples. Our method identified variation clusters around eight of these TRs located in the *ATXN8OS*, *FGF14*, *NOP56*, *CNBP*, *HTT*, *BEAN1*, *LRP12*, and *EIF4A3* genes. Variation around most of these TRs has already been characterized: the *ATXN8OS*, *NOP56*, *CNBP*, and *HTT* TRs contain known adjacent repeats that contribute to variation in their flanks. A polymorphic region flanking the *FGF14* TR was recently found to have a stabilizing effect on this repeat.^34^ As anticipated, the vclust analysis incorporated this region into the *FGF14* variation cluster (Figure 3A). The *CNBP* VC (Figure 3B) includes three polymorphic repeats: CAGG, CAGA, and CA motifs while the *NOP56* VC (Figure 3C) contains the original repeat, plus a common deletion located less than 50 bp away from the repeat boundary. *BEAN1*, *LRP12*, and *EIF4A3* VCs were caused by the presence of small indels adjacent to these repeats.

**Figure 4.**
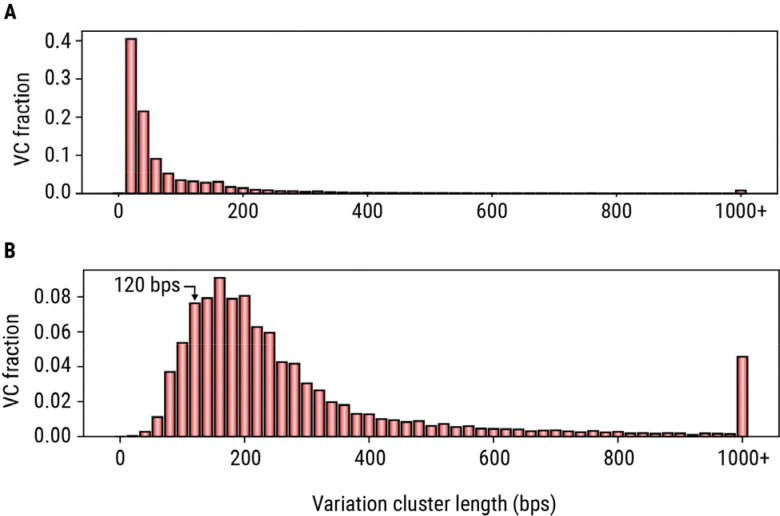
Size distributions of variation clusters. (A) Distribution of the differences between variation cluster length and the length of the original TR region contained within it. (B) Distribution of lengths of complex variation clusters in the reference genome.

Next, we applied the variation cluster analysis to our catalog of 4,863,043 TRs. This analysis identified 273,112 VCs that collectively overlapped 744,458 TRs, with 66% of VCs overlapping two or more TRs. About 18% of VCs (N=50,557) spanned more than 120 bp in the reference genome (Figure 4A), a length at which accurate genotyping with short-read data (30× 150bp WGS) becomes difficult. To assess the benefits of genotyping entire variation clusters rather than their constituent TRs, we compared the accuracy of allele sequences obtained by genotyping full VCs with those obtained by genotyping individual TRs in a long-read HiFi sequencing dataset from the HG002 sample. Accuracy was defined as sequence-level concordance with a highly accurate assembly of the same genome^43,44^ (Methods; Assembly consistency analysis). The allele concordance rate increased from 79.43% for TRs (87.30% when off-by-one repeat errors were considered concordant) to 90.58% (95.98%) for the VCs containing these TRs. In contrast, isolated TRs showed a concordance rate of 96.21% (98.50%), indicating that TRs located within VCs are a major source of genotyping errors when analyzed individually. To further characterize VCs containing multiple repeat tracts, we identified 24,867 VCs that each contained five or more constituent TRs, which we refer to as complex VCs. These complex VCs collectively contain 254,879 TRs, have a median reference length of 208 bp, and in 86% of cases exceed 120 bp (Figure 4B). For example, a VC in the *KCNMB2* gene (Figure 2E) spans over 3 Kbp in the reference genome and contains 135 TRs, including 116 TRs with AT motifs. Other similarly complex regions can benefit from VC analysis (Figure S6).

**Figure 5.**
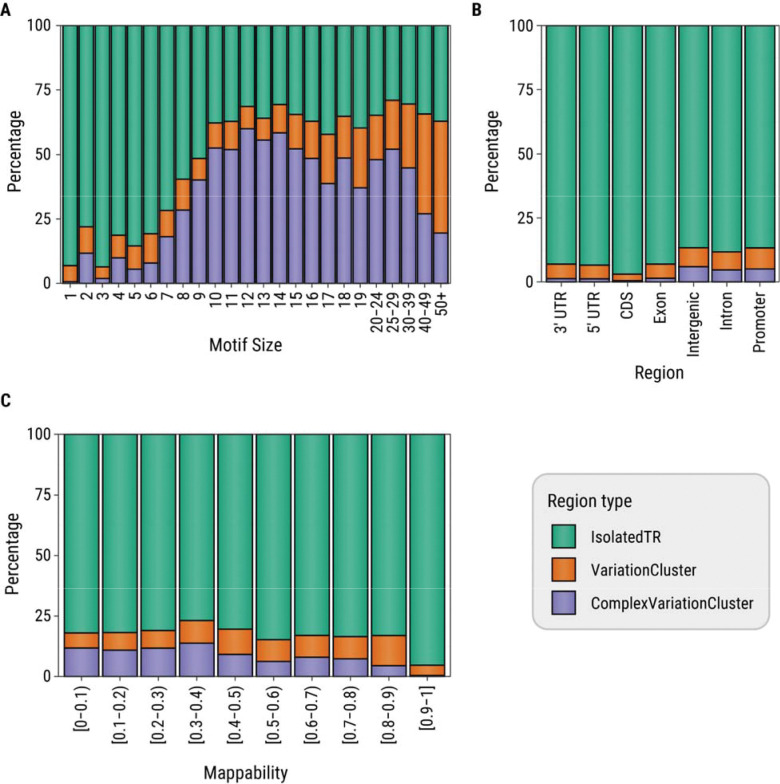
Percentage of tandem repeat (TR) loci in each variation cluster (VC) status group as a function of (A) motif size (in bp), (B) Gencode genomic region, and (C) mappability of tandem repeat locus and flanks. Isolated TRs are TRs that do not lie within a VC, complex VCs are VCs that contain five or more TRs.

We investigated the distribution of features that differed between isolated TRs compared to those within VCs or complex VCs (Figure 5). The majority of TRs with 1–9 bp repeat motifs are isolated TRs (88%), with this trend being most pronounced for homopolymer and trinucleotide TRs (93% and 94%, respectively). In contrast, only 34% of TRs with motifs 10 bp or larger are isolated TRs, while nearly half (46%) are located within complex VCs (Figure 5A). We also observed a higher proportion of isolated TRs in coding or untranslated regions (94%) relative to intronic, promoter, or intergenic regions (88%). Conversely, a higher percentage of TRs in intronic, promoter, or intergenic regions lie within complex VCs (5%) compared to those in coding or untranslated regions (1%) (Figure 5B). Finally, we found that TRs within VCs tend to have lower mappability while TRs with high mappability are nearly all isolated TRs (Figure 5C).

We also assessed whether TRs or VCs occur more frequently near other repetitive elements, such as long or short interspersed nuclear elements (LINEs or SINEs), by comparing the proportion which overlap or lie within 100 bp of the eight most frequent major classes of repetitive elements in the UCSC Genome Browser RepeatMasker annotation track^45^ (Figure S9A). We also generated a set of 1,000,000 randomly selected genomic regions with lengths sampled from the empirical distribution of TR lengths to evaluate the background rate of overlaps. As expected, TRs overlap simple repeat elements at a substantially higher frequency than random regions. SINE elements overlap isolated TRs and small VCs around twice as often as complex VCs, which are similar to the background rate (~0.2). In contrast, isolated TRs and VCs overlapped LINE elements at a rate similar to the background, whereas complex VCs were low. TRs, VCs and complex VCs exhibited higher overlap with low complexity regions than background, with the strongest enrichment for complex VCs, with over twice the proportion of overlaps (~0.1) than the next highest group. As TRs tend to cluster together, we examined whether the pattern of overlaps between TRs and repetitive elements varied depending on the number of nearby TRs (defined as the number TRs separated by no more than 6 bp of sequence). As the number of nearby TRs in the region increased, the proportion of overlaps with simple repeat elements and low complexity regions increased, while the overlap with LINEs and SINEs tended to decrease (Figure S9B).

Additionally, we performed VC downsampling analysis by picking random samples containing 1, 2, …, 10, 20, …, 90 genomes from our dataset. Then we calculated VCs for each set of samples and compared each pair of VCs by calculating the Jaccard Index (total span of the intervals belonging to both sets of VCs divided by the span of their union) between them. This analysis showed that the set of VCs becomes quite similar at the 10 sample mark (Jaccard Index > 0.8; Figure S5), suggesting that many VCs are common (note however that rare and population specific variation may occur within common VCs).

### Efficient Motif Sets

To further characterize sequence composition of TR loci in our catalog, we generated efficient motif sets for our catalog of 4,863,043 TRs at a compression level of q=0.1, following the vamos protocol.^24,46^ After filtering out loci that overlapped centromeres, had more than 500 unique motifs, or where the locus itself or its surrounding variation cluster spanned more than 10 kb, 4,734,075 loci remained. Among these, 2,138,077 loci (45%) had more than one unique motif but only 280,636 (6%) had more than one efficient motif. As shown in Figure S10, the replaced motifs were rare by counts among assemblies, indicating that efficient motifs effectively capture the primary sequence compositions in TRs.

### Catalog Annotations and Utilities

We annotated TRExplorer v1.0 catalog loci with multiple categories of information, including their rates of polymorphism in different short-read and long-read datasets, their membership in variation clusters, mappability, overlap with gene regions based on Gencode,^47^ Refseq^48^ and MANE^49^, non-coding annotations, constraint scores, RepeatMasker classes, and other data (Table S1). As an illustration of their utility, we used these annotations to summarize basic properties of known disease-associated loci (Table S2) and to reproduce Figure 2 in Danzi et al. 2025 which shows that many known disease-associated loci have some of the highest polymorphism rates among TR loci genome-wide (Figure S8). The latest set of annotations is available in JSON format on the TRExplorer portal’s Downloads page and can also be accessed in either JSON or TSV format by using the TRExplorer portal’s “export” feature above the search results table.

Additionally, we share the *str-analysis* library of scripts and utilities for performing common operations on the catalog, such as filtering based on the above annotations, combining it with other TR catalogs, and computing various statistics (Code Availability).

## Discussion

Advances in sequencing technologies and genotyping tools may soon make TR analysis a standard part of rare and common disease discovery pipelines alongside SNVs, indels and SVs. We expect that this will involve a transition from the current variety of highly custom, specialized approaches to a small number of well-established methods and repeat catalogs. The lack of consensus around TR catalog design in particular may become increasingly problematic as population databases and other large-scale surveys of TR variation are made available. Our study is therefore an effort to build consensus by bringing issues and trade-offs involved in TR catalog design to the foreground.

While existing genome-wide TR catalogs^16,23,30,33^ have enabled a range of important discoveries, they are still primarily designed for specific tools and sequencing technologies that make them suboptimal choices for population resources of TR variation. Although newer catalogs^4,29,37^ cover a large fraction of repeats in the human genome, they use permissive locus definitions that include significant amounts of non-repetitive sequence. Such catalogs cannot be easily applied to studies based on short-read data which will remain important due to the wide-spread adoption and larger sample sizes of these datasets. Many older catalogs developed for short-read tools do support repeat copy number analyses in both short-read and long-read data. However, these catalogs remain incomplete, missing a substantial fraction (30% or more) of the polymorphic STR loci in our test sets.

We therefore developed a catalog that incorporates TRs identified in the reference genome as well as polymorphic TRs detected through cohort-based analyses, ensuring comprehensive representation of a wider range of TR loci that are amenable to both short-read and long-read analysis. To simplify adoption, we provide the catalog in the different formats expected by short-read and long-read tools. Additionally, we annotate the TR loci with their gene regions, population allele frequencies, and other properties, and then make this data available for interactive exploration, visualization, filtering, and export through an online portal at trexplorer.broadinstitute.org.

Our catalog stratifies TRs into two groups: isolated TRs and variation clusters. This second group is derived using a novel tool that extends the boundaries of TRs located within broader regions of variation. This is similar to how HipSTR and LongTR dynamically extend the repeat boundaries when variation is detected close to the original repeat region. While the HipSTR/LongTR approach improves genotyping accuracy, it can lead to discordant repeat definitions when these tools are applied to different sample batches because the boundaries are adjusted at genotyping time. In contrast, defining variation cluster boundaries apriori with our new tool vclust facilitates comparisons across studies/batches in downstream analyses.

We hope that the TR catalog and the computational tools introduced in this paper will serve as a starting point for a dynamic community resource that will address the needs of most research groups involved in profiling variation in TR regions. The adoption of a shared catalog across TR studies and population databases will help avoid future challenges in the reuse and interpretation of TR analysis results, including population allele frequencies and pathogenic thresholds.

Going forward, we will extend the catalog by adding TR loci not captured by our current approach. This includes a broader set of polymorphic VNTRs and interrupted repeats. For each locus, our catalog currently specifies only the TR motifs present in the reference genome, it will be important to supplement our TR definitions with motif sets observed in other genomes.^24,46^ Future work will characterize the accuracy of TR genotypes across loci, tools, and sequencing technologies to identify loci that can be accurately genotyped using short-read tools as well as those loci that can only be resolved using long reads. The catalog would also benefit from annotations related to methylation states, interruption patterns, somatic mosaicism, and most importantly, allele frequencies derived from larger and more diverse populations, along with genotype quality scores which indicate where allele frequencies may be inaccurate.

In conclusion, repeat catalogs are a critical part of TR analysis and poorly constructed catalogs can lead to low sensitivity by missing relevant loci as well as increase the likelihood of genotyping errors through the use of locus boundaries that are suboptimal for existing tools. In this paper we discussed issues and best practices related to catalog design. Also, we proposed a comprehensive catalog that can serve as the basis for short- and long-read analyses, thereby promoting consensus across studies and population resources.

## Methods

### Catalog comparison

All catalog comparison steps were implemented in the tandem-repeat-catalogs GitHub repo compare_catalogs.py script. Prior to comparison, any compound locus definitions were split into their constituent TR loci, each of which had a single chromosome, start (0-based), end, and motif. For example, the compound definition of the *HTT* locus described by the expression (CAG)*CAACAG(CCG)* would be split into two separate definitions with the motifs CAG and CCG. For the Vamos v1.2 catalog, the first motif was selected from each motif set. The PlatinumTRs v1.0 TRGT catalog contained compound locus definitions that typically spanned multiple adjacent TRs without specifying the boundaries between them. To use the same approach for all catalogs, we attempted to split these compound definitions into their constituent TRs by locating stretches of perfect repeats of each specified motif within the reference sequence, as implemented in the str-analysis repo convert_trgt_catalog_to_expansion_hunter_catalog.py script. For 296,782 out of 7,722,729 locus definitions (3.8%), the exact boundaries of constituent TRs couldn’t be resolved in this way due to interruptions in the reference sequence, so we excluded them from comparisons and statistics.

In Table 1, comparison of each catalog with the test set of polymorphic STRs was performed by converting the catalogs to BED format, then running bedtools v2.31.0^50^ using the following command:

bedtools subtract -A -a {STR_test_set.bed} -b {catalog_i.bed} | wc -l

We then divided the number of loci found to be unique to the STR test set by the overall size of the test set (250,262).

### Identifying polymorphic TR loci in 78 T2T assemblies

To identify polymorphic TRs within haplotype-resolved T2T assemblies from 78 individuals for use as our TR test set and as the 4th source of TR loci for our TR catalog (Table 1, **row #4**), we employed the algorithm described in Weisburd et al.^31^ Briefly, we performed assembly-to-hg38 alignments and variant detection for each individual by running dipcall v0.3 with default parameters.^51^ Then, we filtered high-confidence insertion and deletion variants to the subset that represented tandem repeat expansions or contractions. For an insertion or deletion allele to be considered a tandem repeat variant, its non-reference sequence plus its flanking reference sequences needed to consist of 3 or more repeats of some motif while spanning at least 9bp. We then merged per-sample TRs from the 78 individuals by running the merge_loci.py script provided in the str-analysis repo. The pipelines for running dipcall and generating the merged set of TR loci are implemented in the str-truth-set-v2 repo run_dipcall_pipeline.py and run_filter_vcf.py scripts.

### Detecting All Perfect Repeats in the Reference Genome

To identify uninterrupted TR stretches in the reference genome, we located all sequences that spanned at least 9bp and consisted of at least three repeats of any 1–1000 bp motif. We implemented this method in the colab-repeat-finder GitHub repo perfect_repeat_finder.py script, and ran it as follows:

python3 perfect_repeat_finder.py --min-repeats 3 --min-span 9 --min-motif-size 1 --max-motif-size 1000 hg38.fa

Initially, we tried using TRF v4.09.1 for this purpose by setting its mismatch and indel penalties to prohibitively high values (ie. 10^7^), and running it using the command:

trf409.macosx chr22.fa 2 10000000 10000000 80 10 2 2000 -h -ngs

However, when we compared TRF output for hg38 chr22 to that of perfect_repeat_finder.py, we found that TRF missed 1,355 (2%) out of the 67,638 perfect TRs detected by our approach. At the same time, our approach detected all perfect TRs identified by TRF. Loci missed by TRF had a variety of motifs and locus sizes, including the following examples: chr22:39,325,454–39,325,467 (GAG), chr22:22,778,396–22,778,417 (GATATA), chr22:50,530,014–50,530,023 (CGG), chr22:43,504,251–43,504,261 (TGA).

### Variation plots

A variation plot (as shown in Figure 3) is a visualization of a matrix whose columns correspond to bases in some genomic region and whose rows correspond to sequenced samples. The element ( , ) of the matrix is the fraction of alternative bases observed at position in the sample. The numerator of this fraction counts deleted bases, mismatched bases, and inserted bases (an insertion is assigned the reference position of its anchor base). The denominator is given by the number of HiFi reads that span the entire region. The maximum value of the fraction is set to 1.0. Note that the purpose of variation plots is to visually assess the presence of variation in a given region of the genome without fully resolving it. In particular, mismatches are not distinguishable from deletions and the length of the insertions cannot be ascertained. Plotting functionality within TRGT can be used for detailed visual analysis of variation in TR regions. Variation plots can be generated with the following script: https://github.com/PacificBiosciences/vclust/blob/main/utils/variation_plot.py

### Detection of variation clusters with vclust

To determine the variation cluster (VC) that a given TR belongs to, we implemented a method called vclust, available on GitHub (https://github.com/PacificBiosciences/vclust). The method calculates the probability that each base surrounding a given TR belongs to the VC. It then iteratively extends the boundaries of the VC upstream and downstream by one base pair at a time until the probability that each flanking base belongs to a variation cluster drops below 0.5 (Figure S4). To calculate the VC probability for a given base (Figure S4A), we consider variation in a window starting at that base and extending 150 bp away from the repeat (Figure S4B). We discretize variation for each base of the window into six categories according to the fraction of observed alternative bases (≤0.10, ≤0.25, ≤0.75, ≤1.50, ≤5.00, and >5.00) producing a 150 element *variation vector* with entries in {*0,…,5*}. We then calculate the probability that the first base of the variation vector window belongs to the variation cluster using ( | ) = ( | ) ( )/( | ) ( | ) + ( | ) ( )). Here, the terms ( | ) and ( | ) represent probabilities that the first base of the window belongs to a variation cluster or is outside of the variation cluster respectively. They are calculated according to Figure S4D and S4E. The distributions in Figure S4D and S4E are defined through the following analysis: We randomly picked 50,000 TRs from our catalog, and then extracted the upstream and downstream flanking sequences of these TRs from 100 HPRC^52^ HiFi samples. The resulting sequences were aligned to the corresponding segments of the hg38 reference genome. If the base adjacent to the TR (rightmost base for the upstream flank and leftmost base for the downstream flank) was soft-clipped in more than half of the samples, we designated it as being in a variation cluster. We used the corresponding (discretized) variation windows to create the distributions depicted in Figure S4D, E. The terms ( ) and ( ) are set to 0.58 and 0.42 respectively corresponding to the fraction of repeat flanks that were softclipped by at least one base in this analysis. A locus was annotated as a variation cluster if its boundary was extended by more than 5 bp in either direction. Overlapping variation clusters were then merged together.

### TRGT longest pure segment genotypes

Longest pure segments (LPS)^53^ within repeat alleles were calculated using the TRGT-LPS tool (https://github.com/PacificBiosciences/trgt-lps). Briefly, TRGT-LPS finds copies of a given motif using a hidden Markov model (see TRGT paper)^18^ and then calculates the longest stretch of consecutive copies of that motif, which is allowed to have at most one imperfect motif per 10 perfect motif occurrences.

### Assembly consistency analysis

The assembly consistency analysis was performed as follows. We concatenated TR allele sequences produced by TRGT with 250 bp flanks extracted from the reference genome. We aligned the resulting sequences to the HG002 genome assembly with minimap2 2.24-r1122.^54^ The edit distance between each allele sequence and the corresponding segment of the assembly was then used as a measure of consistency.

## Supplementary Material

Supplement 1 https://docs.google.com/spreadsheets/d/1Pt4EWyrmQXxMQodDJzg8uo7a3cw35NYEQytAVopNLj4/edit?usp=sharing
**Supplementary Table 3: Summary of the methods used to generate existing TR catalogs.** This table shows the methods and parameters used to generate existing catalogs listed in Table 1.

Supplement 2 https://docs.google.com/spreadsheets/d/1GUpy_M5qzoOcqGoMdU7NDha4iKilDgclXGE_EqyUE5o/edit?usp=sharing
**Supplementary Table 2: Summary of annotations for known disease-associated loci.** This table lists the hg38 start (0-based) and end coordinates, motifs, gene regions based on Gencode v49, polymorphism rates (computed as the standard deviation of the TRGT-LPS repeat size distribution in 256 long-read samples from the HPRC), average mappability of the locus and flanking regions, whether the locus is embedded within a larger variation cluster (and if yes, how much larger it is than the TR locus), and finally, the number of other TR loci near this locus.

Supplement 3 https://docs.google.com/spreadsheets/d/1DPGRlr-4ZEK-0uObl8RwT8uSwggWytoNisHTTJWnxS4/edit?usp=sharing
**Supplementary Table 1: Per-locus annotations provided with the catalog.** Some annotations such as GencodeGeneName are only available for a subset of TRs. The six annotation categories are: Basic locus properties derived from the catalog itself or the reference genome (green), mappability (orange), variation clusters (blue), whether this is a known disease-associated locus (red), gene annotations (yellow), locus variability and population allele frequencies (purple).

Supplement 4

## Figures and Tables

**Table 1:** Properties of commonly-used TR catalogs, including the total size, total span, and motif size ranges. The “Polymorphic STRs missed” column is based on lack of overlap with a test set of 250,262 polymorphic STRs with 3–6 bp motifs identified in 78 diverse haplotype-resolved assemblies. Overlap was defined leniently as having any TR definition that overlapped the test set STR by 1 bp or more, regardless of motif.

row	catalog	total loci	genome coverage	motif sizes	locus sizes in reference	pure repeat fraction in reference	includes chrX	includes chrY	polymorphic STRs missed
1	Known disease-associated TRs + adjacent repeats & historical candidate loci	83	<0.01%	1 – 27 bp	6 – 150 bp	94%	✓	✗	N/A
2	Illumina 174k polymorphic loci	174,286	0.15%	1 – 24 bp	4 – 532 bp	100%	✓	✗	71%
3	UCSC Simple Repeat Track	965,511	9.9%	1 – 1,991 bp	13 – 499,998 bp	83%	✓	✓	38%
4	Vamos v2.1	1,243,954	2.9%	1 – 667 bp	1 – 9,975 bp	64%	✓	✓	27%
5	GangSTR v17	1,340,266	0.74%	1 – 20 bp	10 – 612 bp	100%	✓	✓	53%
6	HipSTR	1,638,945	1.4%	1 – 6 bp	9 – 132,210 bp	77%	✓	✓	30%
7	Adotto v1.2 *	2,934,805	3.9%	1 – 495 bp	4 – 49,802 bp	85%	✓	✓	13%
8	popSTR	5,401,401	2.7%	1 – 6 bp	8 – 136 bp	96%	✗	✗	26%
9	Platinum TRs v1.0 **	† 7,524,815	5.1%	1 – 822 bp	4 – 9,504 bp	90%	✓	✓	45%
10	Chiu et al. 2024	18,759,399	10.7%	2 – 100 bp	2 – 185,860 bp	70%	✓	✗	4%

* For the Adotto v1.2 catalog, each of the original 1,784,804 entries was split into its constituent TRs, before calculating statistics

** The PlatinumTR v1.0 catalog was converted from TRGT catalog format, splitting any compound locus definitions into their constituent TRs (Methods).

† 296,782 loci (3.8%) were excluded because their reference sequences contained interruptions that prevented us from unambiguously splitting the region into its constituent repeats.

**Table 2:** Four source catalogs that were incorporated into our tandem repeat catalog.

priority	catalog	total loci	genome coverage	motif sizes	locus sizes in reference	pure repeat fraction in reference	includes chrX	includes chrY	loci contributed to final catalog
1	Known disease-associated TRs + adjacent repeats & historical candidate loci	83	<0.01%	1 – 27 bp	6 – 150 bp	94%	✓	✗	83
2	Illumina 174k polymorphic TRs in 1kGP	174,286	0.15%	1 – 24 bp	4 – 532 bp	100%	✓	✗	174,244
3	Perfect repeats that span ≥ 9 bp and ≥ 3 repeats in hg38	4,558,281	2.0%	1 – 833 bp	9 – 2,523 bp	100%	✓	✓	4,391,197
4	Polymorphic TRs in 78 T2T assemblies	1,937,805	1.0%	1 – 833 bp	1 – 2,499 bp	100%	✓	✓	297,517
	Tandem Repeat Catalog	4,863,041	2.1%	1 – 833 bp	1 – 2,523 bp	100%	✓	✓	

## Data Availability

The Repeat Catalog is available on the Releases page of the tandem-repeat-catalog GitHub repo (https://github.com/broadinstitute/trexplorer-catalog). The HiFi samples from the Human Pangenome Reference Consortium are available under BioProject ID PRJNA850430 (https://www.ncbi.nlm.nih.gov/bioproject/730823) in SRA. TRGT allele calls in VCF format are available on the Downloads page of trexplorer.broadinstitute.org

## Footnotes

Code Availability

- https://github.com/broadinstitute/TRExplorer TRExplorer online portal client-side and server-side code
- https://github.com/PacificBiosciences/vclust variation cluster analysis tool
- https://github.com/PacificBiosciences/trgt-lps TRGT companion tool that processes TRGT allele sequences to detect the longest pure segments (LPS)^53^
- https://github.com/broadinstitute/trexplorer-catalog scripts for generating the new repeat catalog described in this paper
- https://github.com/broadinstitute/colab-repeat-finder tool for detecting perfect repeats in nucleotide sequences
- https://github.com/broadinstitute/str-analysis utilities for filtering, annotating, and converting TR catalogs to a variety of formats
- https://github.com/broadinstitute/str-truth-set-v2 pipelines for identifying TR variants through T2T-assembly-to-reference-genome alignment via dipcall, followed by filtering and post-processing steps^31^

## References

[1] Steely C. J., Watkins W. S., Baird L. & Jorde L. B. The mutational dynamics of short tandem repeats in large, multigenerational families. Genome Biol. 23, 253 (2022). 36510265 10.1186/s13059-022-02818-4 PMC9743774

[2] Mitra I. Patterns of de novo tandem repeat mutations and their role in autism. Nature 589, 246–250 (2021). 33442040 10.1038/s41586-020-03078-7 PMC7810352

[3] Kristmundsdottir S. Sequence variants affecting the genome-wide rate of germline microsatellite mutations. Nat. Commun. 14, 3855 (2023). 37386006 10.1038/s41467-023-39547-6 PMC10310707

[4] Porubsky D. Human de novo mutation rates from a four-generation pedigree reference. Nature 643, 427–436 (2025). 40269156 10.1038/s41586-025-08922-2 PMC12240836

[5] Fotsing S. F. The impact of short tandem repeat variation on gene expression. Nat. Genet. 51, 1652–1659 (2019). 31676866 10.1038/s41588-019-0521-9 PMC6917484

[6] Hamanaka K. Genome-wide identification of tandem repeats associated with splicing variation across 49 tissues in humans. Genome Res. 33, 435–447 (2023). 37307504 10.1101/gr.277335.122 PMC10078293

[7] Quilez J. Polymorphic tandem repeats within gene promoters act as modifiers of gene expression and DNA methylation in humans. Nucleic Acids Res. 44, 3750–3762 (2016). 27060133 10.1093/nar/gkw219 PMC4857002

[8] Martin-Trujillo A., Garg P., Patel N., Jadhav B. & Sharp A. J. Genome-wide evaluation of the effect of short tandem repeat variation on local DNA methylation. Genome Res. 33, 184–196 (2023). 36577521 10.1101/gr.277057.122 PMC10069470

[9] Depienne C. & Mandel J.-L. 30 years of repeat expansion disorders: What have we learned and what are the remaining challenges? Am. J. Hum. Genet. 108, 764–785 (2021). 33811808 10.1016/j.ajhg.2021.03.011 PMC8205997

[10] Hannan A. J. Tandem repeats mediating genetic plasticity in health and disease. Nat. Rev. Genet. 19, 286–298 (2018). 29398703 10.1038/nrg.2017.115

[11] Manigbas C. A. A phenome-wide association study of tandem repeat variation in 168,554 individuals from the UK Biobank. Nat. Commun. 15, 10521 (2024). 39627187 10.1038/s41467-024-54678-0 PMC11614882

[12] Dolzhenko E. ExpansionHunter Denovo: a computational method for locating known and novel repeat expansions in short-read sequencing data. Genome Biol. 21, 102 (2020). 32345345 10.1186/s13059-020-02017-z PMC7187524

[13] Dashnow H. STRling: a k-mer counting approach that detects short tandem repeat expansions at known and novel loci. Genome Biol. 23, 257 (2022). 36517892 10.1186/s13059-022-02826-4 PMC9753380

[14] Fearnley L. G., Bennett M. F. & Bahlo M. Detection of repeat expansions in large next generation DNA and RNA sequencing data without alignment. Sci. Rep. 12, 13124 (2022). 35907931 10.1038/s41598-022-17267-z PMC9338934

[15] Dolzhenko E. ExpansionHunter: a sequence-graph-based tool to analyze variation in short tandem repeat regions. Bioinformatics 35, 4754–4756 (2019). 31134279 10.1093/bioinformatics/btz431 PMC6853681

[16] Mousavi N., Shleizer-Burko S., Yanicky R. & Gymrek M. Profiling the genome-wide landscape of tandem repeat expansions. Nucleic Acids Res. 47, e90 (2019). 31194863 10.1093/nar/gkz501 PMC6735967

[17] Ziaei Jam H. LongTR: genome-wide profiling of genetic variation at tandem repeats from long reads. Genome Biol. 25, 176 (2024). 38965568 10.1186/s13059-024-03319-2 PMC11229021

[18] Dolzhenko E. Characterization and visualization of tandem repeats at genome scale. Nat. Biotechnol. (2024) doi:10.1038/s41587-023-02057-3. PMC11921810 38168995

[19] Gymrek M., Golan D., Rosset S. & Erlich Y. lobSTR: A short tandem repeat profiler for personal genomes. Genome Res. 22, 1154–1162 (2012). 22522390 10.1101/gr.135780.111 PMC3371701

[20] Dashnow H. STRetch: detecting and discovering pathogenic short tandem repeat expansions. Genome Biol. 19, 121 (2018). 30129428 10.1186/s13059-018-1505-2 PMC6102892

[21] Tankard R. M. Detecting expansions of tandem repeats in cohorts sequenced with short-read sequencing data. Am. J. Hum. Genet. 103, 858–873 (2018). 30503517 10.1016/j.ajhg.2018.10.015 PMC6288141

[22] Kristmundsdottir S., Eggertsson H. P., Arnadottir G. A. & Halldorsson B. V. popSTR2 enables clinical and population-scale genotyping of microsatellites. Bioinformatics 36, 2269–2271 (2020). 31804671 10.1093/bioinformatics/btz913 PMC7141861

[23] Willems T. Genome-wide profiling of heritable and de novo STR variations. Nat. Methods 14, 590–592 (2017). 28436466 10.1038/nmeth.4267 PMC5482724

[24] Ren J., Gu B. & Chaisson M. J. P. Vamos: Variable-number tandem repeats annotation using efficient motif sets. Genome Biol. 24, 175 (2023). 37501141 10.1186/s13059-023-03010-y PMC10373352

[25] Kaivola K. Genome-wide structural variant analysis identifies risk loci for non-Alzheimer’s dementias. Cell Genom. 3, 100316 (2023). 37388914 10.1016/j.xgen.2023.100316 PMC10300553

[26] Tanudisastro H. A., Deveson I. W., Dashnow H. & MacArthur D. G. Sequencing and characterizing short tandem repeats in the human genome. Nat. Rev. Genet. 25, 460–475 (2024). 38366034 10.1038/s41576-024-00692-3

[27] Genovese L. M., Mosca M. M., Pellegrini M. & Geraci F. Dot2dot: accurate whole-genome tandem repeats discovery. Bioinformatics 35, 914–922 (2019). 30165507 10.1093/bioinformatics/bty747 PMC6419916

[28] Benson G. Tandem repeats finder: a program to analyze DNA sequences. Nucleic Acids Res. 27, 573–580 (1999). 9862982 10.1093/nar/27.2.573 PMC148217

[29] Chiu R., Rajan-Babu I.-S., Friedman J. M. & Birol I. A comprehensive tandem repeat catalog of the human genome. medRxiv (2024) doi:10.1101/2024.06.19.24309173. PMC12855859 41605898

[30] Docs/str_generation.md at Master · Illumina/RepeatCatalogs. (Github).

[31] Weisburd B., Tiao G. & Rehm H. L. Insights from a genome-wide truth set of tandem repeat variation. (2023) doi:10.1101/2023.05.05.539588.

[32] Hoyt S. J. From telomere to telomere: The transcriptional and epigenetic state of human repeat elements. Science 376, eabk3112 (2022). 35357925 10.1126/science.abk3112 PMC9301658

[33] Ziaei Jam H. A deep population reference panel of tandem repeat variation. Nat. Commun. 14, 6711 (2023). 37872149 10.1038/s41467-023-42278-3 PMC10593948

[34] Pellerin D. A common flanking variant is associated with enhanced stability of the FGF14-SCA27B repeat locus. Nat. Genet. 56, 1366–1370 (2024). 38937606 10.1038/s41588-024-01808-5 PMC11440897

[35] Rajan-Babu I.-S., Dolzhenko E., Eberle M. A. & Friedman J. M. Sequence composition changes in short tandem repeats: heterogeneity, detection, mechanisms and clinical implications. Nat. Rev. Genet. 25, 476–499 (2024). 38467784 10.1038/s41576-024-00696-z

[36] Dominik N. Normal and pathogenic variation of RFC1 repeat expansions: implications for clinical diagnosis. Brain 146, 5060–5069 (2023). 37450567 10.1093/brain/awad240 PMC10689911

[37] English A. C. Analysis and benchmarking of small and large genomic variants across tandem repeats. Nat. Biotechnol. 43, 431–442 (2025). 38671154 10.1038/s41587-024-02225-z PMC11952744

[38] Ibañez K. Whole genome sequencing for the diagnosis of neurological repeat expansion disorders in the UK: a retrospective diagnostic accuracy and prospective clinical validation study. Lancet Neurol. 21, 234–245 (2022). 35182509 10.1016/S1474-4422(21)00462-2 PMC8850201

[39] van der Sanden B. P. G. H. Systematic analysis of short tandem repeats in 38,095 exomes provides an additional diagnostic yield. Genet. Med. 23, 1569–1573 (2021). 33846582 10.1038/s41436-021-01174-1

[40] Cui Y. A genome-wide spectrum of tandem repeat expansions in 338,963 humans. Cell 187, 2336–2341.e5 (2024). 38582080 10.1016/j.cell.2024.03.004 PMC11065452

[41] Gall-Duncan T., Sato N., Yuen R. K. C. & Pearson C. E. Advancing genomic technologies and clinical awareness accelerates discovery of disease-associated tandem repeat sequences. Genome Res. 32, 1–27 (2022). 34965938 10.1101/gr.269530.120 PMC8744678

[42] Matuszek Z. Base editing of trinucleotide repeats that cause Huntington’s disease and Friedreich’s ataxia reduces somatic repeat expansions in patient cells and in mice. Nat. Genet. 57, 1437–1451 (2025). 40419681 10.1038/s41588-025-02172-8 PMC12165863

[43] Rautiainen M. Telomere-to-telomere assembly of diploid chromosomes with Verkko. Nat. Biotechnol. 41, 1474–1482 (2023). 36797493 10.1038/s41587-023-01662-6 PMC10427740

[44] Rhie A. The complete sequence of a human Y chromosome. Nature 621, 344–354 (2023). 37612512 10.1038/s41586-023-06457-y PMC10752217

[45] Smit AFA, Hubley R & Green P. RepeatMasker Open-4.0. 2013–2015. http://www.repeatmasker.org.

[46] Gu B. & Chaisson M. J. P. TRCompDB: A reference of human tandem repeat sequence and composition variation from long-read assemblies. bioRxiv (2024) doi:10.1101/2024.08.07.607105.

[47] Frankish A. GENCODE: reference annotation for the human and mouse genomes in 2023. Nucleic Acids Res. 51, D942–D949 (2023). 36420896 10.1093/nar/gkac1071 PMC9825462

[48] O’Leary N. A. Reference sequence (RefSeq) database at NCBI: current status, taxonomic expansion, and functional annotation. Nucleic Acids Res. 44, D733–45 (2016). 26553804 10.1093/nar/gkv1189 PMC4702849

[49] Morales J. A joint NCBI and EMBL-EBI transcript set for clinical genomics and research. Nature 604, 310–315 (2022). 35388217 10.1038/s41586-022-04558-8 PMC9007741

[50] Quinlan A. R. & Hall I. M. BEDTools: a flexible suite of utilities for comparing genomic features. Bioinformatics 26, 841–842 (2010). 20110278 10.1093/bioinformatics/btq033 PMC2832824

[51] Li H. A synthetic-diploid benchmark for accurate variant-calling evaluation. Nat. Methods 15, 595–597 (2018). 30013044 10.1038/s41592-018-0054-7 PMC6341484

[52] Wang T. The Human Pangenome Project: a global resource to map genomic diversity. Nature 604, 437–446 (2022). 35444317 10.1038/s41586-022-04601-8 PMC9402379

[53] Danzi M. C. Detailed tandem repeat allele profiling in 1,027 long-read genomes reveals genome-wide patterns of pathogenicity. bioRxiv (2025) doi:10.1101/2025.01.06.631535.

[54] Li H. Minimap2: pairwise alignment for nucleotide sequences. Bioinformatics 34, 3094–3100 (2018). 29750242 10.1093/bioinformatics/bty191 PMC6137996

[55] Brais B. Short GCG expansions in the PABP2 gene cause oculopharyngeal muscular dystrophy. Nat. Genet. 18, 164–167 (1998). 9462747 10.1038/ng0298-164

[56] Smith I. C. Emerging and established biomarkers of oculopharyngeal muscular dystrophy. Neuromuscul. Disord. 33, 824–834 (2023). 37926637 10.1016/j.nmd.2023.09.010

[57] Halman A., Dolzhenko E. & Oshlack A. STRipy: A graphical application for enhanced genotyping of pathogenic short tandem repeats in sequencing data. Hum. Mutat. 43, 859–868 (2022). 35395114 10.1002/humu.24382 PMC9541159

