# Supplementary material for: Defining a tandem repeat catalog and variation clusters for genome-wide analyses
Source: bioRxiv. 2025 Dec 29:2024.10.04.615514. Preprint. [Version 3] doi: 10.1101/2024.10.04.615514 (PMC12632841; doi:10.1101/2024.10.04.615514)
Supplement: Supplementary file 1 [file NIHPP2024.10.04.615514v3-supplement-4.pdf]

bioRxiv preprint doi: https://doi.org/10.1101/2024.10.04.615514; this version posted December 29, 2025. The copyright holder for this preprint (which was not certified by peer review) is the author/funder, who has granted bioRxiv a license to display the preprint in perpetuity. It is made available under aCC-BY 4.0 International license.

Supplementary Information

Different definitions of the *PABPN1* locus

In the hg38 reference, the *PABPN1* locus locus begins with six GCGs (chr14:23,321,473 - 23,321,490), followed by three GCAs (chr14:23,321,491 - 23,321,499), followed by GCG·GGG·GCT·GCG. This sequence can be concisely represented as GCG[6]GCA[3]GCG[1]GGG[1]GCT[1]GCG[1]. Brais et al. (1998)[55] established that *PABPN1* expansions to 8 or more GCG repeats cause autosomal dominant oculopharyngeal muscular dystrophy (OPMD). Their study did not include the adjacent GCA[3]GCG[1] in the estimation of the pathogenic threshold, and thereby set locus boundaries at chr14:23,321,473 - 23,321,490. However, subsequent studies[9,56] and resources such as GeneReviews and STRchive[Citation error] extended the boundaries to chr14:23,321,473 - 23,321,499, including GCA[3]GCG[1], and therefore adjusted the pathogenic threshold to 12 or more repeats. In the meantime, other resources such as the STRipy database[57] and gnomAD continued to use Brais et al.'s original locus boundaries and pathogenic threshold of 8. While these different definitions describe the same biological reality, the inconsistency creates the potential for inadvertent errors in OPMD diagnosis.

bioRxiv preprint doi: https://doi.org/10.1101/2024.10.04.615514; this version posted December 29, 2025. The copyright holder for this preprint (which was not certified by peer review) is the author/funder, who has granted bioRxiv a license to display the preprint in perpetuity. It is made available under aCC-BY 4.0 International license.

Supplementary Figures

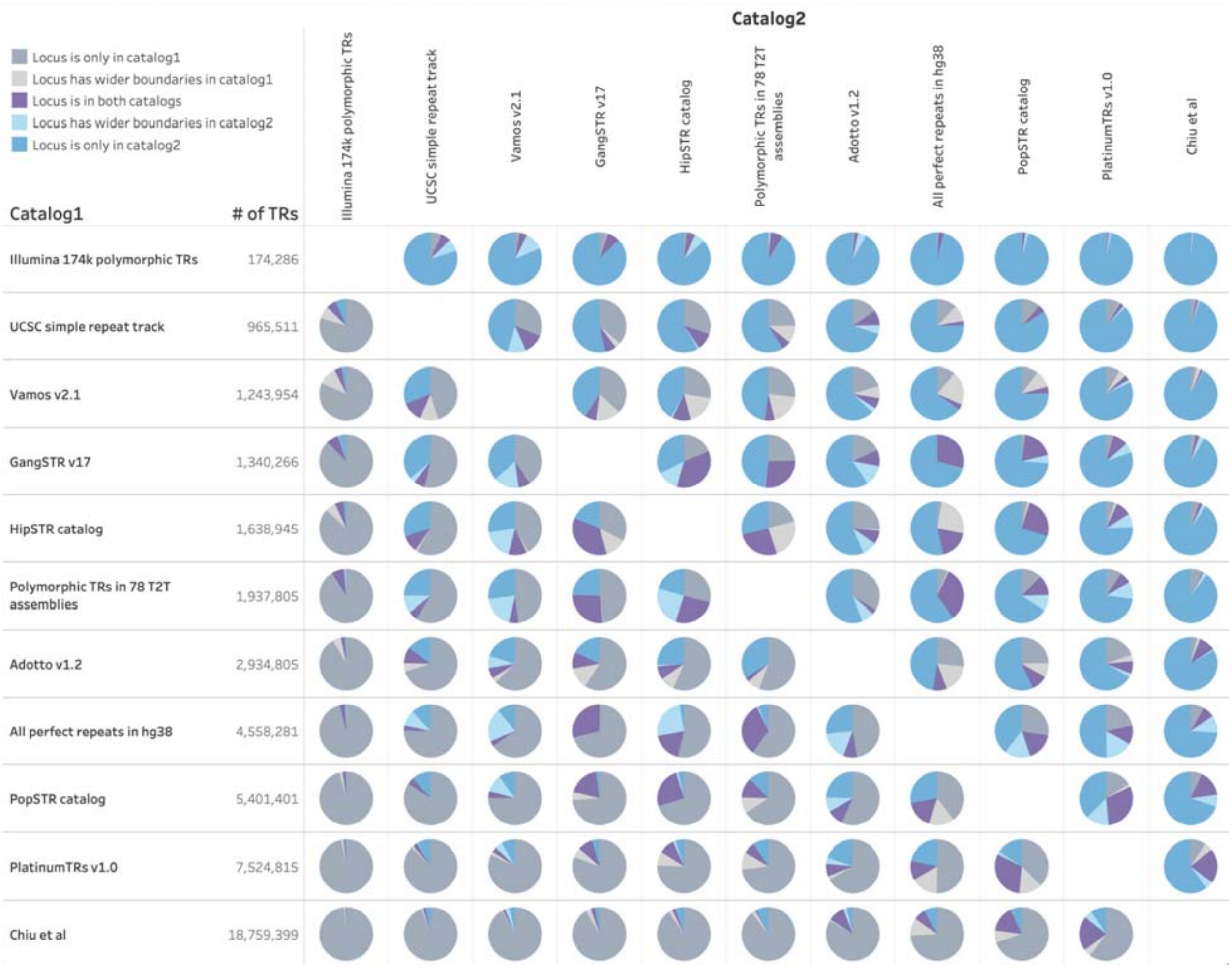

Supplementary Figure 1: Pairwise comparison of TR definitions in 11 catalogs. A locus was considered to be in both catalogs (purple) if the two locus definitions overlapped each other by at least 5%, specified the same motif, and differed in size by less than one motif length. Motif cyclic shifts and reverse complements (ie. CAG, AGC, GAC, CTG, TGC, GCT) were considered to be equivalent. Locus definitions with the same motif but wider boundaries in catalog 1 (light gray) or wider boundaries in catalog 2 (light blue) were counted separately.

bioRxiv preprint doi: https://doi.org/10.1101/2024.10.04.615514; this version posted December 29, 2025. The copyright holder for this preprint (which was not certified by peer review) is the author/funder, who has granted bioRxiv a license to display the preprint in perpetuity. It is made available under aCC-BY 4.0 International license.

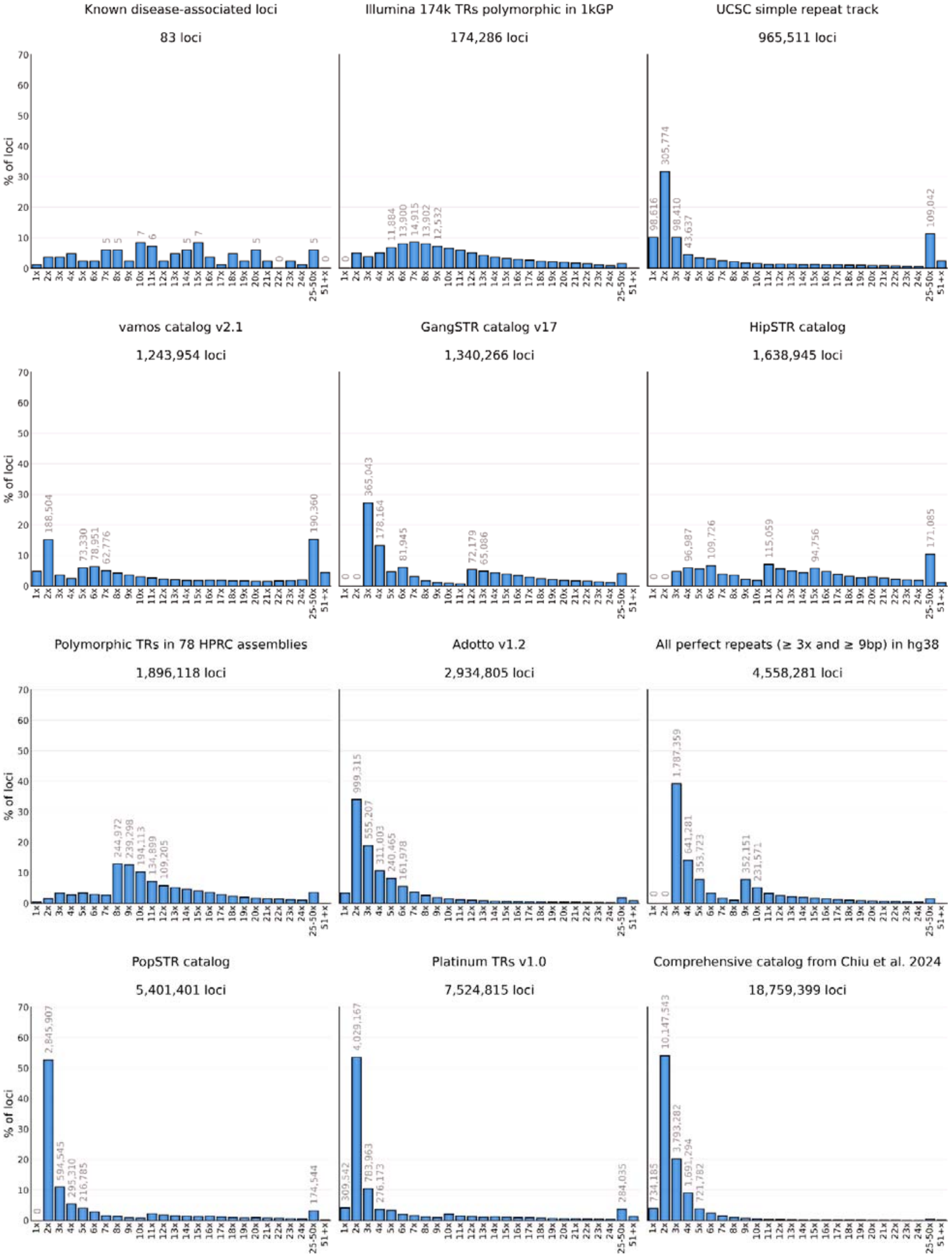

bioRxiv preprint doi: https://doi.org/10.1101/2024.10.04.615514; this version posted December 29, 2025. The copyright holder for this preprint (which was not certified by peer review) is the author/funder, who has granted bioRxiv a license to display the preprint in perpetuity. It is made available under aCC-BY 4.0 International license.

Supplementary Figure 2: Reference locus size distributions in 12 TR catalogs. The horizontal axis represents the repeat copy number of each locus in the hg38 reference.

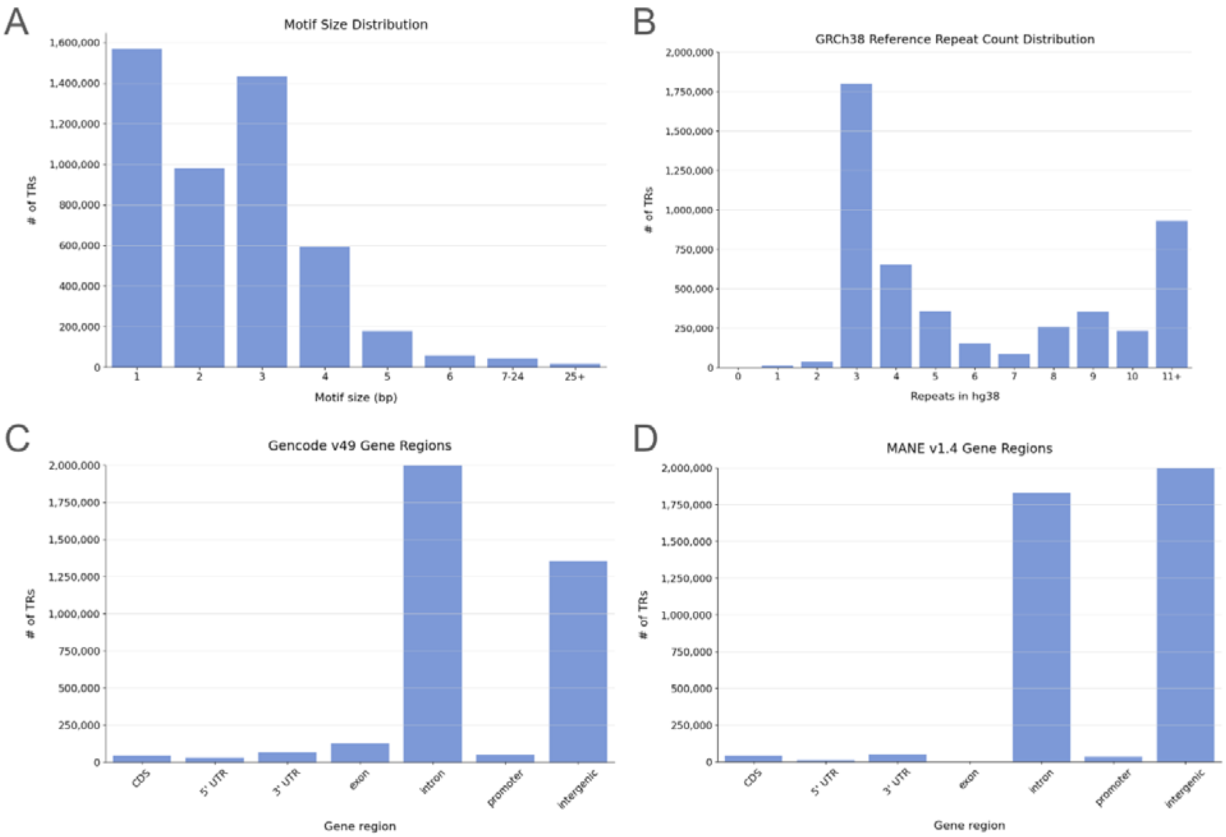

Supplementary Figure 3: TRExplorer v1.0 catalog summary statistics. (A) Motif size distribution (B) Repeat copy number in the hg38 reference (C) Most significant gene region that overlaps the locus based on the Gencode v49 comprehensive annotation (D) Most significant gene region that overlaps the locus based on the MANE v1.4 annotation

bioRxiv preprint doi: https://doi.org/10.1101/2024.10.04.615514; this version posted December 29, 2025. The copyright holder for this preprint (which was not certified by peer review) is the author/funder, who has granted bioRxiv a license to display the preprint in perpetuity. It is made available under aCC-BY 4.0 International license.

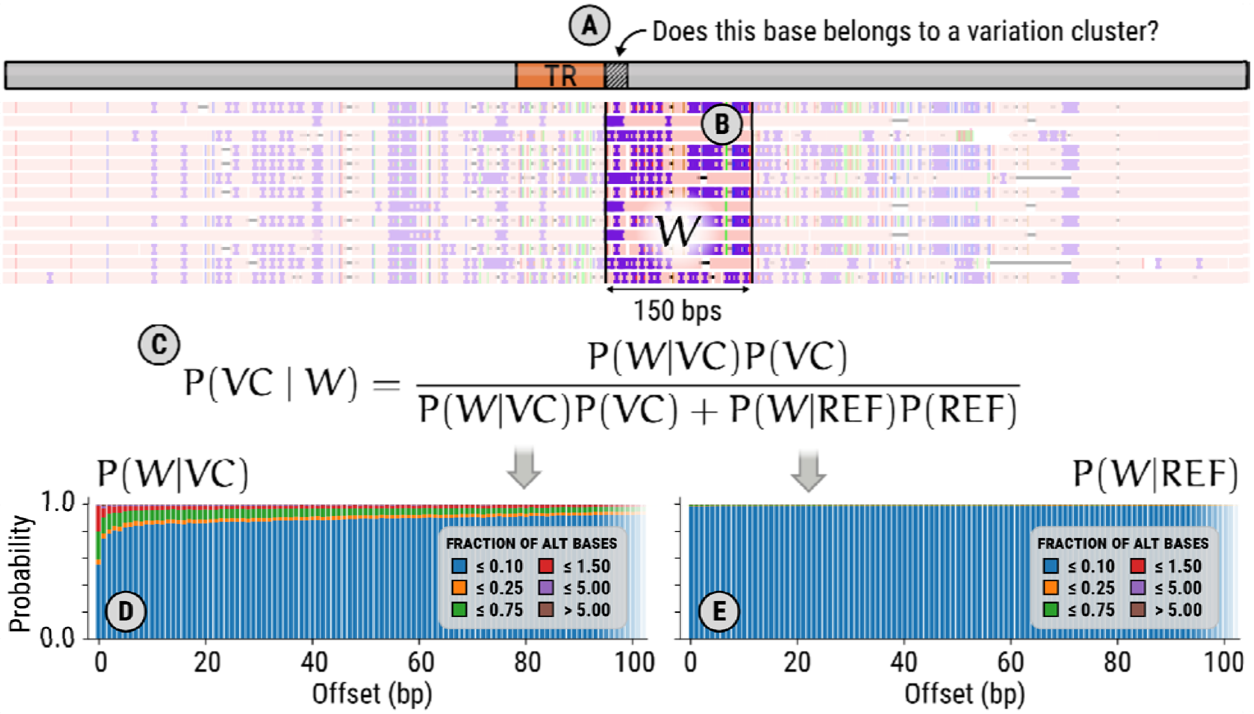

Supplementary Figure 4: Computing variation cluster boundaries. (A) The base being evaluated is adjacent to the tandem repeat. (B) The 150bp variation window that starts at the base under evaluation and extends away from the repeat. (C) The equation used to evaluate the probability that the first base of the window belongs to a variation cluster. (D) The model used to evaluate . (E) The model used to evaluate .

bioRxiv preprint doi: https://doi.org/10.1101/2024.10.04.615514; this version posted December 29, 2025. The copyright holder for this preprint (which was not certified by peer review) is the author/funder, who has granted bioRxiv a license to display the preprint in perpetuity. It is made available under aCC-BY 4.0 International license.

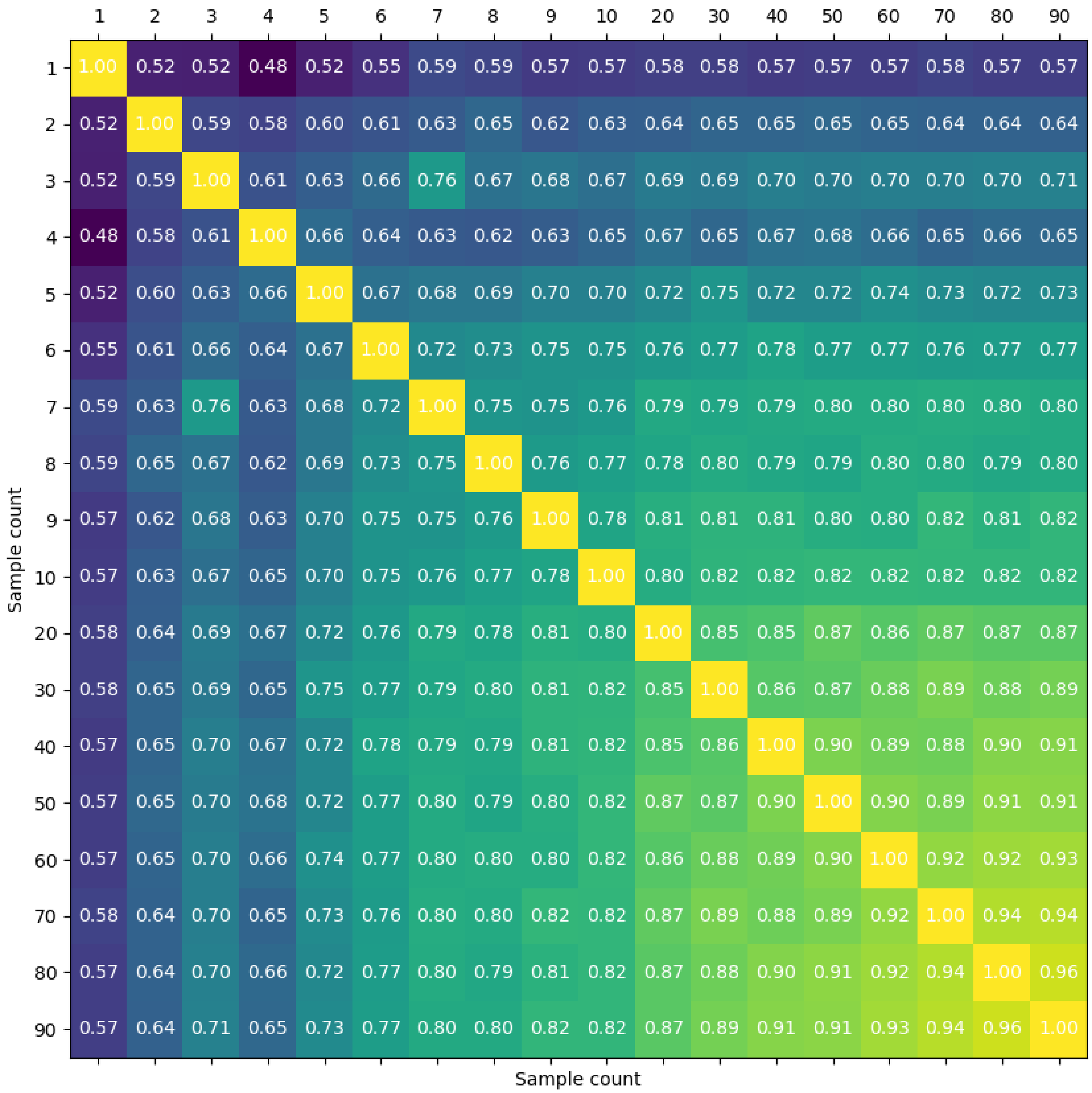

Supplementary Figure 5: Variation cluster downsampling analysis. Jaccard Index from pairwise comparisons of VCs computed from different subsets of samples. For example, the value of 0.94 at x=70 and y=90 indicates that VCs calculated using a random subset of 70 samples had an average Jaccard Index of 0.94 when compared to VCs computed using a random subset of 90 samples.

bioRxiv preprint doi: https://doi.org/10.1101/2024.10.04.615514; this version posted December 29, 2025. The copyright holder for this preprint (which was not certified by peer review) is the author/funder, who has granted bioRxiv a license to display the preprint in perpetuity. It is made available under aCC-BY 4.0 International license.

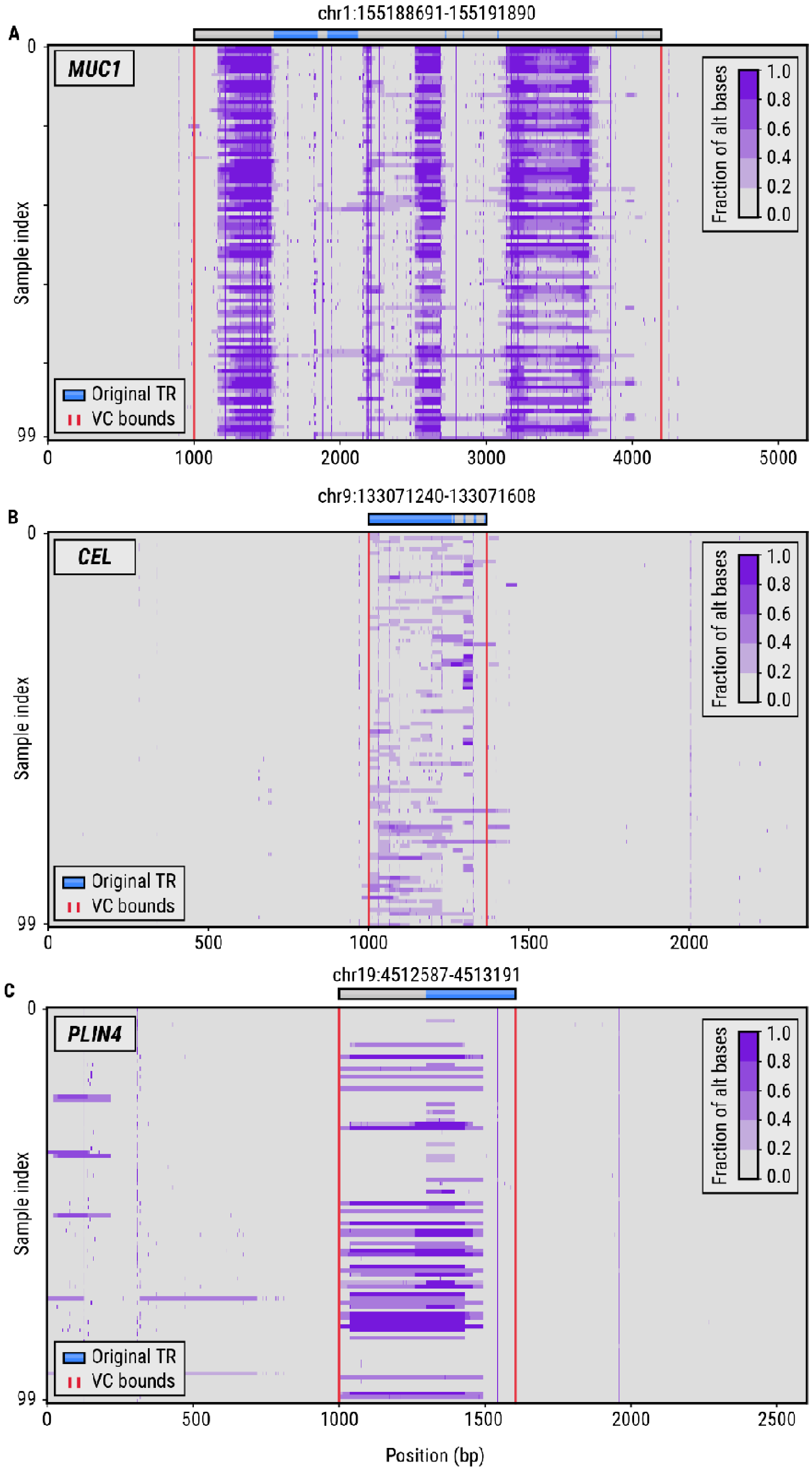

bioRxiv preprint doi: https://doi.org/10.1101/2024.10.04.615514; this version posted December 29, 2025. The copyright holder for this preprint (which was not certified by peer review) is the author/funder, who has granted bioRxiv a license to display the preprint in perpetuity. It is made available under aCC-BY 4.0 International license.

Supplementary Figure 6. Variation plots showing the genetic variation in 100 HiFi HPRC samples around VNTRs in the (A) *MUC1*, (B) *CEL*, and (C) *PLIN4* genes. The blue bars denote the boundaries of the original repeat region while the gray bars depict the extension of the repeat region to a full-length variation cluster. The red vertical lines denote the boundaries of the variation cluster (VC) computed by the vclust tool.

bioRxiv preprint doi: https://doi.org/10.1101/2024.10.04.615514; this version posted December 29, 2025. The copyright holder for this preprint (which was not certified by peer review) is the author/funder, who has granted bioRxiv a license to display the preprint in perpetuity. It is made available under aCC-BY 4.0 International license.

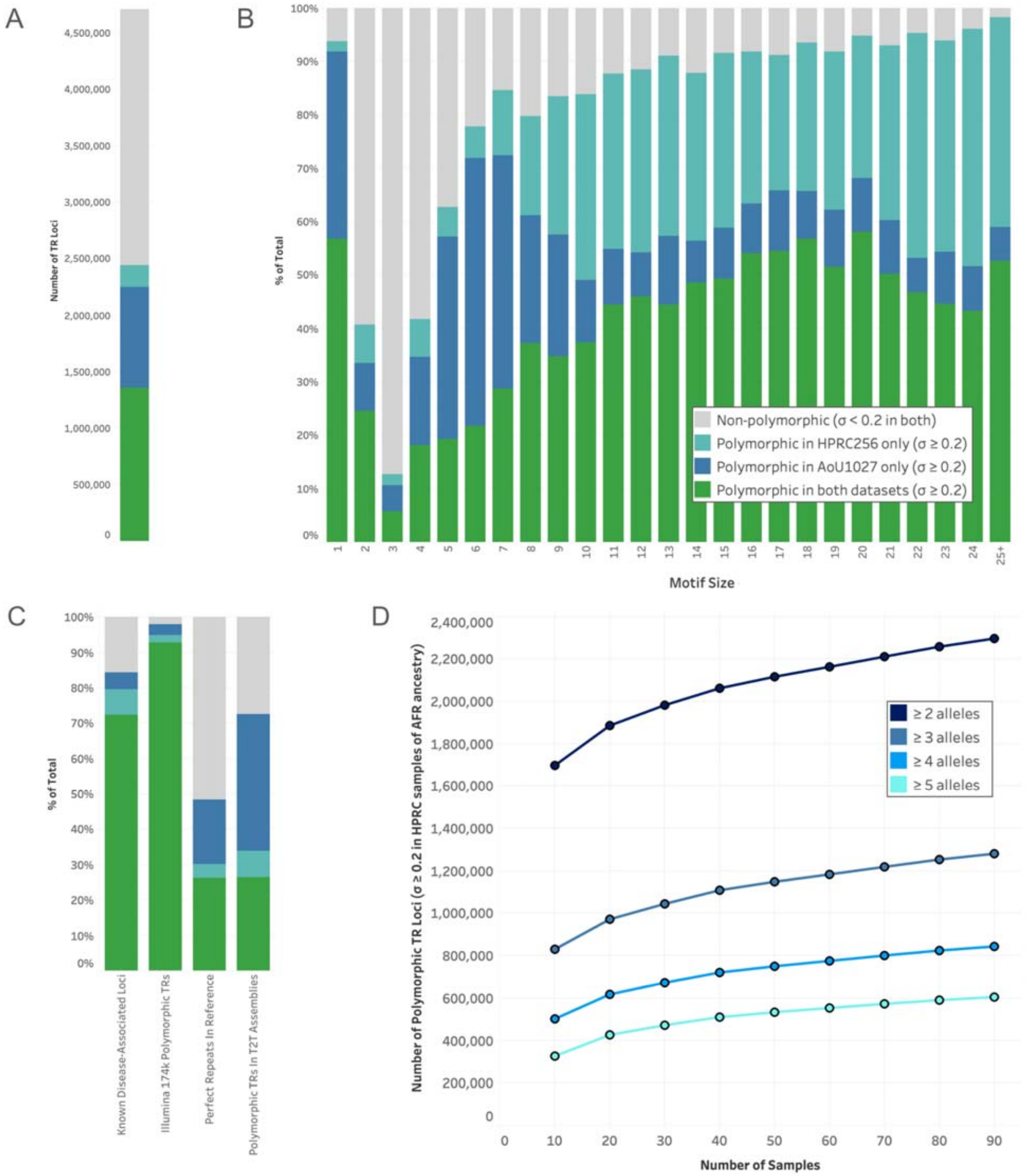

Supplementary Figure 7: Polymorphism rates. Total counts (A) and fractions (B, C) of loci that are non-polymorphic (gray) - defined as their allele size distributions having a standard deviation < 0.2 in both the AoU release 1 dataset of HiFi samples from 1,027 self-identified Black or African American individuals and in 256 HPRC HiFi samples from diverse ancestries. Fraction of loci that are polymorphic only in the HPRC samples

bioRxiv preprint doi: https://doi.org/10.1101/2024.10.04.615514; this version posted December 29, 2025. The copyright holder for this preprint (which was not certified by peer review) is the author/funder, who has granted bioRxiv a license to display the preprint in perpetuity. It is made available under aCC-BY 4.0 International license.

(teal), fraction of loci that are polymorphic only in the AoU release 1 dataset (navy blue), fraction of loci that are polymorphic in both datasets (green) stratified by motif size (B) and source catalog (C). The number of loci that have no fewer than 2, 3, 4, or 5 observed alleles by repeat count in samples of African ancestry from the HPRC dataset, downsampled to 10, 20, .. up to 90 samples. TR repeat counts in both datasets were computed by running TRGT followed by TRGT-LPS to find the number of repeats in the longest pure segment in each allele.

bioRxiv preprint doi: https://doi.org/10.1101/2024.10.04.615514; this version posted December 29, 2025. The copyright holder for this preprint (which was not certified by peer review) is the author/funder, who has granted bioRxiv a license to display the preprint in perpetuity. It is made available under aCC-BY 4.0 International license.

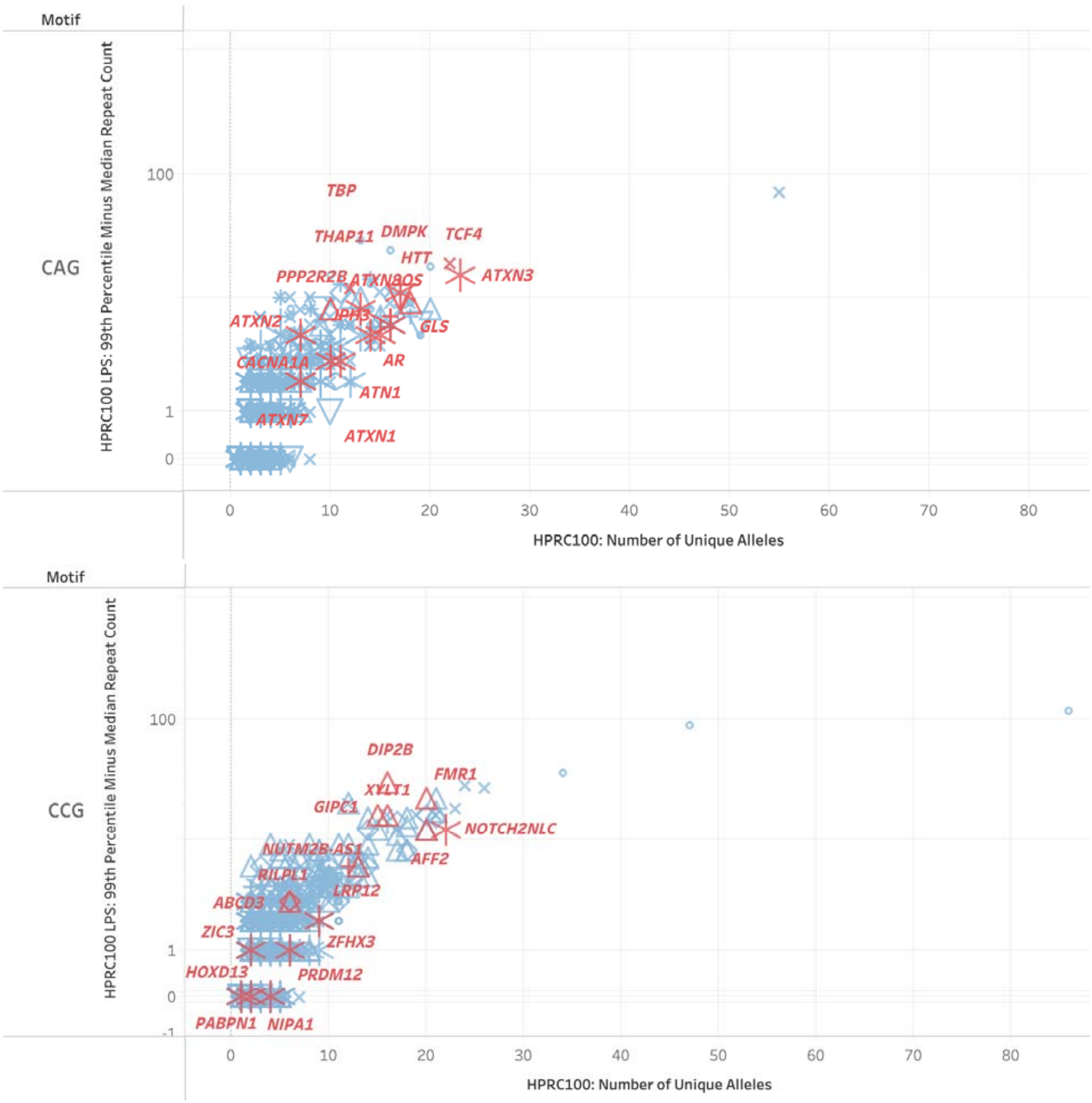

bioRxiv preprint doi: https://doi.org/10.1101/2024.10.04.615514; this version posted December 29, 2025. The copyright holder for this preprint (which was not certified by peer review) is the author/funder, who has granted bioRxiv a license to display the preprint in perpetuity. It is made available under aCC-BY 4.0 International license.

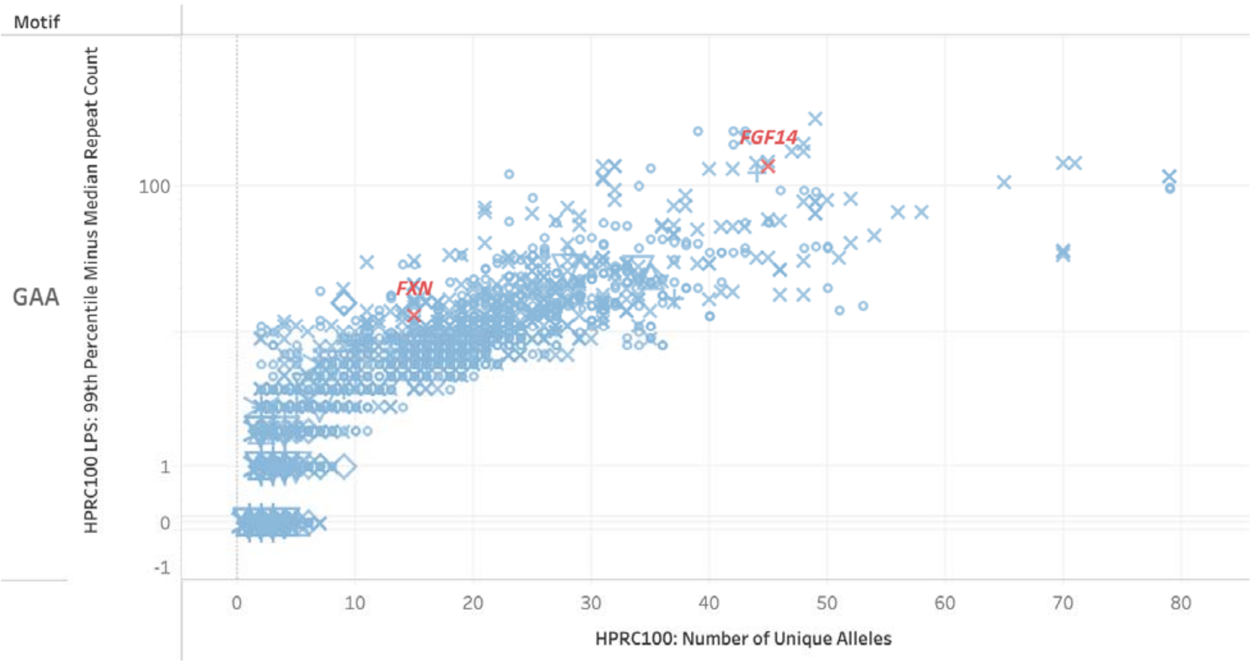

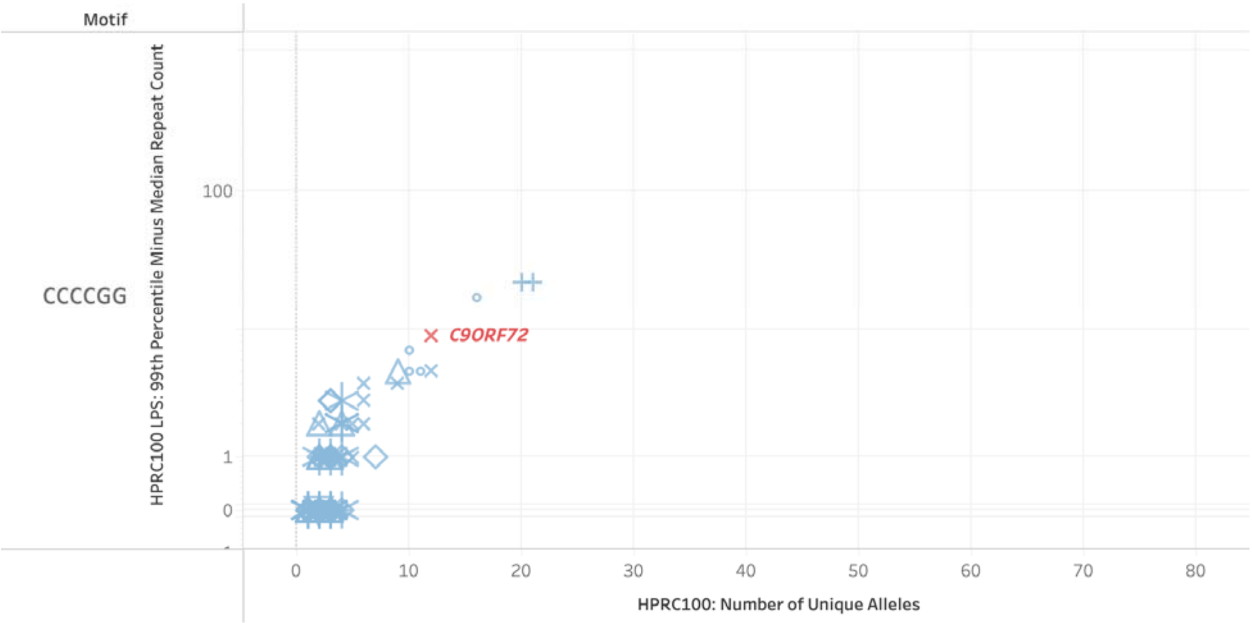

bioRxiv preprint doi: https://doi.org/10.1101/2024.10.04.615514; this version posted December 29, 2025. The copyright holder for this preprint (which was not certified by peer review) is the author/funder, who has granted bioRxiv a license to display the preprint in perpetuity. It is made available under aCC-BY 4.0 International license.

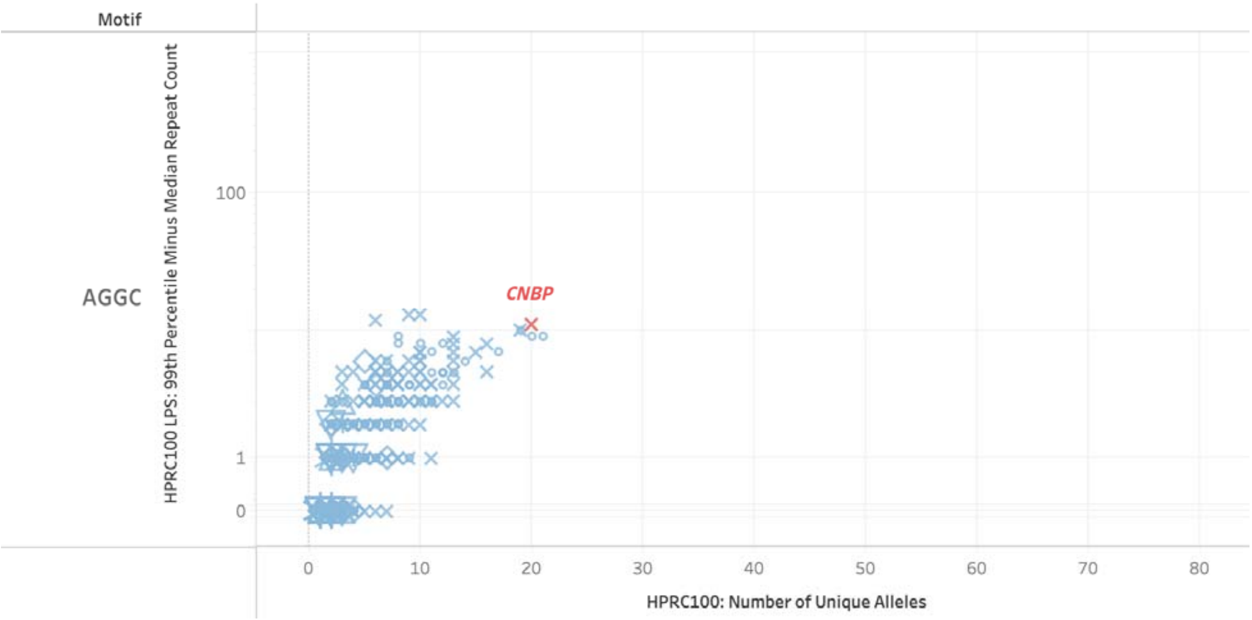

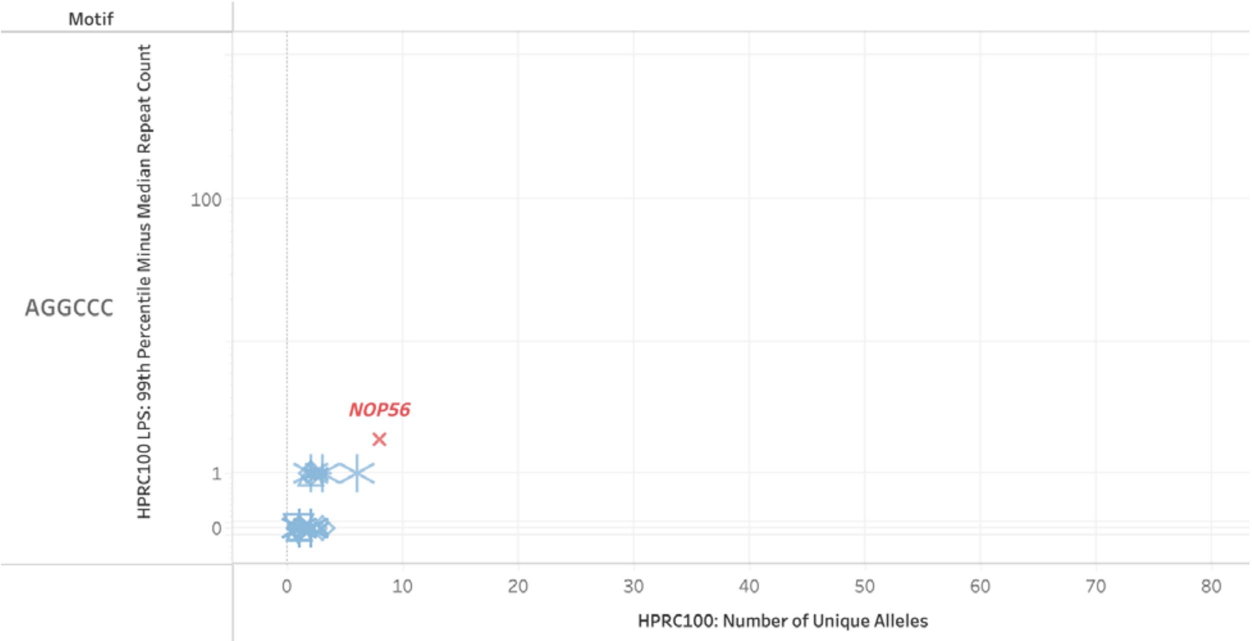

bioRxiv preprint doi: https://doi.org/10.1101/2024.10.04.615514; this version posted December 29, 2025. The copyright holder for this preprint (which was not certified by peer review) is the author/funder, who has granted bioRxiv a license to display the preprint in perpetuity. It is made available under aCC-BY 4.0 International license.

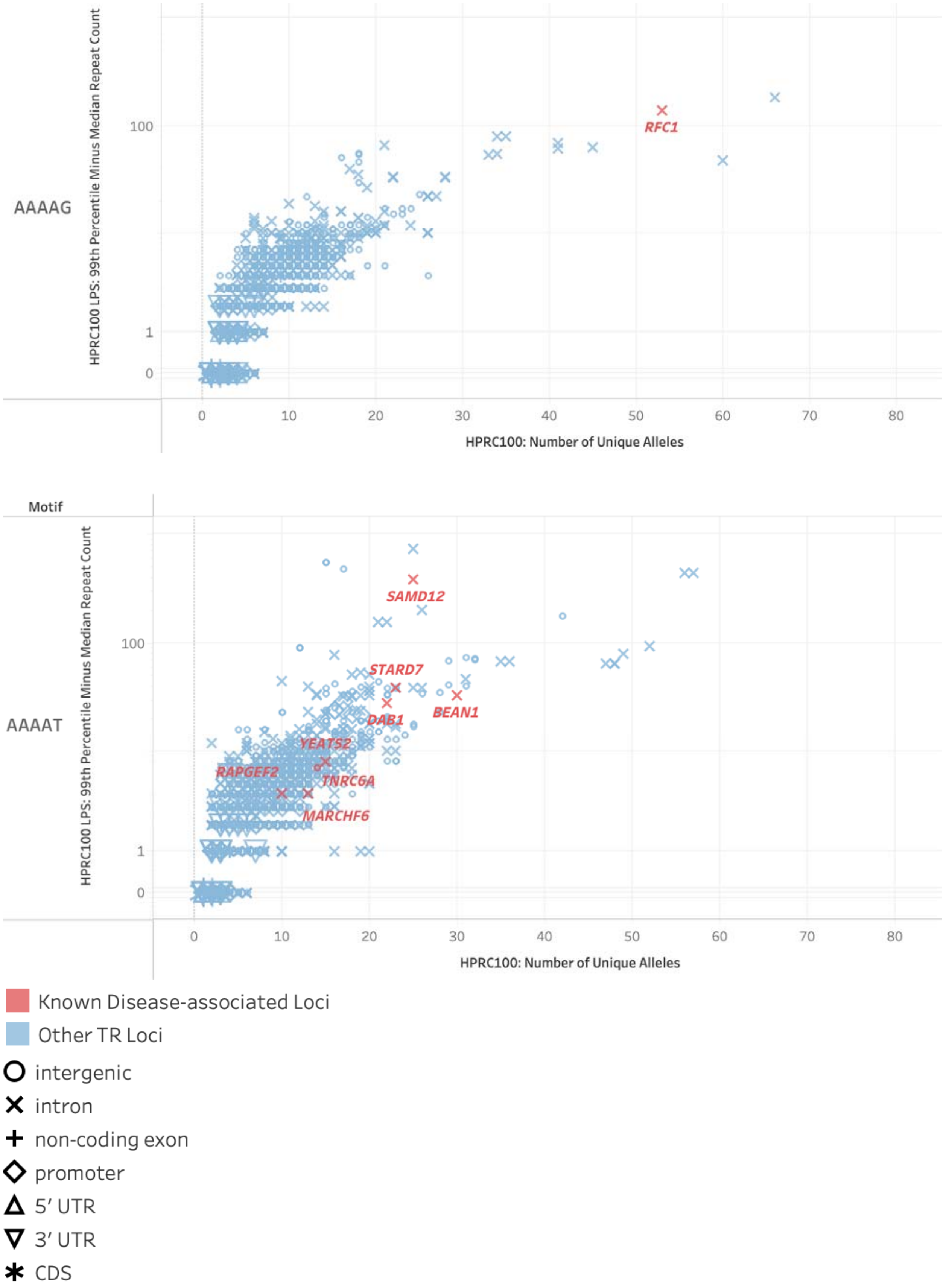

bioRxiv preprint doi: https://doi.org/10.1101/2024.10.04.615514; this version posted December 29, 2025. The copyright holder for this preprint (which was not certified by peer review) is the author/funder, who has granted bioRxiv a license to display the preprint in perpetuity. It is made available under aCC-BY 4.0 International license.

Supplementary Figure 8: Polymorphism rates stratified by motif. Comparison of known disease-associated STR loci with all other loci in the catalog that share the same normalized motif (where shifted or reverse-complemented motifs such as CAG, AGC, GCA, TGC, etc. are considered to be the same). Each panel shows data for a different STR motif: (A) CAG (B) CCG (C) GAA (D) CCCCGG (E) AGGC (F) AGGCCC (G) AAAAG (H) AAAAT. Two metrics of polymorphism are shown on the x- and y- axis: the x-axis shows the number of observed TR allele sizes at a locus, while the y-axis shows the 99th percentile allele size minus the median allele size (in units of repeat counts) at a locus. The color is red for known disease-associated loci and blue for all other loci. Different shapes represent the gene region of each locus in Gencode v46. The polymorphism metrics were generated by running TRGT-LPS with the full TR catalog on 100 long-read HPRC samples from diverse populations.

bioRxiv preprint doi: https://doi.org/10.1101/2024.10.04.615514; this version posted December 29, 2025. The copyright holder for this preprint (which was not certified by peer review) is the author/funder, who has granted bioRxiv a license to display the preprint in perpetuity. It is made available under aCC-BY 4.0 International license.

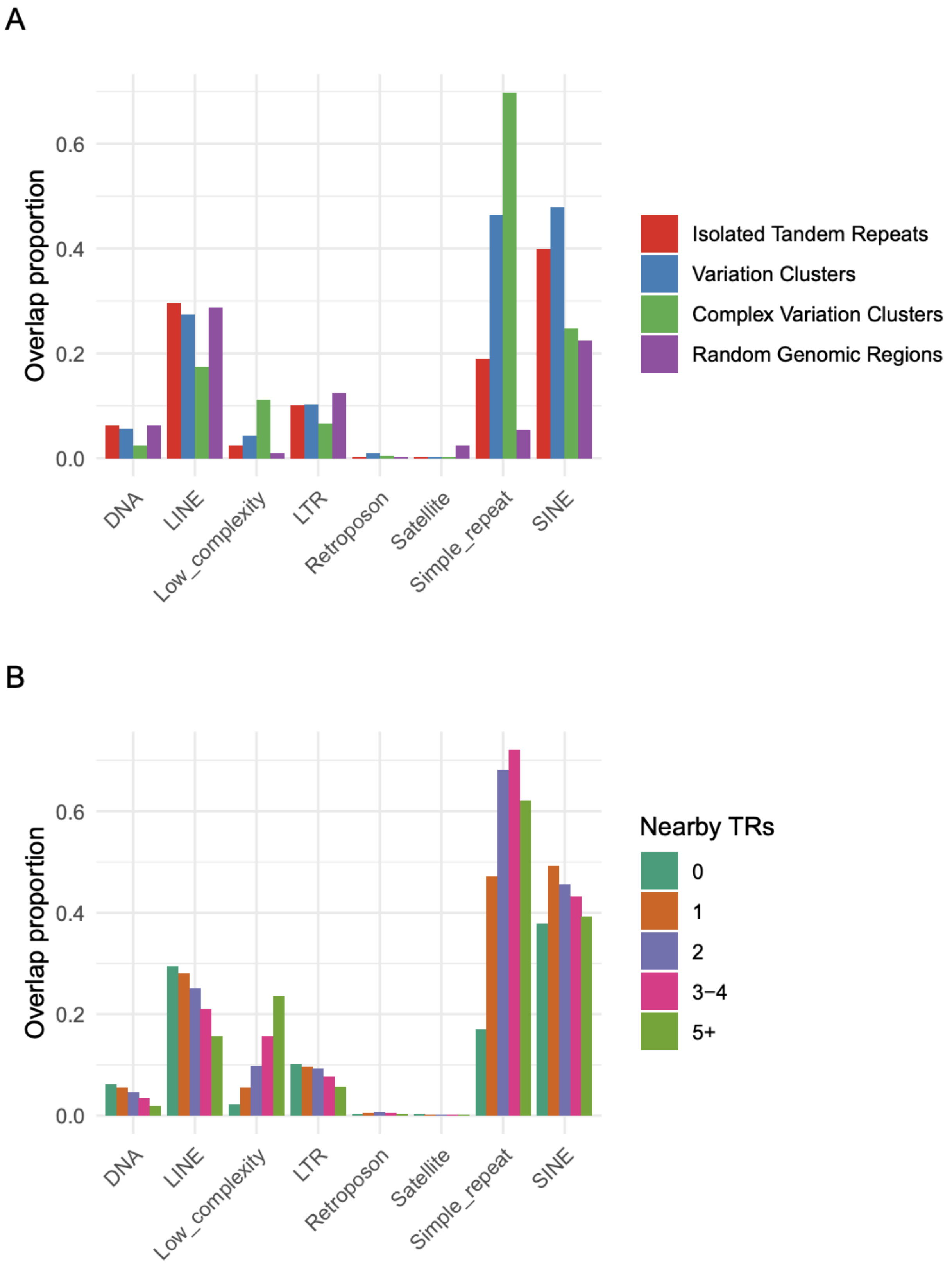

bioRxiv preprint doi: https://doi.org/10.1101/2024.10.04.615514; this version posted December 29, 2025. The copyright holder for this preprint (which was not certified by peer review) is the author/funder, who has granted bioRxiv a license to display the preprint in perpetuity. It is made available under aCC-BY 4.0 International license.

Supplementary Figure 9: Overlap with repetitive elements. (A) Proportion of TR loci that overlap or lie within 100 bp of major types of repetitive elements in UCSC Genome Browser RepeatMasker track, compared to background rate for randomly selected genomic regions. TRs that overlapped multiple RepeatMasker classes were counted under each class. (B) Proportion of TRs that overlap repetitive elements, stratified by the number of nearby TRs (defined as TRs separated by no more than 6 bp of sequence). RepeatMasker abbreviations: DNA = DNA transposon, LINE = long interspersed nuclear element, LTR = long terminal repeat, SINE = short interspersed nuclear element.

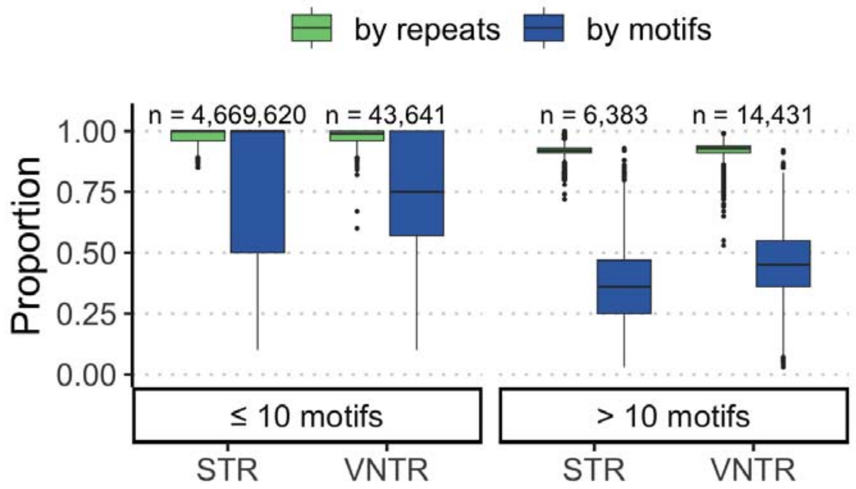

Supplementary Figure 10: Proportion of efficient motifs. TRs were grouped by the number of unique motifs observed within the assembly repeat sequences at a locus (≤ 10 or > 10). Green boxes represent the fraction of assembly repeat sequences that are composed of efficient motifs (q = 0.1) while blue boxes represent the number of efficient motifs divided by the total number of unique motifs at a locus.
