# Supplementary material for: Defining a tandem repeat catalog and variation clusters for genome-wide analyses
Source: bioRxiv. 2025 Oct 16:2024.10.04.615514. Preprint. [Version 2] doi: 10.1101/2024.10.04.615514 (PMC12632841; doi:10.1101/2024.10.04.615514)
Supplement: Supplementary file 1 [file NIHPP2024.10.04.615514v2-supplement-3.pdf]

bioRxiv preprint doi: https://doi.org/10.1101/2024.10.04.615514; this version posted October 16, 2025. The copyright holder for this preprint (which was not certified by peer review) is the author/funder, who has granted bioRxiv a license to display the preprint in perpetuity. It is made available under aCC-BY 4.0 International license.

Supplementary Information

Different definitions of the *PABPN1* locus

In the hg38 reference, the *PABPN1* locus locus begins with six GCGs (chr14:23,321,473 - 23,321,490), followed by three GCAs (chr14:23,321,491 - 23,321,499), followed by GCG·GGG·GCT·GCG. This sequence can be concisely represented as GCG[6]GCA[3]GCG[1]GGG[1]GCT[1]GCG[1]. Brais et al. (1998)[51] established that *PABPN1* expansions to 8 or more GCG repeats cause autosomal dominant oculopharyngeal muscular dystrophy (OPMD). Their study did not include the adjacent GCA[3]GCG[1] in the estimation of the pathogenic threshold, and thereby set locus boundaries at chr14:23,321,473 - 23,321,490. However, subsequent studies[7,52] and resources such as GeneReviews and STRchive[29] extended the boundaries to chr14:23,321,473 - 23,321,499, including GCA[3]GCG[1], and therefore adjusted the pathogenic threshold to 12 or more repeats. In the meantime, other resources such as the STRipy database[53] and gnomAD continued to use Brais et al.'s original locus boundaries and pathogenic threshold of 8. While these different definitions describe the same biological reality, the inconsistency creates the potential for inadvertent errors in OPMD diagnosis.

bioRxiv preprint doi: https://doi.org/10.1101/2024.10.04.615514; this version posted October 16, 2025. The copyright holder for this preprint (which was not certified by peer review) is the author/funder, who has granted bioRxiv a license to display the preprint in perpetuity. It is made available under aCC-BY 4.0 International license.

Supplementary Figures

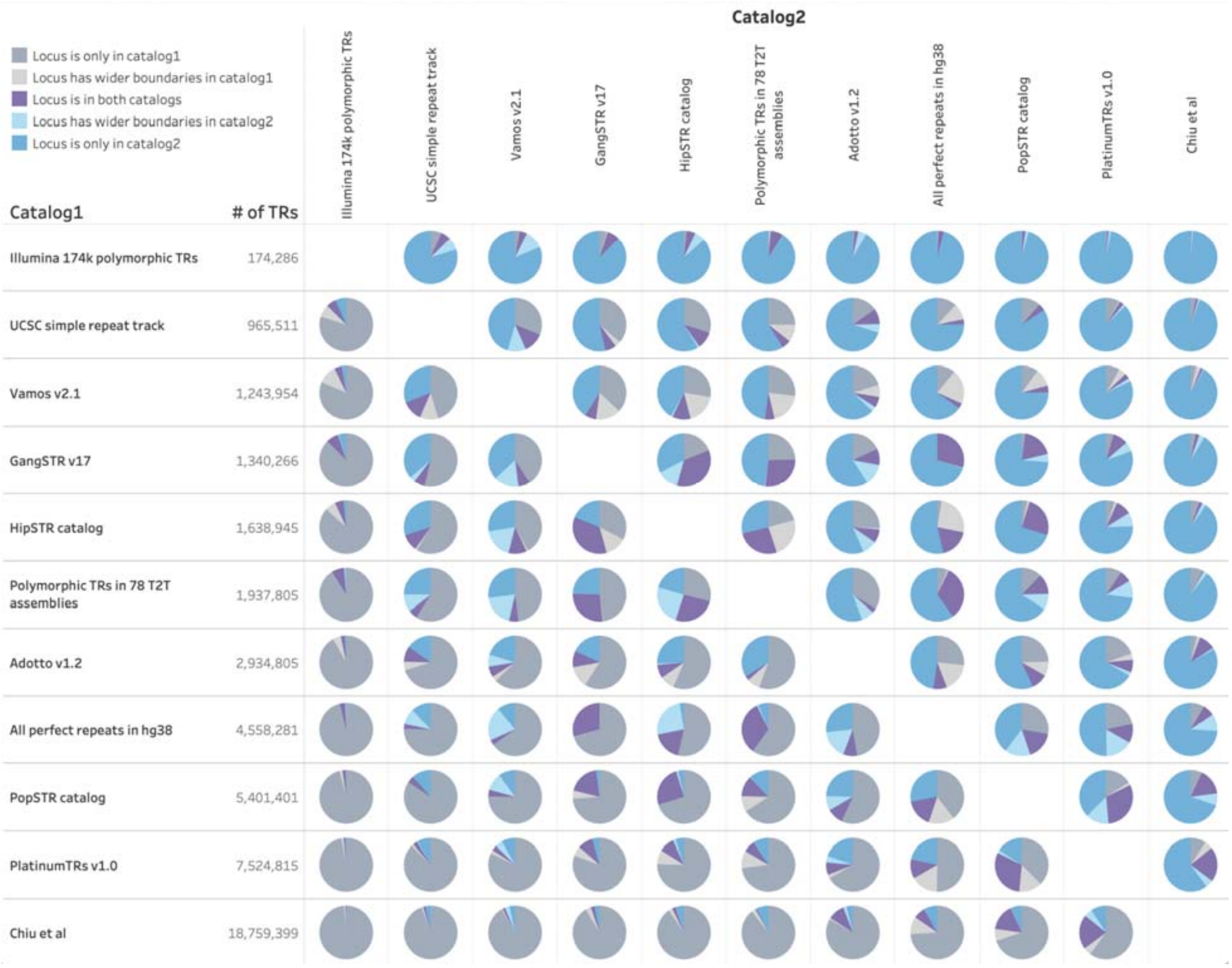

Supplementary Figure 1: Pairwise comparison of TR definitions in 11 catalogs. A locus was considered to be in both catalogs (purple) if the two locus definitions overlapped each other by at least 5%, specified the same motif, and differed in size by less than one motif length. Motif cyclic shifts and reverse complements (ie. CAG, AGC, GAC, CTG, TGC, GCT) were considered to be equivalent. Locus definitions with the same motif but wider boundaries in catalog 1 (light gray) or wider boundaries in catalog 2 (light blue) were counted separately.

bioRxiv preprint doi: https://doi.org/10.1101/2024.10.04.615514; this version posted October 16, 2025. The copyright holder for this preprint (which was not certified by peer review) is the author/funder, who has granted bioRxiv a license to display the preprint in perpetuity. It is made available under aCC-BY 4.0 International license.

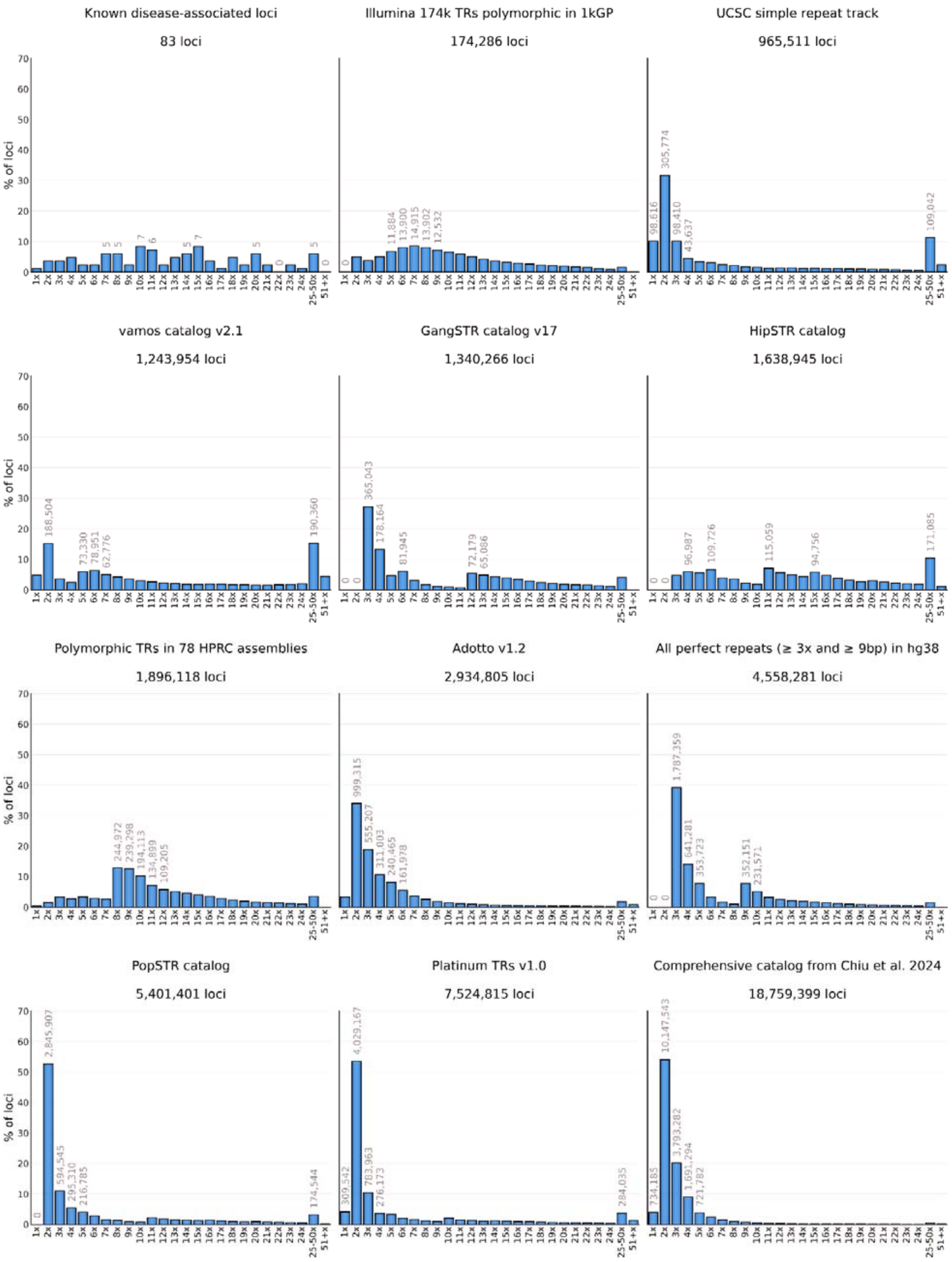

bioRxiv preprint doi: https://doi.org/10.1101/2024.10.04.615514; this version posted October 16, 2025. The copyright holder for this preprint (which was not certified by peer review) is the author/funder, who has granted bioRxiv a license to display the preprint in perpetuity. It is made available under aCC-BY 4.0 International license.

Supplementary Figure 2: Reference locus size distributions in 12 existing TR catalogs. The horizontal axis represents the repeat copy number of each locus in the hg38 reference genome.

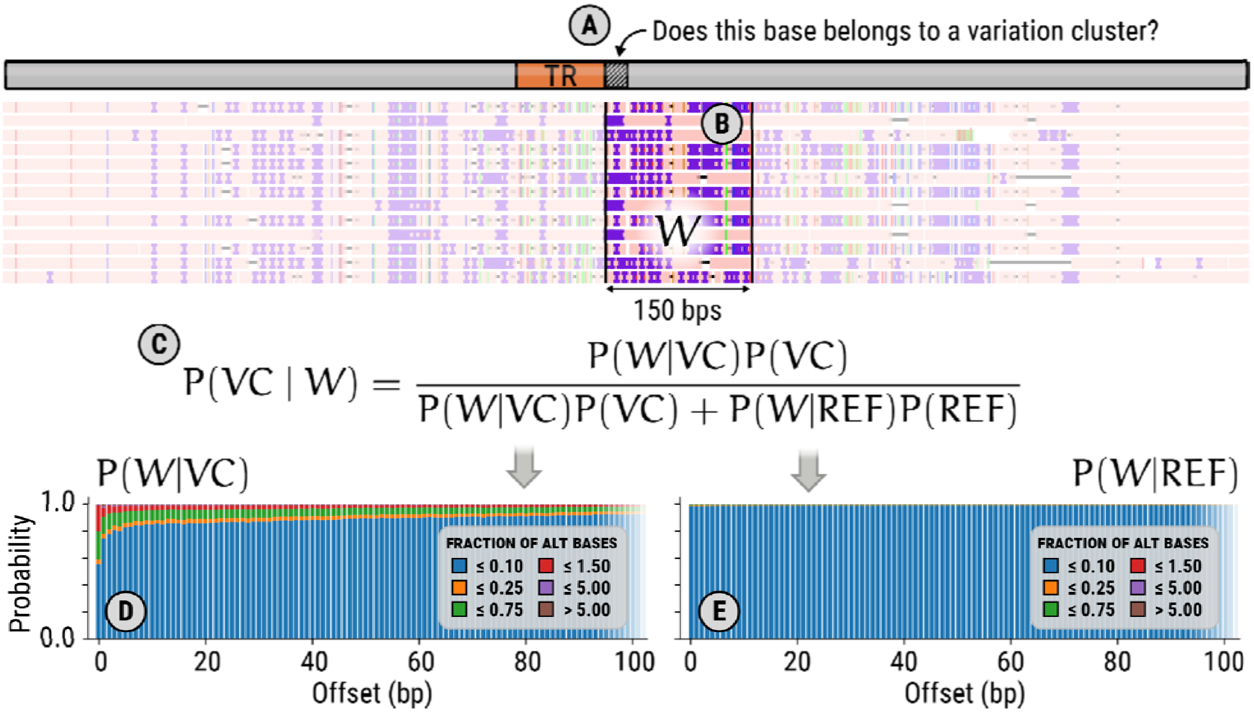

Supplementary Figure 3: Computing variation cluster boundaries. (A) The base being evaluated is adjacent to the tandem repeat. (B) The 150bp variation window that starts at the base under evaluation and extends away from the repeat. (C) The equation used to evaluate the probability that the first base of the window belongs to a variation cluster. (D) The model used to evaluate . (E) The model used to evaluate .

bioRxiv preprint doi: https://doi.org/10.1101/2024.10.04.615514; this version posted October 16, 2025. The copyright holder for this preprint (which was not certified by peer review) is the author/funder, who has granted bioRxiv a license to display the preprint in perpetuity. It is made available under aCC-BY 4.0 International license.

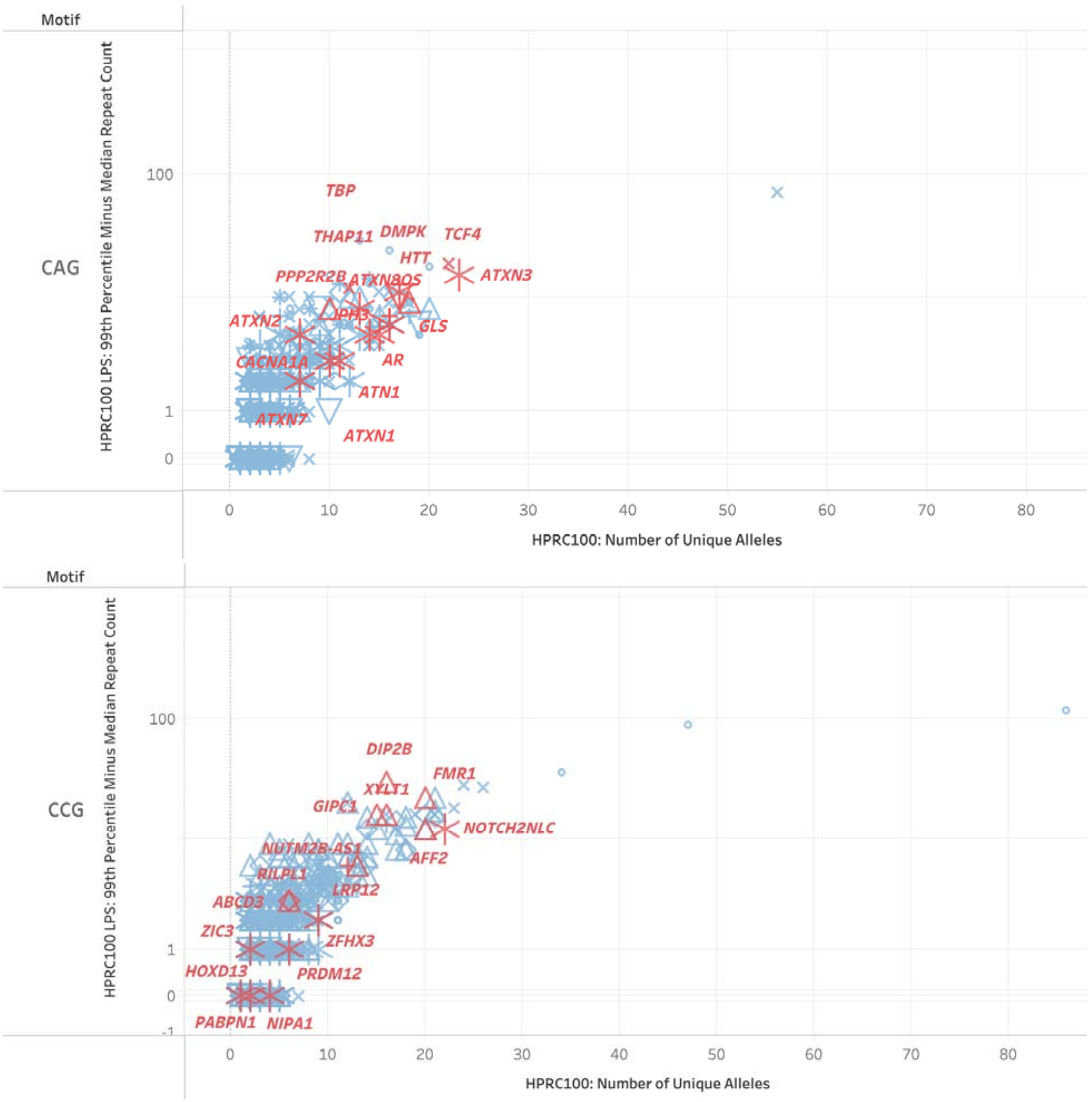

bioRxiv preprint doi: https://doi.org/10.1101/2024.10.04.615514; this version posted October 16, 2025. The copyright holder for this preprint (which was not certified by peer review) is the author/funder, who has granted bioRxiv a license to display the preprint in perpetuity. It is made available under aCC-BY 4.0 International license.

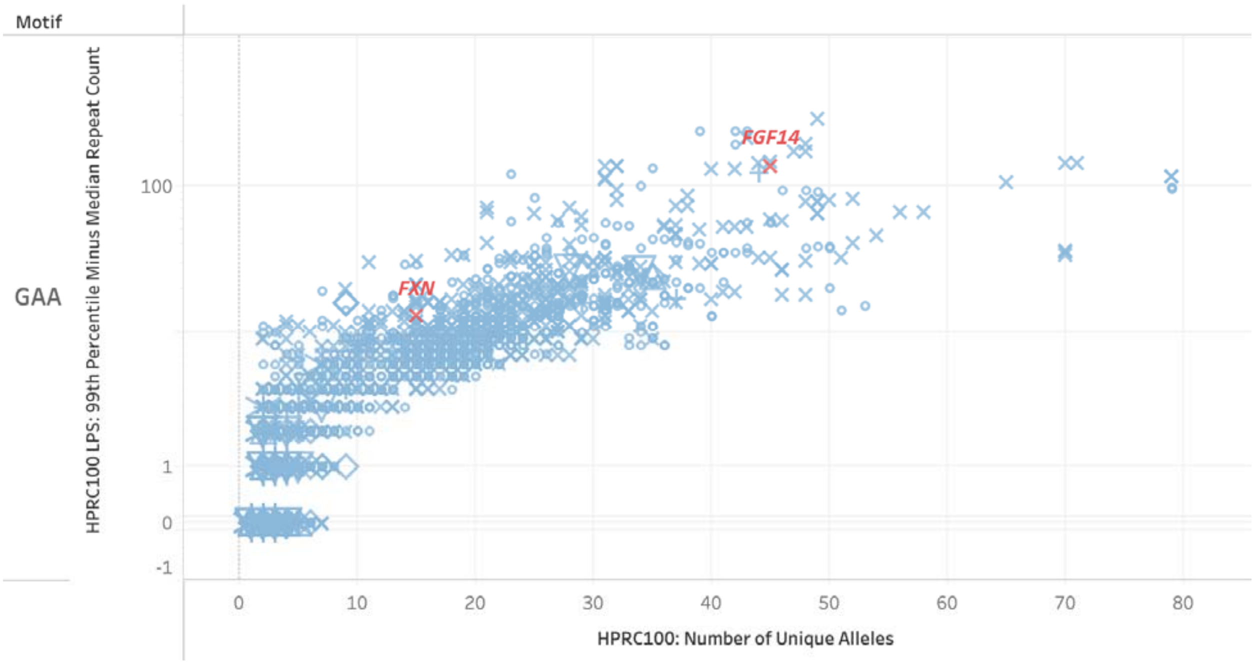

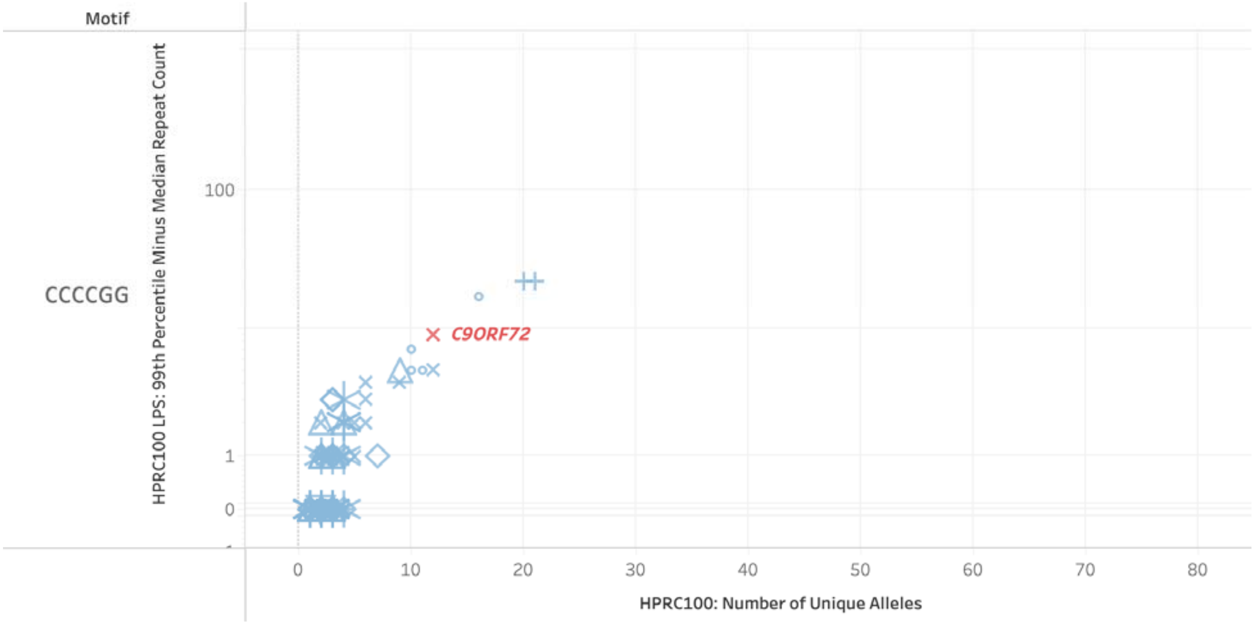

bioRxiv preprint doi: https://doi.org/10.1101/2024.10.04.615514; this version posted October 16, 2025. The copyright holder for this preprint (which was not certified by peer review) is the author/funder, who has granted bioRxiv a license to display the preprint in perpetuity. It is made available under aCC-BY 4.0 International license.

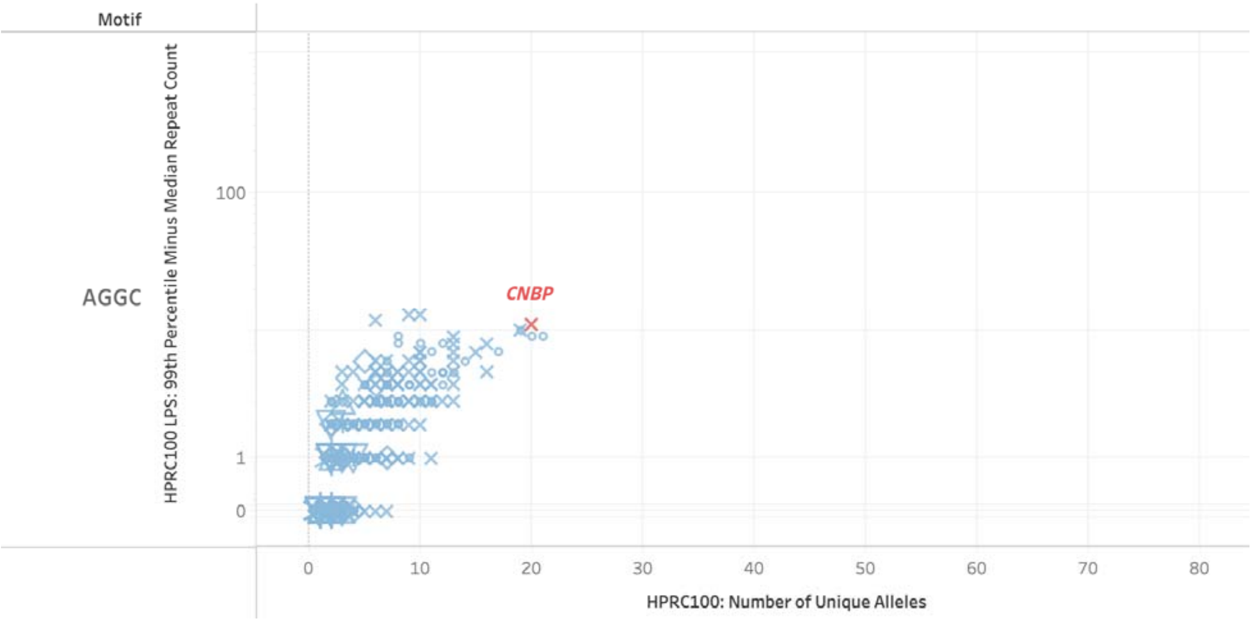

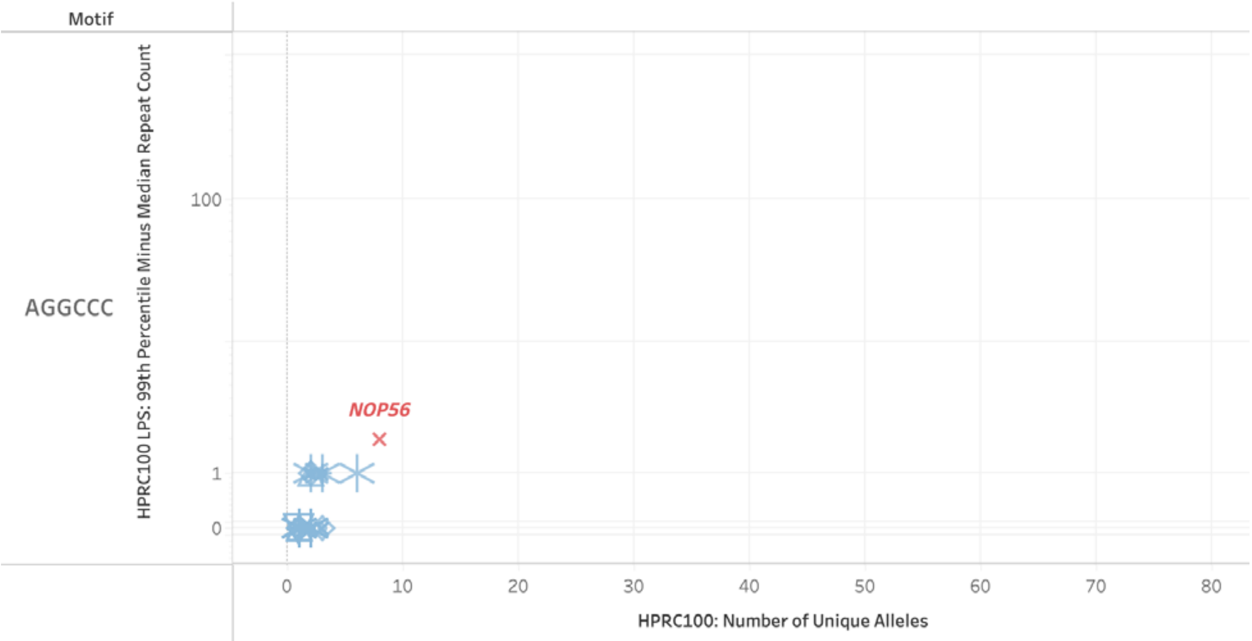

bioRxiv preprint doi: https://doi.org/10.1101/2024.10.04.615514; this version posted October 16, 2025. The copyright holder for this preprint (which was not certified by peer review) is the author/funder, who has granted bioRxiv a license to display the preprint in perpetuity. It is made available under aCC-BY 4.0 International license.

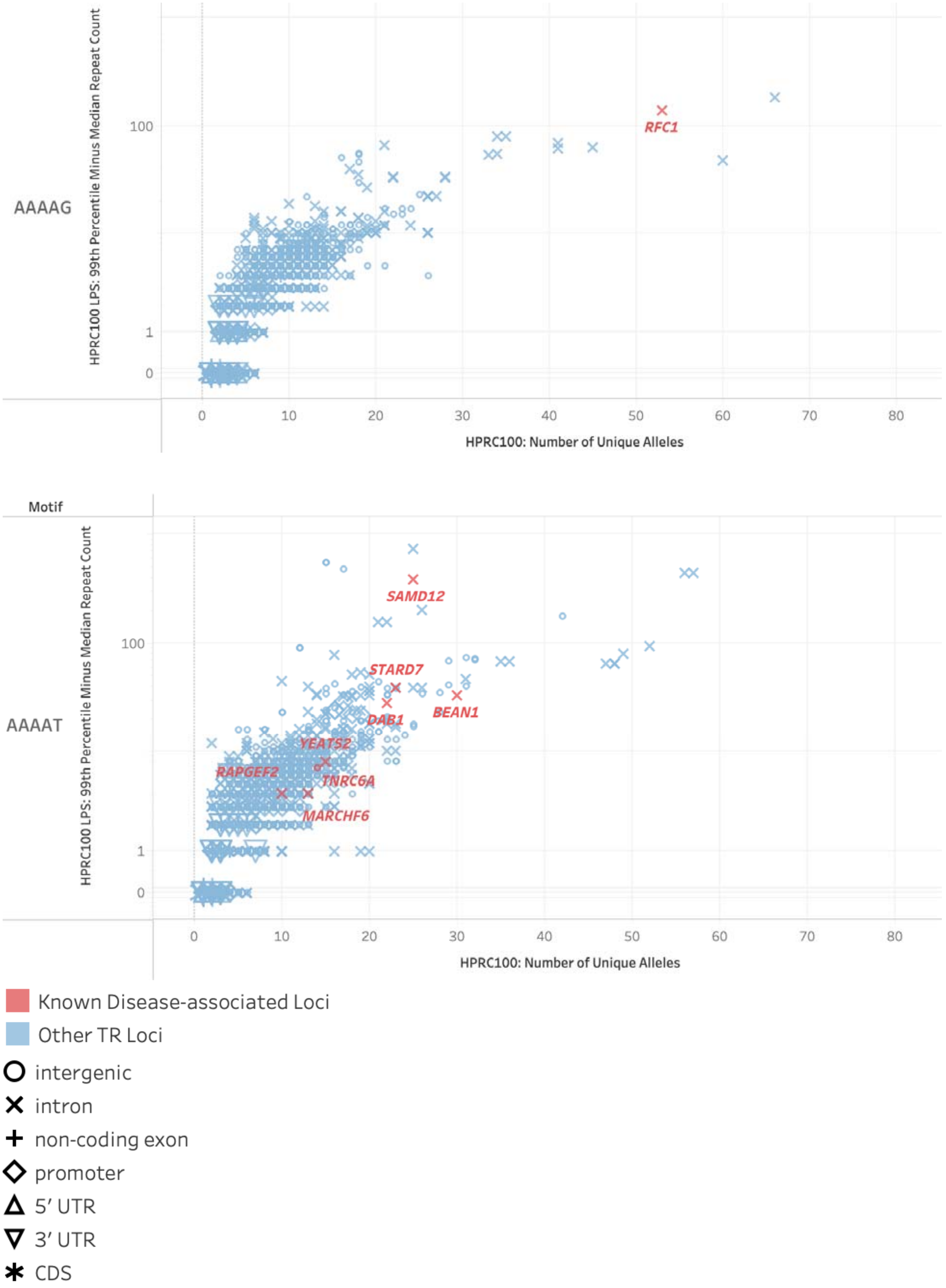

bioRxiv preprint doi: https://doi.org/10.1101/2024.10.04.615514; this version posted October 16, 2025. The copyright holder for this preprint (which was not certified by peer review) is the author/funder, who has granted bioRxiv a license to display the preprint in perpetuity. It is made available under aCC-BY 4.0 International license.

Supplementary Figure 4: Comparison of locus polymorphism rates stratified by motif. Comparison of known disease-associated STR loci with all other loci in the catalog that share the same normalized motif (where shifted or reverse-complemented motifs such as CAG, AGC, GCA, TGC, etc. are considered to be the same). Each panel shows data for a different STR motif: (A) CAG (B) CCG (C) GAA (D) CCCCGG (E) AGGC (F) AGGCCC (G) AAAAG (H) AAAAT. Two metrics of polymorphism are shown on the x- and y- axis: the x-axis shows the number of observed TR allele sizes at a locus, while the y-axis shows the 99th percentile allele size minus the median allele size (in units of repeat counts) at a locus. The color is red for known disease-associated loci and blue for all other loci. Different shapes represent the gene region of each locus in Gencode v46. The polymorphism metrics were generated by running TRGT-LPS with the full TR catalog on 100 long-read HPRC samples from diverse populations.

Supplementary Tables

Supplementary Table 1: Per-locus annotations provided with the catalog. Some annotations such as GencodeGeneName are only available for a subset of TRs. The six annotation categories are: Basic locus properties derived from the catalog itself or the reference genome (green), mappability (orange), variation clusters (blue), whether this is a known disease-associated locus (red), gene annotations (yellow), locus variability and population allele frequencies (purple).

bioRxiv preprint doi: https://doi.org/10.1101/2024.10.04.615514; this version posted October 16, 2025. The copyright holder for this preprint (which was not certified by peer review) is the author/funder, who has granted bioRxiv a license to display the preprint in perpetuity. It is made available under aCC-BY 4.0 International license.

Supplementary Table 2: Summary of annotations at known disease-associated loci. This table lists the hg38 start (0-based) and end coordinates, motifs, gene regions based on Gencode v46, polymorphism rates (computed as the standard deviation of the TRGT-LPS repeat size distribution in 100 long-read samples from the HPRC), average mappability of the locus and flanking regions, whether the locus is embedded within a larger variation cluster (and if yes, how much larger it is than the TR locus), and finally, the number of other TR loci near this locus.
